# Light‐Activated Isolation of High‐Quality Mitochondria for Therapeutic Transplantation

**DOI:** 10.1002/anie.7935890

**Published:** 2026-05-28

**Authors:** Hui Liu, Yuxin Jiao, Ting Zhang, Haiwei Wang, Yufei Xue, Jiayu Ding, Yang Ding, Meiling Wang, Weisen Zhang, Hua Bai, Bo Peng, Nicolas H. Voelcker, Lin Li

**Affiliations:** ^1^ State Key Laboratory of Flexible Electronics (LOFE) & Institute of Flexible Electronics (IFE) Northwestern Polytechnical University Xi'an China; ^2^ Drug Delivery Disposition and Dynamics Monash Institute of Pharmaceutical Sciences Monash University Parkville Australia; ^3^ State Key Laboratory of Flexible Electronics (LoFE) & Institute of Flexible Electronics (IFE) Xiamen University Xiamen China

**Keywords:** ischemia‐reperfusion injury, mitochondrial isolation, mitochondrial transplantation, multifunctional modular design, photo‐responsive release

## Abstract

Artificial mitochondrial transplantation (AMT) holds great promise for reprogramming cellular metabolism and restoring cell function. Its clinical translation, however, relies on access to mitochondria that are both of high purity and metabolically active, requirements that current isolation techniques struggle to meet. Conventional differential centrifugation (DC) method yields heterogeneous and low‐activity mitochondria, whereas magnetic bead (MB)‐based immuno‐isolation leaves non‐biodegradable beads permanently attached. Herein, we present a Light‐Activated Mitochondrial Isolation (**LAMI**) platform comprising programmable mitochondria‐targeting MBs and a photo‐responsive release mechanism for the selective, efficient, and non‐destructive extraction of high‐quality mitochondria. **LAMI** employs magnetic nanoparticles decorated with a branched, modular probe architecture that supports systematic variation in mitochondria‐targeting ligand type, ligand density, and optical tracking elements. Incorporation of a photo‐cleavable linker allows on‐demand, mild, and reagent‐free release of captured mitochondria. Compared with DC method, **LAMI** produces mitochondria with markedly improved purity, structural integrity, and functionality. In an ischemia‐reperfusion injury (IRI) model, **LAMI**‐isolated mitochondria‐based AMT exhibits superior therapeutic performance. Together, **LAMI** provides a non‐destructive, efficient, and versatile mitochondrial isolation strategy that overcomes long‐standing limitations of current methods, offering a robust platform to advance AMT and its future biomedical applications.

## Graphical abstract

The Light‐Activated Mitochondrial Isolation (**LAMI**) platform enables the selective and non‐destructive isolation of high‐purity, metabolically active mitochondria. By integrating modular, ligand‐programmable magnetic probes with photo‐cleavable release, **LAMI** overcomes long‐standing limitations of conventional isolation methods and provides a robust foundation for artificial mitochondrial transplantation (AMT).

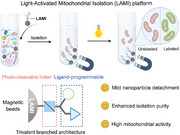

## 1 Introduction

Mitochondria are essential subcellular organelles responsible for cellular energy production and functional maintenance, and their dysfunction is directly associated with the progression of critical illnesses [1, 2]. Recent studies have revealed that mitochondria are dynamic and mobile, capable of transferring between cells [3]. Among various intercellular transfer modes, the direct delivery of isolated mitochondria, termed artificial mitochondrial transplantation (AMT), has emerged as a promising therapeutic approach [4, 5, 6]. Unlike cell‐mediated or extracellular vesicle (EV)‐based delivery, AMT enables precise control over mitochondrial purity, functionality, and dosage [7]. Transplanted mitochondria rapidly replenish ATP and metabolic intermediates to restore energy metabolism, while improving mitochondrial quality control *via* enhanced dynamics and autophagy [8]. Meanwhile, AMT also remodels intracellular signaling by modulating reactive oxygen species (ROS) and calcium homeostasis [9, 10, 11], and ultimately influences nuclear gene expression through the metabolic‐epigenetic axis, thereby achieving profound cell fate reprogram [12]. To date, AMT has been investigated in clinical trials for cardiac ischemia‐reperfusion injury (IRI) and has demonstrated remarkable therapeutic efficacy in various preclinical models of critical diseases, including ischemia‐reperfusion injuries, spinal cord injury, cardiac resuscitation, anti‐aging, and inherited mitochondrial diseases [13, 14, 15].

**SCHEME 1:**
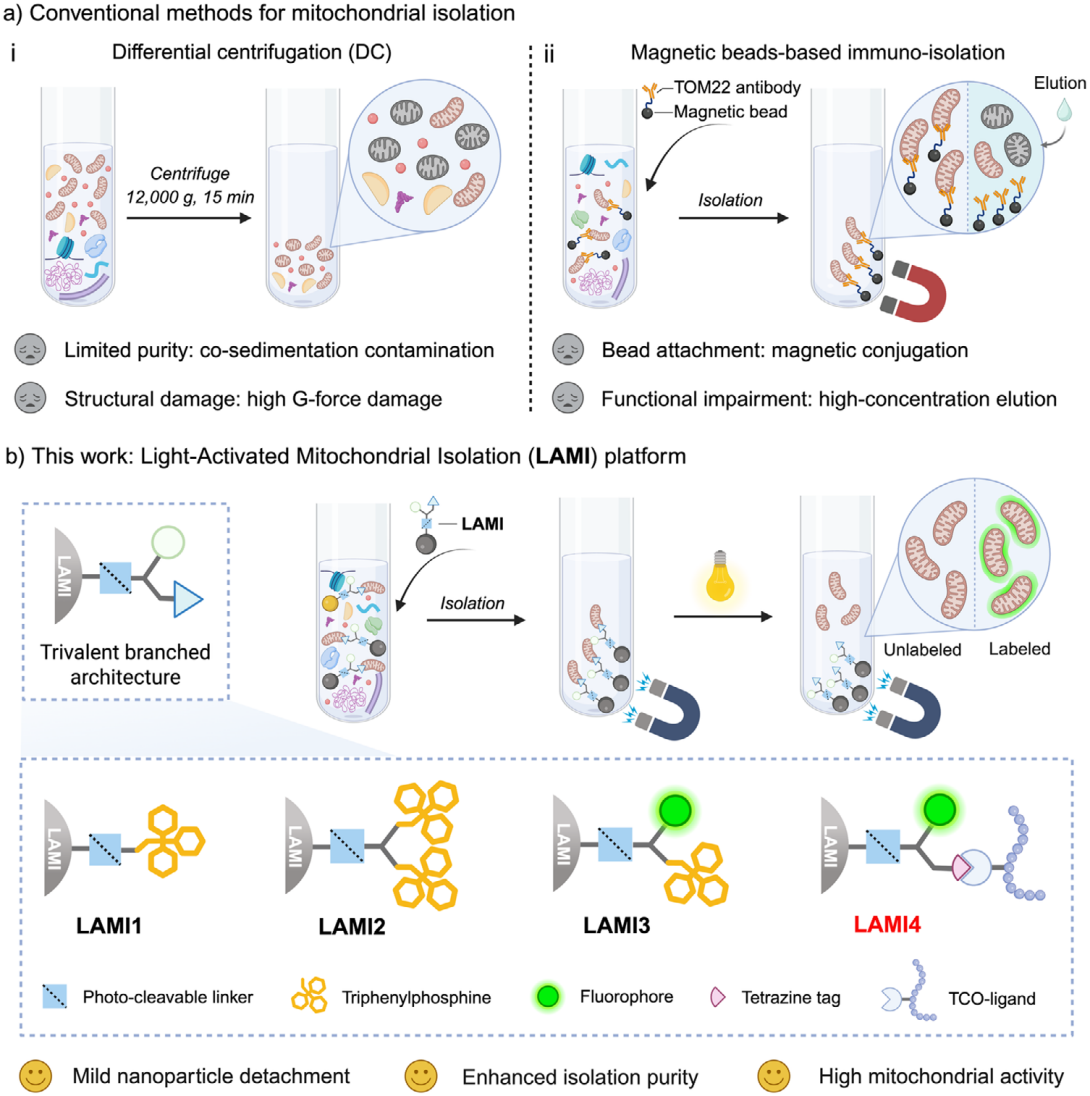
Schematic representation of mitochondrial isolation strategies. (a) Conventional methods for mitochondrial isolation. (i) differential centrifugation (DC) method, (ii) magnetic bead (MB)‐based immuno‐isolation. (b) This work: the Light‐Activated Mitochondrial Isolation (**LAMI**) platform for the selective capture and rapid yet gentle release of functional mitochondria.

However, the clinical translation of AMT critically depends on obtaining large quantities of high‐purity and functionally competent mitochondria from donor cells [16, 17]. Existing isolation methods pose significant technical and economic challenges (Scheme 1a). Differential centrifugation (DC), the most widely used method [18], inherently yields mitochondria of limited purity because mitochondria co‐sediment with similarly sized cellular debris and other organelles, resulting in heterogeneous activity and variable functional quality [19, 20, 21]. Additionally, the high gravitational forces involved can inflict mechanical damage on mitochondrial structures, further diminishing the functional integrity of the isolate [22, 23]. Magnetic bead (MB)‐based immuno‐isolation, by attaching antibodies such as anti‐TOM22 to the surface of MBs, improves purity and is highly effective for downstream analytical applications; however, it introduces non‐biodegradable beads that irreversibly attach to mitochondrial membranes, raising biosafety concerns for in vivo use, particularly in the context of AMT [24, 25, 26, 27]. Alternative elution strategies offer partial solutions, yet require high concentrations of elution agents such as imidazole, which can impair mitochondrial activity, further limiting their clinical applicability [28]. These limitations highlight the need for an isolation platform that not only enables gentle and traceless mitochondrial release, but also allows systematic optimization of targeting design to balance capture efficiency, purity, and functional preservation.

To address these limitations, in this work, we developed the Light‐Activated Mitochondrial Isolation platform (**LAMI**, Scheme 1b). At its core, **LAMI** features a modular, ligand‐programmable architecture tailored specifically for non‐destructive organelle isolation. Specifically, **LAMI** is built upon a set of rationally engineered mitochondrial probes (**ZT1**–**ZT4**) anchored on magnetic nanoparticles, incorporating a trivalent branched architecture that allows systematic tuning of mitochondrial‐targeting ligands, such as triphenylphosphonium (TPP^+^) and mitochondria‐penetrating peptides (MPPs). This modular design enables exploration of different targeting strategies and identification of the most effective probe configuration. In addition, the probes incorporate photo‐cleavable linkers, enabling mitochondria to be captured selectively and released gently upon light activation. Among the four probes, **ZT4**, which is functionalized with MPPs, exhibited the highest performance, yielding mitochondria with superior purity and functional integrity. The functional competence of **LAMI**‐isolated mitochondria was further validated in an in vitro hepatic IRI model. Mitochondria purified using **LAMI** demonstrated markedly enhanced therapeutic efficacy relative to those obtained by DC method. Collectively, **LAMI** provides an optimized approach for efficient mitochondrial isolation and transplantation, with strong potential to accelerate the clinical translation of AMT.

## 2 Result and Discussion

### 2.1 Design and Synthesis of Mitochondrial Probes

Photo‐cleavable linkers offer distinct advantages for on‐demand regulation, remote control, and non‐invasive operation. Among them, *o*‐nitrobenzyl (ONB) groups undergo efficient cleavage under 395 nm irradiation and have been widely applied in drug delivery and optogenetics (Figure S1) [29]. Leveraging this property, we incorporated ONB linkers into the **LAMI** platform to enable robust yet gentle disassociation of mitochondria from MBs. Prior to constructing **LAMI**, a family of ONB‐based probes (**ZT1**–**ZT4**) with modular functionalities were rationally designed and synthesized, including tunable targeting group density (**ZT1**–**ZT2**), fluorescence tracking capability (**ZT3**–**ZT4**), and a tetrazine (Tz) tag for efficient and versatile ligand conjugation (**ZT4**).

**FIGURE 1:**
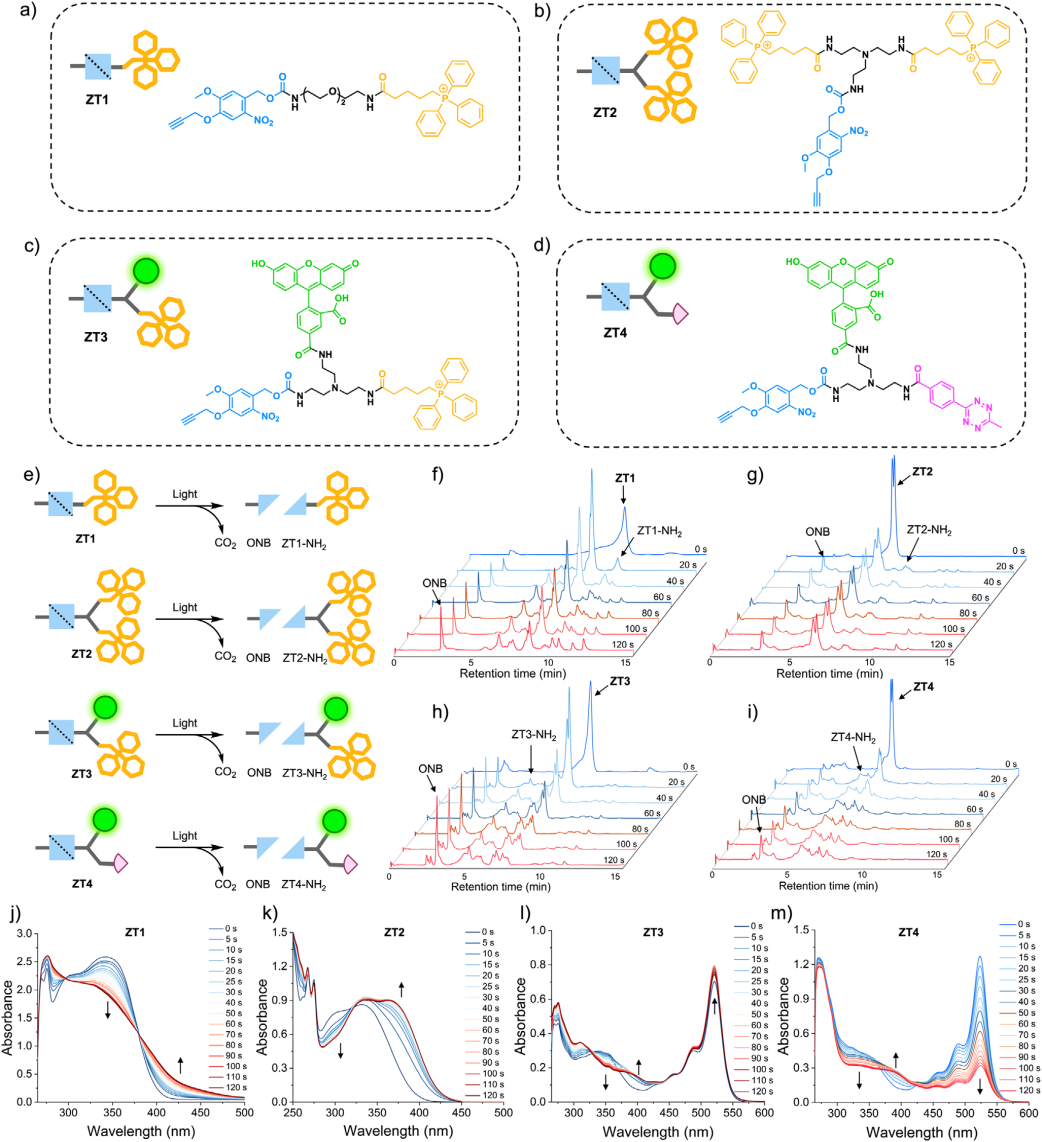
Photolysis mechanism and controllable photo‐responsiveness of the multifunctional probes. (a–e) Molecular structures of **ZT1**–**ZT4** and their photolysis products. (f–i) Liquid chromatography‐mass spectrometry (LC‐MS) analysis showing the time‐dependent bond cleavage of **ZT1**–**ZT4**. (j–m) Ultraviolet–visible (UV–Vis) absorption spectra indicating the progressive bond cleavage of **ZT1**–**ZT4** upon irradiation. All photolysis experiments were performed using an OmniCure S2000 (395 nm, 5 mW cm^−2^, 0.6 J cm^−2^).

As outlined in Schemes S1 and S2 (Supporting Information), the synthetic route of the probes began with vanillin, from which the ONB linker (A4) was prepared through sequential protection, nitration, deprotection, and activation steps. This ONB linker was then coupled to TPP^+^ via a polyethylene glycol (PEG) spacer to yield **ZT1** (Figure 1a). To explore how multivalency influences mitochondrial capture efficiency, a trivalent probe architecture was introduced by coupling the photo‐cleavable linker to a tris(2‐aminoethyl)amine scaffold, generating a three‐armed intermediate (CA1). The two branches of CA1 were conjugated with TPP^+^ to yield **ZT2** (Figure 1b). Building on this scaffold, replacing one TPP^+^ group in **ZT2** with 5‐carboxyfluorescein (5‐FAM) afforded **ZT3**, enabling simultaneous targeting and fluorescence tracking (Figure 1c). To enable more versatile ligand conjugation, the remaining TPP^+^ in **ZT3** was substituted with a bioorthogonal handle (i.e., Tz moiety), giving rise to **ZT4** (Figure 1d). This design allowed efficient conjugation of diverse targeting ligands via Tz–trans‐cyclooctene (TCO) bioorthogonal click chemistry. The successful synthesis of **ZT1**–**ZT4** and all their intermediates was confirmed by ^1^H and ^13^C NMR spectroscopy as well as high‐resolution mass spectrometry (HRMS) (Figures S21–S41).

The photolytic properties of **ZT1**–**ZT4** were evaluated using liquid chromatography‐mass spectrometry (LC‐MS). Upon irradiation with 395 nm UV light (5 mW cm^−2^), all probes decomposed within 2 min (0.6 J cm^−2^), producing the expected cleavage molecules, demonstrating their high photosensitivity (Figure 1e–i). Ultraviolet–visible (UV–Vis) absorption spectra revealed a consistent trend in the range from 250 to 450 nm as irradiation time increased, characterized by a progressive decrease of the parent absorption band and the concomitant emergence of photolysis‐related features (as indicated in Figure 1j–m), further confirming efficient ONB cleavage. In the 450–600 nm range of the absorption spectra, the absorption profiles of **ZT3** and **ZT4** at different concentrations exhibited spectral features similar to those of the free 5‐FAM fluorophore, confirming the fluorescent properties of **ZT3** and **ZT4** (Figure S2a–c). Compared with the maximum absorption peak of 5‐FAM at 470 nm, the absorption maximum of the FAM moiety in **ZT3** and **ZT4** are red‐shifted to 525 nm. This is primarily attributed to the π‐conjugation extension and the enhanced intramolecular charge transfer (ICT) effect resulting from structural modification [30, 31].

### 2.2 The Fabrication and Characterization of LAMI Platform

After confirming the controllable photo‐responsiveness of **ZT1**–**ZT4**, these probes were covalently conjugated to MBs to construct the **LAMI** beads. To establish the optimal surface functionalization strategy, a series of N_3_‐NHS linkers with varying alkyl chain lengths and PEG segments was first screened (1–5N_3_, Figure S3a). Amino‐functionalized MBs (MB‐NH_2_, 3.1 nmol mg^−1^) were conjugated with each linker under mild alkaline conditions to form azide‐functionalized beads. **ZT2** was used as a representative probe, and attachment of **ZT2** to the bead surface was achieved through CuI‐catalyzed azide–alkyne cycloaddition (CuAAC). Because most mitochondria‐targeting ligands rely on mitochondrial membrane potential (MMP) for effective interaction, zeta potential was used as the primary evaluation criterion. Beads modified with 2N_3_, (2,5‐dioxopyrrolidin‐1‐yl) 4‐azidobutanoate, exhibited the most favorable positive surface charge and were therefore selected for the preparation of all subsequent **LAMI** constructs (Figure S3b).

**FIGURE 2:**
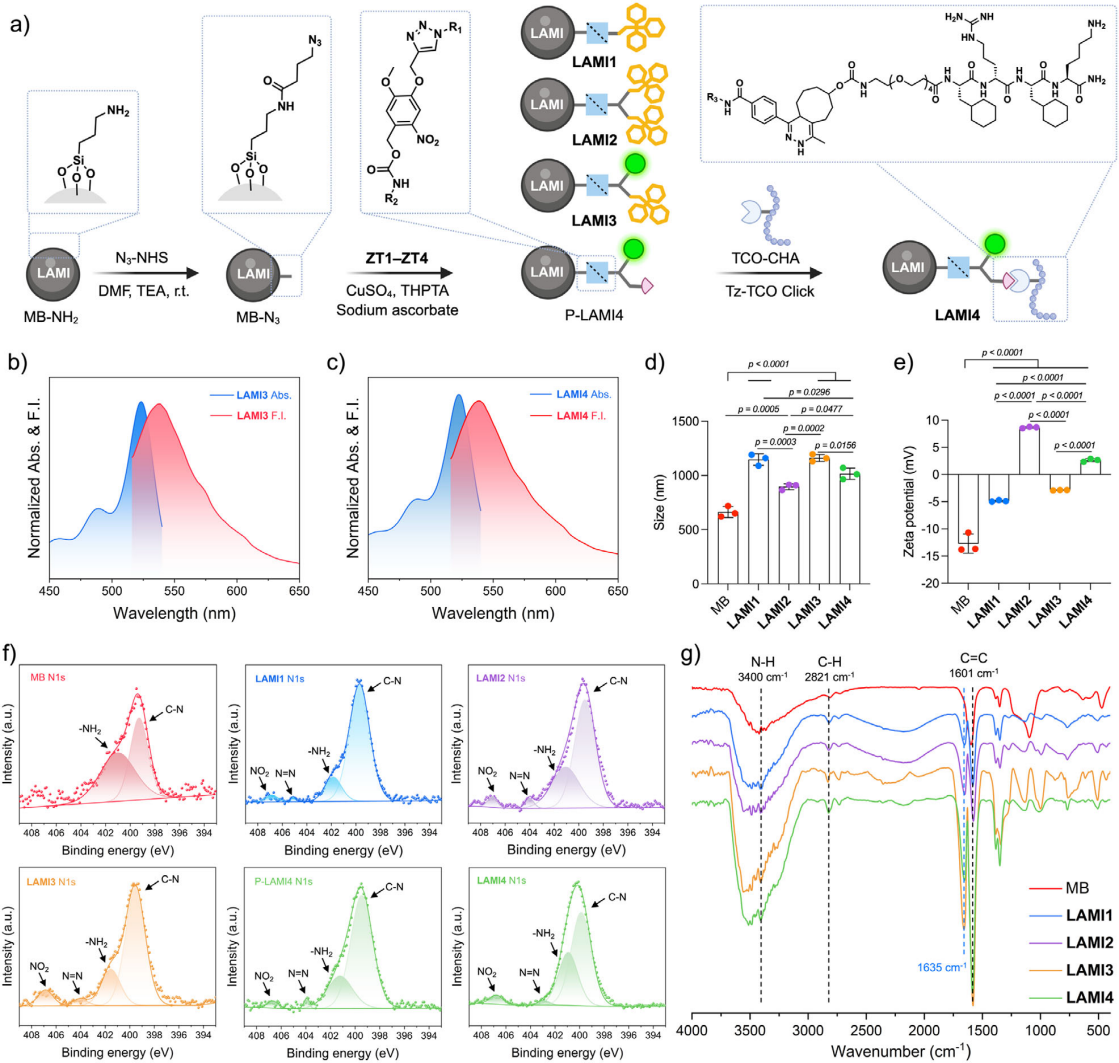
Construction and characterization of the **LAMI** platform. (a) Schematic of the **LAMI** assembly process. (b) Normalized absorption (within the 5‐FAM absorption range) and fluorescence emission spectra of **LAMI3** in DMF. (c) Normalized absorption (within the 5‐FAM absorption range) and fluorescence emission spectra of **LAMI4** in DMF. (d) Size distribution of **LAMI** beads measured by dynamic light scattering (DLS). (e) Zeta potential characterization of **LAMI** beads. (f) X‐ray photoelectron spectroscopy (XPS) analysis of **LAMI** beads. (g) Fourier‐transform infrared (FTIR) spectra of **LAMI** beads. The results are shown as the average values ± SD (*n* = 3). The *p* values were determined by one‐way ANOVA with Tukey's multiple comparison test.

The conjugation of **ZT1**–**ZT4** to 2N_3_‐modified beads generated the **LAMI** beads, that is, **LAMI1**–**LAMI3** and the precursor P‐LAMI4. P‐LAMI4 can be further extended via bioorthogonal Tz–TCO ligation with TCO‐functionalized targeting ligands (e.g., antibodies, aptamers, peptides). As a proof of concept, MPPs, a class of synthetic peptides known for efficient cellular uptake and mitochondrial localization [32], were employed. We synthesized a TCO‐modified MPP (TCO‐cyclohexylalanine‐arginine‐cyclohexylalanine‐lysine, TCO‐CHA, Scheme S2) and conjugated it to P‐LAMI4 to generate **LAMI4** (Figure 2a). The successful conjugation of **ZT3** and **ZT4** probes to the beads (i.e., **LAMI3** and **LAMI4**) was further confirmed by their characteristic fluorescence emission spectra (Figures 2b,c and S4a–c).

The morphology and size of the **LAMI** beads were subsequently characterized by scanning electron microscopy (SEM) and dynamic light scattering (DLS). SEM images revealed an average bead diameter of approximately 250 nm (Figure S5), comparable to bare MBs. In contrast, DLS analysis showed an increase in hydrodynamic diameter to ∼1 µm after surface modification, likely attributable to the hydrophobicity and intermolecular interactions of the conjugated probes (Figure 2d). Given the relatively large size of mitochondria (typically 0.5–2 µm), such clustering is expected to remain compatible with mitochondrial capture. After photo‐cleavage, the beads were removed by magnetic separation and were therefore absent from the final mitochondrial preparation. For zeta potential measurements, bare MBs exhibited a negative potential (–12.7 mV). After conjugation of **ZT1** (i.e., **LAMI1**), the potential increased to –4.8 mV owing to the presence of the cationic TPP^+^ group. **LAMI2**, functionalized with **ZT2** which contains two TPP^+^ moieties, displayed a potential of +8.6 mV, while **LAMI3** exhibited a similar potential to **LAMI1** due to replacement of one TPP^+^ with FAM. Upon conjugation with cationic TCO‐CHA, **LAMI4** displayed a zeta potential of +2.6 mV (Figure 2e).

Finally, fourier‐transform infrared (FTIR) and x‐ray photoelectron spectroscopy (XPS) analyses further confirmed the successful generation of **LAMI1**–**LAMI4**. The XPS spectra showed an increase in the N1s peak intensity, accompanied by the emergence of characteristic N=N and –NO_2_ signals, consistent with the nitrogen‐rich structures of **ZT1**–**ZT4** (Figure 2f). Correspondingly, an increased proportion of C–O bonds was observed in the C1s spectrum (Figure S6). The FTIR spectra of all surface‐functionalized samples, along with the pristine MBs, exhibited characteristic absorption bands at 3400, 2821, and 1601 cm^−1^, corresponding to N–H, C–H, and C=C stretching vibrations, respectively, indicating the successful conjugation of **ZT1**–**ZT4** on the MB surface. Meanwhile, **LAMI** beads showed a distinct peak at 1635 cm^−1^ attributed to the C–H stretching vibration of the ONB group, and their fingerprint region features were also consistent with those of the **ZT1**–**ZT4** molecules (Figures 2g and S7).

### 2.3 Verification of LAMI Platform for Efficient and Pure Mitochondrial Isolation

**FIGURE 3:**
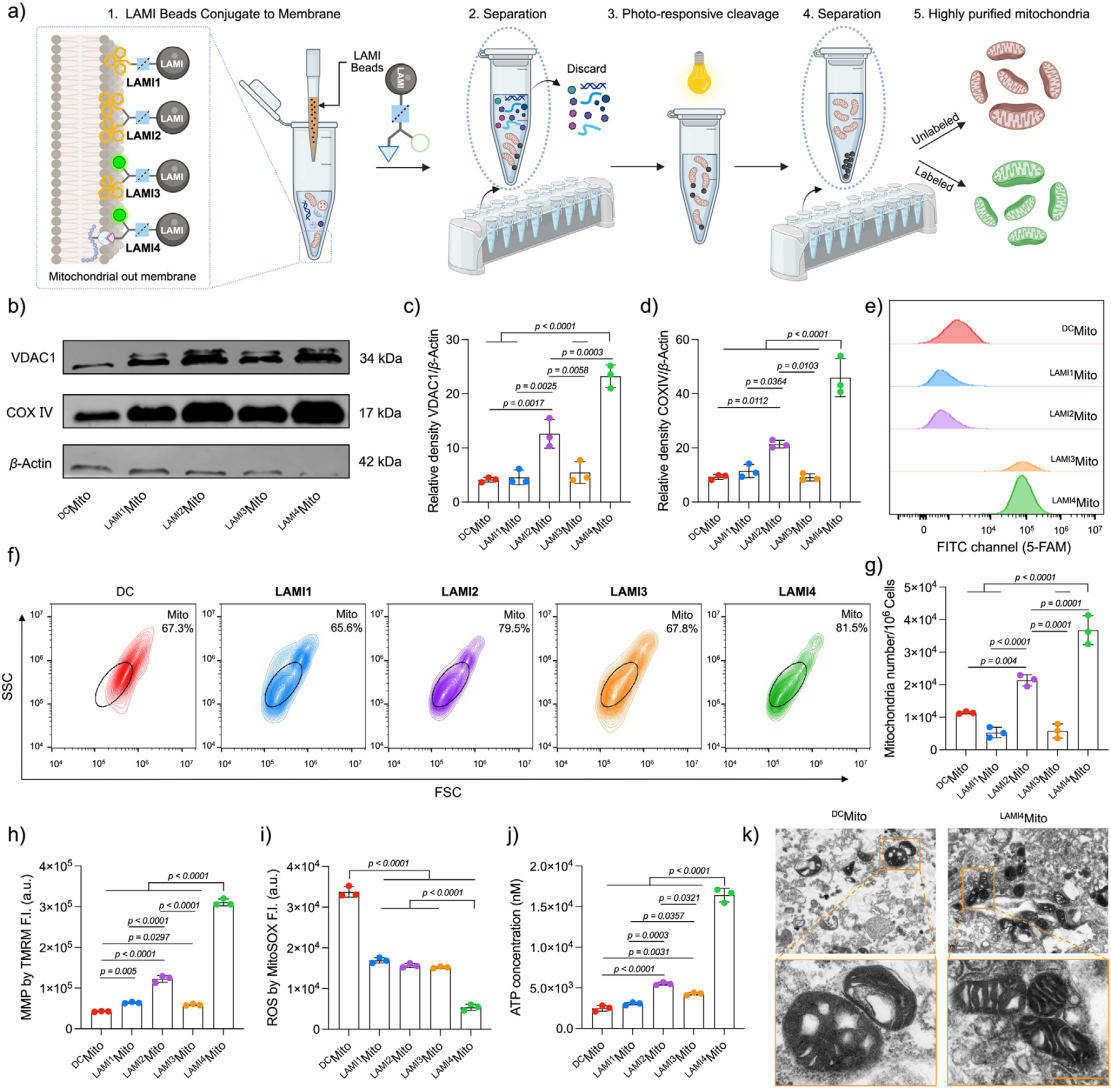
Verification of **LAMI** platform for efficient and pure mitochondrial isolation. (a) Schematic workflow of **LAMI**‐mediated isolation of mitochondria from wild‐type HepG2 cells. (b–d) Expression levels and relative quantification of voltage‐dependent anion channel 1 (VDAC1) and cytochrome‐c oxidase subunit IV (COX IV) in isolated mitochondria. The corresponding uncropped images are available in Figure S20 in the Supporting Information. (e) Fluorescence‐activated cell sorting (FACS) analysis of FITC fluorescence detection channels of mitochondria extracted by the DC and **LAMI**‐based method, respectively. (f) FACS scatter plots of mitochondria isolated using the DC and **LAMI**‐based method, respectively. (g) Quantification of mitochondrial particle numbers within the FACS gate for equal sample volumes. (h–j) The membrane potential (MMP) (h), ROS level (i) and ATP content (j) of mitochondria were measured in samples following isolation with the DC and **LAMI**‐based method, respectively. (k) Bio‐transmission electron microscopy (bio‐TEM) images of ^DC^Mito and ^LAMI4^Mito, scale bar = 500 nm. The results are shown as the average values ± SD (*n* = 3 independent biological replicates). The *p* values were determined by one‐way ANOVA with Tukey's multiple comparison test.

To evaluate the efficiency of the **LAMI** platform, mitochondria were isolated and purified from wild‐type HepG2 cells using either the DC (^DC^Mito) or **LAMI** (^LAMI^Mito). For ^DC^Mito, mitochondria were isolated using a commercial kit (Supporting Information). For the ^LAMI^Mito, **LAMI** beads were incubated with the cell lysates, followed by magnetic separation to remove the cytosolic fraction and washing under a magnetic field. Finally, the **LAMI** beads were irradiated with 395 nm light at a power density of 5 mW cm^−2^ for 2 min (total fluence: 0.6 J cm^−2^) to release the mitochondria from the MBs (Figure 3a). This process was verified by bio‐scanning electron microscopy (bio‐SEM, Figure S8). After mitochondrial isolation and purification, the size and zeta potential of the mitochondria were characterized. The results indicated that mitochondria isolated by the **LAMI**‐based method exhibited similar physical properties to those isolated by the DC method (Figure S9). Furthermore, the biocompatibility of the 395 nm irradiation used for photo‐triggered release was evaluated using mitochondria isolated by the DC method. No significant differences were observed between irradiated and non‐irradiated mitochondria in ROS levels, MMP, or ATP production (Figure S10), indicating that these irradiation conditions do not measurably impair mitochondrial function.

The identity and purity of the isolated mitochondria were further assessed by western blotting (WB) using voltage‐dependent anion channel 1 (VDAC1) and cytochrome‐c oxidase subunit IV (COX IV) as mitochondrial markers. *β*‐Actin was used as a cytosolic contamination marker; therefore, the ratios of VDAC1/*β*‐actin and COX IV/*β*‐actin served as indicators of mitochondrial purity. ^DC^Mito, ^LAMI1^Mito, and ^LAMI3^Mito showed comparable marker ratios; ^LAMI2^Mito yielded an approximately two‐fold increase of marker ratios than ^LAMI1^Mito and ^LAMI3^Mito, demonstrating that ligand density plays an important role in mitochondrial targeting and isolation. Notably, ^LAMI4^Mito demonstrated the highest enrichment, with VDAC1/*β*‐actin and COX IV/*β*‐actin ratios reaching nearly four‐fold those of ^DC^Mito (Figures 3b–d and S11), likely due to the lower mitochondrial capture efficiency of TPP^+^ compared to MPPs. These results confirm that the **LAMI** platform, particularly the **LAMI4**, achieves significantly more efficient and higher‐purity mitochondrial isolation than conventional DC method.

Subsequently, fluorescence‐activated cell sorting (FACS) in the FITC channel showed a clear rightward fluorescence intensity shift for ^LAMI3^Mito and ^LAMI4^Mito, confirming successful 5‐FAM labeling of the isolated organelles (Figure 3e). FACS analysis was further employed to quantify mitochondrial purity. Among all samples, ^LAMI4^Mito exhibited the highest average purity (77.8%), followed by ^LAMI2^Mito (72.3%). In comparison, ^DC^Mito, ^LAMI1^Mito, and ^LAMI3^Mito displayed lower average purities of 63.5%, 65.3%, and 68.6%, respectively (Figures 3f and S12). Further quantification of mitochondrial particle counts in equal volumes of the concentrated samples revealed that ^LAMI4^Mito had the highest particle count, exceeding those of ^DC^Mito and ^LAMI1–LAMI3^Mito, nearly four‐fold those of ^DC^Mito (Figure 3g), further demonstrating improved mitochondrial isolation efficiency. Collectively, **LAMI** demonstrated improved mitochondrial enrichment and isolation efficiency compared to the DC method.

We next evaluated the functional integrity of the isolated mitochondria, which revealed major advantages of the **LAMI** platform. Compared with mitochondria isolated by DC method, all **LAMI** variants showed significantly improved preservation of mitochondrial function, as evidenced by higher MMP and lower levels of ROS (Figures 3h,i, S13, and S14). In terms of ATP production, while ^LAMI2–LAMI4^Mito displayed significantly elevated ATP levels, ^LAMI1^Mito displayed ATP levels comparable to ^DC^Mito (Figure 3j). This universal improvement can be attributed to the gentle, affinity‐based magnetic isolation process, which avoids the mechanical stress and shear forces associated with ultracentrifugation.

Beyond this general benefit, distinct differences were observed among the **LAMI** variants. Notably, the MPP‐based **LAMI** (i.e., **LAMI4**) provided the greatest functional enhancement, showing better performance than isolations based on TPP^+^ ligands across different surface densities (i.e.**, LAMI1**–**LAMI3**). This finding suggests that the superior outcomes of **LAMI4** are not solely dictated by ligand density, but may also reflect intrinsic ligand properties, such as distinct targeting behavior or more favorable interactions with the mitochondrial surface [33, 34, 35]. To further investigate this, we evaluated capture efficiency after carbonyl cyanide 3‐chlorophenylhydrazone (CCCP)‐induced mitochondrial depolarization (Figure S15). The marked decrease in capture efficiency for both **LAMI2** and **LAMI4** suggests that mitochondrial polarization contributes to the capture process. Moreover, **LAMI4** retained higher capture efficiency than **LAMI2** after CCCP treatment, indicating that the MPP motif may afford additional interactions with mitochondria. Collectively, these results demonstrate that the **LAMI** platform enables isolation of mitochondria with enhanced functional preservation, with performance further optimized through rational selection of targeting ligands.

Mitochondrial morphology is closely linked to functional state [36]. Given the outstanding performance of **LAMI4**, we further compared the ultrastructural features of mitochondria isolated by **LAMI4** and by the DC method using bio‐transmission electron microscopy (bio‐TEM). The bio‐TEM images revealed that ^LAMI4^Mito largely retained rod‐like morphology with abundant cristae and clearly defined inner and outer membranes, indicative of well‐preserved ultrastructural integrity. In contrast, ^DC^Mito displayed pronounced structural disruption, characterized by swollen and sparsely organized cristae, consistent with their reduced functional activity (Figure 3k). These results demonstrate that the **LAMI** platform, particularly **LAMI4**, preserves mitochondrial structure substantially better than conventional DC method. Owing to its consistent outperformance, ^LAMI4^Mito was used in all subsequent experiments and is hereafter referred to as ^LAMI^Mito.

### 2.4 Evaluation of LAMI‐Isolated Mitochondria for Therapy in Hepatic IRI

**FIGURE 4:**
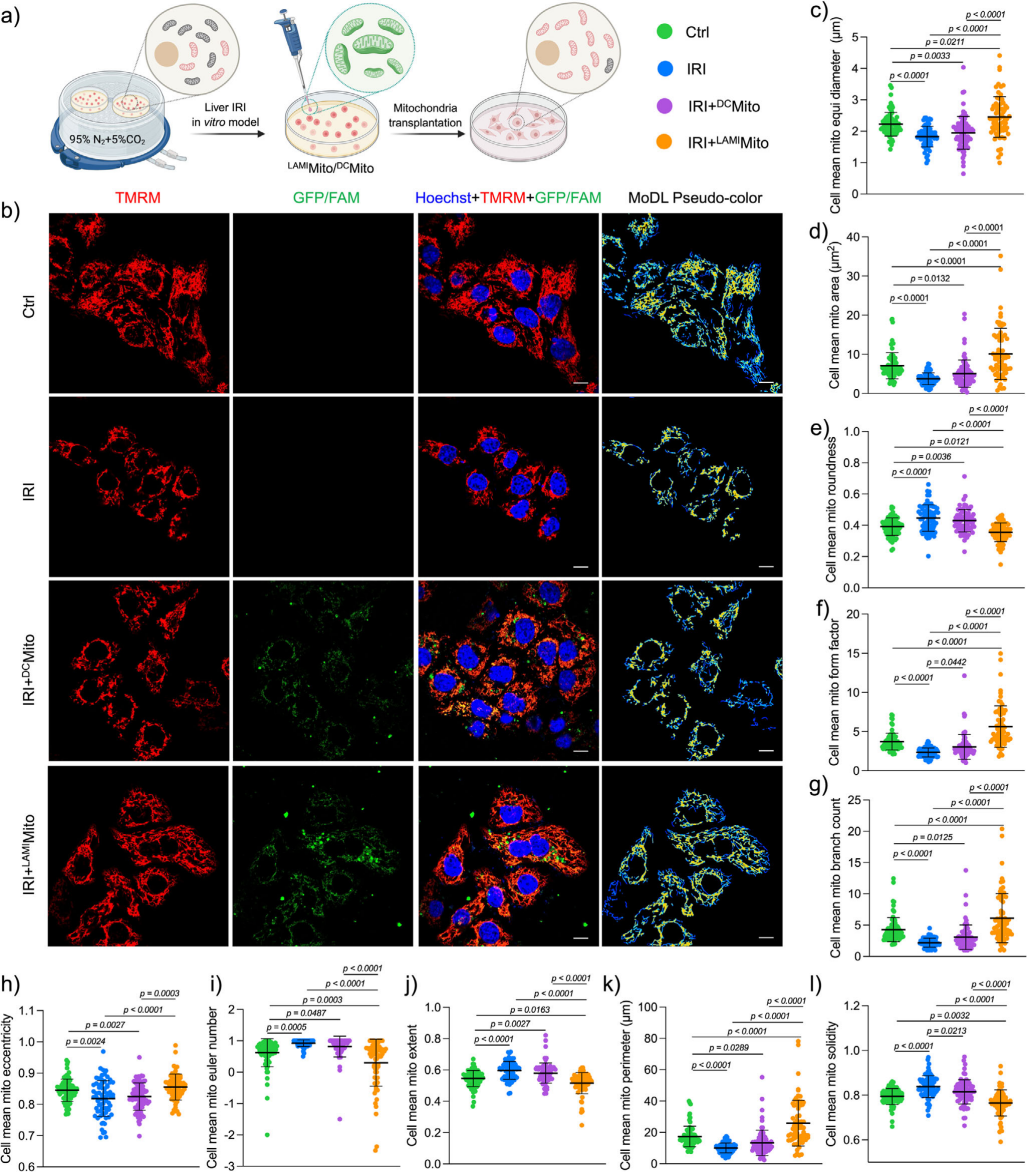
Internalization and morphological analysis of transplanted mitochondria in an in vitro hepatic ischemia‐reperfusion injury (IRI) model. (a) Schematic illustration of the construction of the in vitro hepatic IRI model and subsequent AMT. Four groups were established: control (Ctrl), hypoxia‐reoxygenation (IRI), hypoxia‐reoxygenation cells transplanted with mitochondria isolated by the DC method (IRI+^DC^Mito), and hypoxia‐reoxygenation cells transplanted with mitochondria isolated by the **LAMI**‐based method (IRI+^LAMI^Mito). (b) Confocal fluorescence imaging of hypoxic cells at 12 h post AMT. Mitochondrial morphology was analyzed using the MoDL deep learning algorithm, and the TMRM fluorescence channel was pseudo‐colored accordingly. The pseudo‐color gradient represents the relative intensity of the TMRM fluorescence signal. Cell nuclei were stained with Hoechst 33342 (blue), mitochondria were labelled with TMRM (red), and exogenous mitochondria were derived from HepG2 GFP cells (for ^DC^Mito) or wild‐type HepG2 cells labelled with the FAM fluorophore on **ZT4** molecules (for ^LAMI^Mito). Scale bar = 10 µm. (c–l) Quantitative analysis of mitochondrial morphological parameters extracted by the MoDL model, including equivalent diameter (c), area (d), roundness (e), form factor (f), branch count (g), eccentricity (h), euler number (i), elongation (j), perimeter (k), and solidity (l). Data are presented as mean ± SD (*n* = 80). The *p* values were determined by one‐way ANOVA followed by Tukey's multiple comparison test.

Encouraged by the above data, we next assessed the functional activity of ^LAMI^Mito and ^DC^Mito by performing AMT in an in vitro IRI model (Figure 4a). For mitochondrial donor, here we used HepG2 cells stably expressing mitochondrial targeted green fluorescent protein (HepG2‐Mito‐GFP) for ^DC^Mito, and wild‐type HepG2 cells for ^LAMI^Mito (detected *via* the 5‐FAM signal from the LAMI isolation process). This genetic labeling approach was chosen over conventional chemical dyes, which are prone to leakage and false‐positive signals [37, 38]. At 12 h post‐transplantation, both endogenous and exogenous mitochondria were imaged by confocal microscopy, and the mitochondrial network was subsequently segmented by our in‐house deep learning‐based MoDL algorithm [39, 40]. Compared with the control group (Ctrl), mitochondria in IRI cells exhibited severe fragmentation and shortening, forming clustered structures predominantly localized around the nucleus, confirming successful establishment of the IRI model (Figures 4b and S16). Quantitative MoDL analysis revealed that, relative to the IRI and IRI+^DC^Mito groups, the IRI+^LAMI^Mito group exhibited significant increases in equivalent diameter and mitochondrial area, indicating restoration of elongated and intact tubular structures (Figure 4c–l). Increased shape factor and branch number reflected enhanced mitochondrial networking and interconnectivity, while elevated eccentricity and perimeter indicated improved mitochondrial extension and stretching capacity. In contrast, parameters associated with pathological fragmentation and clustering, including circularity, Euler number, elongation, and solidity, were markedly reduced. Collectively, these multidimensional morphological metrics demonstrate that ^LAMI^Mito‐based AMT promotes robust reorganization of mitochondrial networks, providing a structural basis for subsequent functional recovery.

**FIGURE 5:**
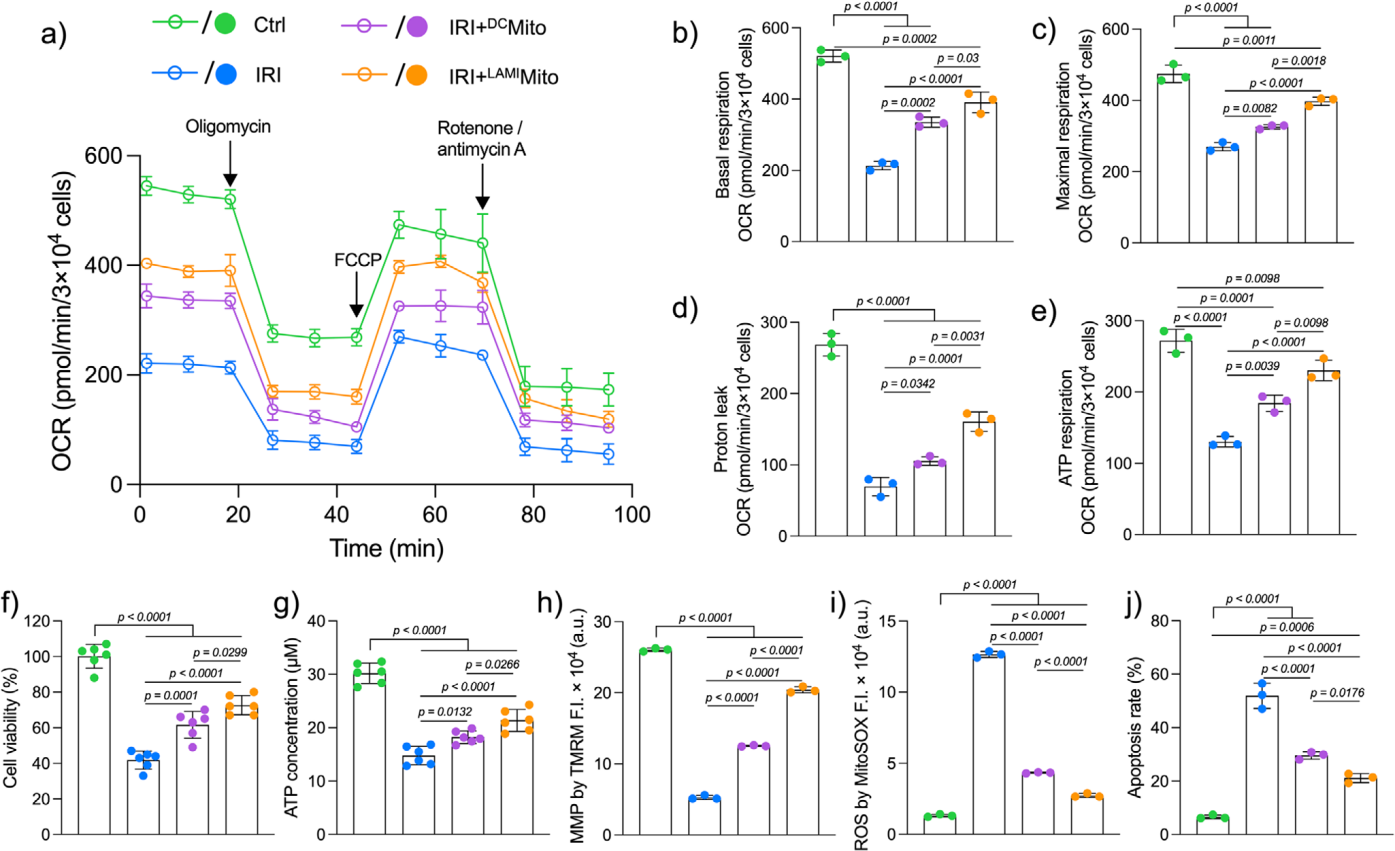
Transplantation of mitochondria isolated by the **LAMI**‐based method ameliorates cellular dysfunction in an in vitro IRI model. (a) Real‐time oxygen consumption rate (OCR) profiles of cells receiving different mitochondrial transplants, measured using a Seahorse XF Analyzer. Arrows indicate sequential injections of oligomycin, FCCP, and rotenone/antimycin A. (b–e) Quantitative analysis of key mitochondrial respiratory parameters derived from the OCR profiles: basal respiration (b), maximal respiration (c), proton leak (d), and ATP respiration (e). For OCR (a–e), *n* = 3 independent biological replicates. (f–j) Functional evaluation of AMT, including cell viability (f) (*n* = 6 independent biological replicates), intracellular ATP content (g) (*n* = 6 independent biological replicates), MMP (assessed by TMRM fluorescence) (h) (*n* = 3 independent biological replicates), ROS levels (i) (*n* = 3 independent biological replicates), and apoptosis rate (j) (*n* = 3 independent biological replicates). Data are presented as mean ± SD, and *p* values were determined by one‐way ANOVA followed by Tukey's multiple comparison test.

At the functional level, the superiority of **LAMI** became further evident. Seahorse XF analysis revealed markedly enhanced respiratory performance of IRI+^LAMI^Mito. As shown in Figure 5a, cells supplemented with ^LAMI^Mito displayed a significantly elevated oxygen consumption rate (OCR) profile compared with those receiving ^DC^Mito. Quantitative assessment confirmed that critical parameters of mitochondrial respiration, including basal respiration, maximal respiration, proton leak, and ATP respiration were all markedly higher in the IRI+^LAMI^Mito group (Figure 5b–e). This enhanced respiratory competence was well reflected in the subsequent cellular functional outcomes. Both ^DC^Mito and ^LAMI^Mito significantly improved cell viability, ATP generation, and mitochondrial membrane potential compared with the IRI group (Figures 5f–h and S17). However, the recovery achieved by ^LAMI^Mito was significantly greater, as reflected by higher intracellular ATP levels and elevated MMP, indicating enhanced bioenergetic capacity and better preservation of mitochondrial integrity.

Similarly, although transplantation with mitochondria isolated by both methods mitigated hypoxia‐induced ROS overproduction and apoptosis, the ^LAMI^Mito displayed a more pronounced suppression of ROS accumulation and a lower apoptotic rate (Figures 5i,j, S18, and S19). These results collectively demonstrate that while ^DC^Mito retained partial functionality, their reparative efficacy remains limited. In contrast, ^LAMI^Mito exhibited superior quality (purity and activity), conferring stronger cytoprotective effects under hypoxic stress and effectively restoring mitochondrial dynamics, energy metabolism, and redox homeostasis. Notably, although the initial internalization of ^DC^Mito and ^LAMI^Mito was visualized using different tracking labels, the subsequent comparative analyses were based on common fluorescence readouts or label‐independent functional assays. Therefore, the observed differences in therapeutic efficacy do not arise from the initial labeling strategy.

## 3 Conclusion

In summary, we have successfully developed the **LAMI** platform by rationally designing a ligand‐programmable, trivalent branched architecture. This modular integration of magnetic capture, customizable targeting probes, and photo‐responsive release enables the high‐efficiency, selective, and non‐destructive extraction of functional mitochondria. Compared with conventional DC method, **LAMI** achieved markedly enhanced mitochondrial quality. Importantly, the photo‐cleavable linker allowed rapid and gentle detachment of mitochondria from the MBs under mild conditions, eliminating the need for chemical triggers and preserving mitochondrial bioactivity. Comprehensive characterizations confirmed that LAMI‐isolated mitochondria retain intact ultrastructure, high MMP, and robust metabolic activity. In a hepatocyte injury model that simulates IRI, the ^LAMI^Mito was efficiently internalized, significantly enhanced intracellular ATP production, and reduced ROS accumulation, thereby promoting functional recovery. Collectively, this work establishes a gentle and efficient platform for high‐quality mitochondrial isolation. The modular and photo‐responsive design of **LAMI** platform overcomes key limitations of existing purification methods, laying a solid foundation for advanced AMT strategies. While the present study demonstrates promising therapeutic efficacy in an in vitro IRI model, subsequent validation in animal models will be essential to fully evaluate its translational potential.

## Author Contributions

**Hui Liu**: conceptualization, methodology, investigation, writing – original draft. **Yuxin Jiao**: investigation. **Ting Zhang**: investigation. **Haiwei Wang**: methodology. **Yufei Xue**: methodology. **Jiayu Ding**: investigation. **Yang Ding**: data curation. **Weisen Zhang**: data curation. **Hua Bai**: supervision. **Bo Peng**: conceptualization, writing – review and editing, supervision, funding acquisition. **Nicolas H. Voelcker**: conceptualization, writing – review and editing, supervision. **Lin Li**: conceptualization, writing – review and editing, funding acquisition.

## Conflicts of Interest

The authors declare no conflicts of interest.

## Supporting information




**Supporting File 1**: anie72910‐sup‐0001‐SuppMat.docx.

## Data Availability

The data that support the findings of this study are available from the corresponding author upon reasonable request.
