# Supplementary material for: Light‐Activated Isolation of High‐Quality Mitochondria for Therapeutic Transplantation
Source: Angew Chem Int Ed Engl. 2026 May 28;65(28):e7935890. doi: 10.1002/anie.7935890 (PMC13340514; doi:10.1002/anie.7935890)
Supplement: Supplementary file 1 — Supporting File 1: anie72910‐sup‐0001‐SuppMat.docx. [file ANIE-65-e7935890-s001.docx]

**Supporting Information**

**Light-activated Isolation of High-quality Mitochondria for Therapeutic Transplantation**

Hui Liu^[a]+^, Yuxin Jiao^[a]+^, Ting Zhang^[a]^, Haiwei Wang^[a]^, Yufei Xue^[a]^, Jiayu Ding^[a]^, Yang Ding^[a,b]^, Meiling Wang^[c]^, Weisen Zhang^[a]^, Hua Bai^[a]^, Bo Peng^[a]^*, Nicolas H. Voelcker^[b]^*, Lin Li^[a,c]^*

^[a]^ State Key Laboratory of Flexible Electronics (LOFE) & Institute of Flexible Electronics (IFE), Northwestern Polytechnical University, Xi'an, 710072, China

^[b]^ Drug Delivery, Disposition and Dynamics, Monash Institute of Pharmaceutical Sciences, Monash University, Parkville, Victoria 3052, Australia

^[c]^ State Key Laboratory of Flexible Electronics (LoFE) & Institute of Flexible Electronics (IFE), Xiamen University, Xiamen 361102, China

[**^*^**] [iambpeng@nwpu.edu.cn](mailto:iambpeng@nwpu.edu.cn) (B.P.); [nicolas.voelcker@monash.edu](mailto:nicolas.voelcker@monash.edu) (N.H.V.); [iamlli@nwpu.edu.cn](mailto:iamlli@nwpu.edu.cn) (L.L.)

[**^+^**] These authors contributed equally to this work

**Table of Contents**

1. Materials and methods...........................................................................................................2
2. Synthesis and characterizations of mitochondrial extraction probes...................................12
3. Supplementary tables and figures........................................................................................21
4. ^1^HNMR and ^13^C NMR spectra of all compounds................................................................34
5. Reference..............................................................................................................................45

**1. Materials and methods**

1.1 General Information

All chemicals were purchased from Sigma-Aldrich or Thermo Fisher Scientific and used without further purification unless otherwise noted. Solvents, including ethanol (EtOH), dichloromethane (DCM), methanol (MeOH), petroleum ether (PE) and ethyl acetate (EA), were obtained from Titan Scientific. Extra dry solvents Acetonitrile (MeCN), [N,N-Dimethylformamide](https://www.medchemexpress.cn/n-n-dimethylformamide.html) (DMF), Dimethyl sulfoxide (DMSO) were purchased from Adamas-beta® (Titan Scientific). All aqueous solutions were prepared using double-distilled water (H_2_O) obtained from a Milli-Q water purification system (Millipore, Billerica, MA, USA) with a resistivity of 18.2 MΩ·cm at 25^o^C, unless otherwise stated. CHA-dde was purchased from Nanjing Peptide Biotech Ltd. (Nanjing, China). MB-NH_2_ was purchased from BioMag^TM^ (Jiangshu, China). The Mitochondria Isolation Kit for DC method was purchased from Thermo Fisher Scientific (Waltham, MA, USA, Cat. No. 89874). The BCA Protein Assay Kit was purchased from Pierce (Rockford, IL, USA). Hoechst 33342 was purchased from Thermo Fisher Scientific (Cat. No. 62249). Tetramethylrhodamine methyl ester (TMRM) was purchased from Thermo Fisher Scientific (Cat. No. I34361).

1.2 Characterization

Reaction progress was monitored by TLC on pre-coated silica plates (250 μm thickness) and spots were visualized by UV light. Column chromatography was carried out using silica gel (Merck 60 F254 nm 0.040–0.063 μm). Liquid chromatography-Mass spectrometry (LC-MS) was performed using an Agilent UHPLC/MS 1260/6120 with a 1260 Infinity G1312B Binary pump and a 1260 Infinity G1367E 1260 HiP ALS autosampler. The detector used is a 1290 Infinity G4212A 1290 DAD, which monitors at 254 nm. High performance liquid chromatography (HPLC) was completed using reverse phase HPLC analysis with a Poroshell 120 EC-C18 column. High-resolution mass spectrometry (HRMS) was tested by an Agilent 6230 Series Accurate Mass TOF using ESI. All ^1^H NMR and ^13^C NMR spectra were taken on a Avance III Nanobay 500 MHz Bruker spectrometer, using DMSO-*d_6_* as the solvent. Chemical shifts are reported in parts per million referenced with respect to residual solvent (CDCl_3_ = 7.26 ppm, DMSO-*d_6_* = 2.50 ppm, CD_3_OD = 3.31 ppm) for ^1^H NMR, (CDCl_3_ = 77.16 ppm, DMSO-*d_6_* = 39.52 ppm, CD_3_OD = 49.03 ppm) for ^13^C NMR. The hydrodynamic diameter and zeta potential (ξ) of the nanoparticles were determined using a Zetasizer Nano ZS instrument (Malvern Panalytical, UK). Scanning electron microscopy (SEM) images were acquired using a ZEISS GeminiSEM 300 microscope (Carl Zeiss, Germany) at an accelerating voltage of 3 kV. Transmission electron microscopy (TEM) was performed on a Hitachi HT7700 microscope (Hitachi, Japan). Flow cytometry analysis was conducted on a CytoFLEX S flow cytometer (Beckman Coulter, USA). Fourier-transform infrared (FTIR) spectra were recorded on a Bruker Tensor II spectrometer (Bruker, Germany). UV–Vis absorption spectroscopy (Hitachi U-3900H) was used to monitor the decomposition of the probes. Upon irradiation with 395 nm UV light (5 mW/cm^2^), all probes decomposed within 2 min (0.6 J/cm^2^). Fluorescence spectra were measured with a HITACHI F-7100 fluorescence spectrophotometer (Hitachi, Japan). Confocal laser scanning microscopy (CLSM) images were captured using a Nikon C2 system, and super-resolution imaging was performed on a Nikon N-SIM E system (Nikon, Japan). The cellular oxygen consumption rate (OCR) was measured using an Agilent Seahorse XFe24 Analyzer (Agilent Technologies, USA).

1.3 Cell Culture

The HepG2 cell line, derived from human hepatocellular carcinoma, was purchased from the American Type Culture Collection (ATCC) and exhibits an epithelial morphology. Green fluorescent protein (GFP) was introduced into HepG2 cells *via* viral transduction, using lentiviral vectors provided by Xi’an Tiancheng Biotechnology Co., Ltd. These genes allow mitochondria to specifically express GFP, enabling mitochondrial targeting and visualization under fluorescence microscopy. Upon excitation at specific wavelengths, mitochondria emit green fluorescence in the cells. Cells were cultured in Dulbecco’s Modified Eagle Medium (DMEM) supplemented with 10% heat-inactivated fetal bovine serum (FBS, Gibco) and 1% penicillin-streptomycin (P/S). Cultures were maintained in a humidified incubator at 37^o^C with 5% CO_2_ and 95% air. Cells were sub-cultured regularly to maintain exponential growth. Cell density was measured using a hemocytometer. All procedures were performed in a biosafety cabinet under sterile conditions prior to experimental use.

1.4 Isolation Buffer for Mitochondria

To prepare mitochondrial isolation buffer (100 mL), accurately weigh sucrose (8.5573 g), KCl (74.55 mg), MgCl_2_ (14.28 mg), EDTA (29.22 mg), EGTA (38.04 mg), and HEPES (476.60 mg). Add the components to double-distilled water (95 mL), insert a magnetic stir bar, and stir thoroughly on a magnetic stirrer. Adjust the pH to 7.5 using 1 M KOH. Store the isolation buffer at 4^o^C.^[1,2]^

1.5 Respiration Buffer for Mitochondria

To prepare mitochondrial respiration buffer (100 mL), accurately weigh sucrose (8.5573 g), KH_2_PO_4_ (27.22 mg), MgCl_2_ (95.21 mg), HEPES (476.60 mg), and EGTA (19.02 mg). Add the components to double-distilled water (100 mL), insert a magnetic stir bar in the solution and mix thoroughly using a magnetic stirrer. Store the respiration buffer at 4^o^C.^[3]^

1.6 Mitochondrial Isolation Protocol

For the differential centrifugation (DC) method, mitochondria were isolated from HepG2 cells using the Mitochondria Isolation Kit (Thermo Scientific, Cat. No. 89874) for Cultured Cells following option B of the manufacturer’s protocol to obtain purified mitochondria (denoted as ^DC^Mito). For the LAMI-based isolation, mitochondria with high purity were isolated from wild-type HepG2 cells using **LAMI** platform. Briefly, 2 × 10⁷ HepG2 cells were harvested by trypsinization, washed with PBS, and centrifuged at 850× g for 5 min at 4^o^C. The cell pellet was resuspended in isolation buffer supplemented with 1% DTT and a protease inhibitor cocktail, followed by gentle homogenization on ice (approximately 30–60 strokes) using a pre-chilled glass homogenizer to achieve adequate disruption while maintaining mitochondrial integrity. Next, 800 μL of the cell lysate was mixed with an equal volume of respiration buffer, followed by the addition of pretreated **LAMI** beads. The mixture was incubated on a rotary shaker at 4^o^C for 1 h in the dark to promote the specific binding of mitochondria to the magnetic beads (MBs). After incubation, the bead–mitochondria complexes were separated on ice using a magnetic rack for 5 min, and the supernatant was discarded (or retained for Western blot analysis if required). The complexes were then resuspended in 200 μL of respiration buffer and exposed to 395 nm light at a power density of 5 mW cm^–2^ for 2 min (total fluence: 0.6 J cm^–2^) to cleave the photo-cleavable linker, thereby releasing intact mitochondria. Finally, the suspension was magnetically separated again, and the supernatant containing high-purity mitochondria was collected for subsequent experiments.

1.7 Imaging LAMI beads and Mitochondria by SEM

The isolated mitochondria, mitochondria–bead complexes, and bare beads were fixed overnight in electron microscopy fixative at 4^o^C. Samples were then dehydrated through a graded ethanol series and subjected to critical point drying to remove water and prevent structural collapse. The dried samples were gently mounted onto conductive adhesive tape and sputter-coated with gold using a Quorum SC7620 sputter coater at 5 mA for 45 s to ensure adequate surface conductivity. Finally, the prepared samples were examined using a ZEISS GeminiSEM 300 SEM at an accelerating voltage of 3 kV.

1.8 Imaging Mitochondria by TEM

To assess mitochondrial morphology following isolation, mitochondria were fixed overnight in an electron microscopy fixative at 4^o^C. The samples were washed three times with 0.1 M phosphate buffer (15 min per wash). The mitochondria were then subjected to graded dehydration using ethanol and acetone at 4^o^C. Subsequently, the samples were infiltrated with pure embedding resin at 37^o^C for 2–3 h and polymerized overnight at 37^o^C. After polymerization, ultrathin sections were prepared and stained with lead citrate and 50% uranyl acetate–saturated ethanol solution for 5–10 min. Finally, the sections were examined using a TEM (Hitachi HT7700, Japan).

1.9 Quantifying Mitochondrial Protein

Mitochondrial protein concentration was determined using the Pierce BCA Protein Assay Kit (Pierce, Rockford, IL) as a critical reference for subsequent experiments. Mitochondrial lysates were prepared by mixing mitochondrial suspension (10 μL) with RIPA lysis buffer (40 μL), followed by incubation on ice for 30 min. A standard curve (0, 5, 25, 50, 125, and 250 μg mL^–1^) was prepared using PBS. Both standards and lysates were loaded (10 μL per well, in triplicate) into a 96-well plate. A BCA working reagent (1.6 mL) was prepared by mixing Reagent A and Reagent B at a 50:1 ratio, and working reagent (90 μL) was added to each well. After incubation at 37^o^C for 30 min, absorbance was measured at 562 nm using a microplate reader.

1.10 Western Blot (WB) Assay

Mitochondrial lysates were prepared by incubating isolated mitochondria in RIPA buffer supplemented with protease and phosphatase inhibitors (Shanghai, China) for 30 min, with brief vortexing every 10 min. Following centrifuged at 12,000× g for 15 min at 4^o^C, the supernatants were collected. Protein concentrations were determined using a BCA Assay. Equal amounts of total protein were separated by 10% SDS-PAGE and transferred onto Immobilon-FL PVDF membranes (Millipore, Billerica, MA, USA). After blocking for 1 h at room temperature with Odyssey blocking buffer (Li-Cor Biosciences, Lincoln, NE, USA), membranes were sectioned according to target molecular weights and incubated overnight at 4^o^C with primary antibodies against *β*-Actin (Bioworld, AP0060), COX IV (Abcam, ab16056), and VDAC1 (Abcam, ab14734). Following three washes with TBST (Tris-buffered saline with 0.1% Tween-20). Following three washes with TBST, membranes were incubated for 1 h at room temperature with corresponding secondary antibodies: Anti-rabbit IgG (H&L) DyLight 800-4xPEG Conjugate (Cell Signaling Technology, #5151) and Anti-mouse IgG (H&L) DyLight 680 Conjugate (Cell Signaling Technology, #5470). After final washes, protein bands were visualized and analyzed using an Odyssey CLX dual-color infrared laser imaging system.

1.11 Flow Cytometry Analysis

Flow cytometry was performed to quantify mitochondrial particles and evaluate isolation purity. The gating strategy was established based on a fluorescence-validated particle reference. Specifically, mitochondrial particles isolated by the **LAMI4** platform (^LAMI4^Mito) were used to define the optimal gate, as these particles were both labeled with FAM and independently verified for high purity *via* Western Blot (enrichment of VDAC1 and COX IV). By identifying the particles with high green fluorescence intensity in the ^LAMI4^Mito sample (isolated from wild-type HepG2 cells), we defined a specific gate on the FSC vs. SSC plot that corresponds to intact, pure mitochondria. This predefined gate was then applied to analyze mitochondria samples isolated by the DC method and other **LAMI** variants under identical instrument settings (100 μL fixed detection volume, medium flow rate). Isolation efficiency was evaluated by comparing particle counts within this validated gate^[4]^.

1.12 Hypoxia–Reoxygenation Cell Model Construction

Wild-type HepG2 cells were seeded in confocal culture dishes and incubated overnight at 37^o^C in a humidified atmosphere containing 5% CO_2_. Upon reaching 60–70% confluence, the culture medium was removed, and the cells were gently rinsed with PBS before replacement with Krebs–Ringer buffer (KRB). The dishes were transferred into a hypoxia chamber and purged with a gas mixture of 95% N_2_ and 5% CO_2_ to displace ambient air and establish hypoxic conditions. The sealed chamber was then incubated at 37^o^C for 6 h to induce hypoxia. Following hypoxic exposure, KRB buffer was replaced with fresh complete medium. Cells were washed twice with PBS and subsequently maintained under normoxic conditions (37°C, 5% CO_2_) for reoxygenation.

1.13 Mitochondrial Transplantation for Attenuating Hypoxia-Reoxygenation Injury

Cells were assigned to four groups: Ctrl (untreated control), IRI (hypoxia-reoxygenation), IRI+^DC^Mito (IRI with mitochondria isolated by DC method), and IRI+^LAMI^Mito (IRI with mitochondria isolated *via* the **LAMI** platform–**LAMI4**). Wild-type HepG2 recipient cells were seeded in confocal dishes. Upon reaching 60–70% confluence, hypoxia-reoxygenation treatment was performed as described above. Mitochondria were isolated from HepG2-GFP cells by the DC method or from wild-type HepG2 cells using **LAMI**-based method. The resulting mitochondrial pellets were resuspended in respiration buffer and gently homogenized. Mitochondria were added to hypoxic cells at a dose of 5 µg mitochondrial protein per 10⁴ cells and incubated at 37°C in 5% CO_2_ for 12 h to permit cellular internalization. After incubation, cells were washed twice with PBS and replaced with phenol red-free medium (1 mL). Mitochondrial uptake was then visualized using laser scanning confocal microscopy.

1.14 Confocal Imaging of Cells Following Hypoxic–Reoxygenation

Mitochondrial morphology following hypoxia–reoxygenation was evaluated by CLSM. HepG2 cells were seeded in confocal plates at 8 × 10^4^ cells per well and treated as described in Section 1.13. After hypoxia-reoxygenation and subsequent mitochondrial transplantation (12 h), nuclei were stained with Hoechst 33342 (ThermoFisher, Cat. No. 62249), and mitochondria were labeled with tetramethylrhodamine methyl ester (TMRM). Exogenous mitochondria were derived from HepG2 GFP cells (for ^DC^Mito) or wild-type HepG2 cells labelled with the FAM fluorophore on **ZT4** molecules (for ^LAMI^Mito). Fluorescence images were acquired using excitation wavelengths of 405, 488, and 561 nm with a 100× oil-immersion objective. Image analysis was performed using Image J software.

1.15 Measurement of Oxygen Consumption Rate after Hypoxia-reoxygenation Treatment

The oxygen consumption rate (OCR) was determined using an Agilent Seahorse XFe24 Cellular Energy Metabolism Analyzer. HepG2 cells were seeded in Seahorse XF cell culture plates at a density of 3 × 10^4^ cells per well. Cells were divided into four groups: Ctrl, IRI, IRI+^DC^Mito, and IRI+^LAMI^Mito. After overnight attachment, hypoxia–reoxygenation treatment was performed as described above. In parallel, the Seahorse sensor cartridge was hydrated according to the manufacturer’s instructions. Mitochondria were isolated from wild-type HepG2 cells cultured in two T75 flasks. The mitochondrial pellets were resuspended in complete medium (1 mL). After removal of the culture medium from the seahorse plate and washing twice with PBS, Mitochondrial suspension (250 μL) was added to each well. Cells was incubated at 37^o^C in a humidified 5% CO_2_ atmosphere for 12 h to allow mitochondrial internalization. Following incubation, cells were washed twice with Seahorse XF DMEM assay medium and then maintained in fresh XF DMEM (500 μL) per well. The plate was equilibrated at 37°C in a non-CO_2_ incubator for 60 min prior to measurement. For mitochondrial stress testing, oligomycin (1 µM), FCCP (0.5 µM), and rotenone/antimycin A (0.5 µM) were sequentially injected according to the manufacturer’s protocol. Basal respiration, ATP-linked respiration, proton leak, and maximal respiration were calculated from real-time OCR measurements. For Seahorse XF analysis, n = 3 denotes three independent biological replicates. Because the Ctrl group and the hypoxia-reoxygenation groups (IRI, IRI+^DC^Mito, and IRI+^LAMI^Mito) required different environmental conditioning prior to measurement, the assays were performed using two separate Seahorse XFe24 plates. Plate 1 was used for the Ctrl group, whereas Plate 2 was used for the IRI, IRI+^DC^Mito, and IRI+^LAMI^Mito groups. Statistical analysis was performed on the three independent biological replicates.

1.16 Cell Viability Assay

Cell viability was assessed using the Cell Counting Kit-8 (CCK-8) assay. HepG2 cells were seeded in 96-well plates at a density of 8 × 10^3^ cells per well and cultured until approximately 80% confluence. Cells were assigned to four groups: Ctrl, IRI, IRI+^DC^Mito, and IRI+^LAMI^Mito. Hypoxia–reoxygenation treatment and mitochondrial transplantation were performed as described above. After 12 h of incubation (37^o^C, 5% CO_2_), the culture medium was replaced with CCK-8 working solution (CCK-8: DMEM = 1: 9, v/v), and cells were incubated for an additional 1 h at 37^o^C. Absorbance was measured at 570 nm (test wavelength) and 600 nm (reference wavelength) using a microplate reader.

1.17 ATP Levels Analysis

ATP levels in mitochondria isolated by different methods were measured to evaluate mitochondrial activity. For each group, mitochondrial protein (10 µg, quantified by BCA assay) from each of the three independent biological replicates was transferred into separate wells of a white 384-well plate. An equal volume of CellTiter-Glo® Luminescent Assay reagent (Promega, 25 µL) was added to each well, followed by orbital shaking for 2 min to induce lysis. After incubation at room temperature for 10 min, luminescence was measured using a multimode microplate reader (Spark, Tecan).

Similarly, cellular ATP levels were quantified in HepG2 cells (seeded in 12-well plates at 8 × 10^4^ cells/well) following hypoxia-reoxygenation treatment and mitochondrial transplantation, as described in Section 1.13. Cells were resuspended in PBS (150 µL), and an aliquot (25 μL) was transferred into a 384-well plate. The addition of CellTiter-Glo reagent induced cell lysis (25 μL), and the luminescence signal was measured after a 10 min incubation at room temperature in the dark using a multifunctional plate reader (Spark, Tecan).

1.18 Mitochondrial Membrane Potential Analysis

Mitochondrial membrane potential (MMP) was analyzed using the TMRM by flow cytometry. For isolated mitochondria, protein (100 µg, quantified by BCA assay) was incubated with TMRM (100 nM) in assay buffer for 30 min at 37°C in the dark. Samples were centrifuged to remove excess dye, washed with PBS and resuspended in PBS for immediate analysis. Fluorescence was detected by flow cytometric analysis (Ex/Em = 548/573 nm).

For cellular MMP analysis, cells were incubated with serum-free medium (1 mL) containing TMRM (100 nM) for 30 min at 37°C in the dark following mitochondrial transplantation. Cells were washed twice with PBS, trypsinized, collected, and resuspended in PBS (500 μL) prior to flow cytometric analysis (Ex/Em = 548/573 nm).

1.19 ROS Level Analysis

Mitochondrial superoxide generation was assessed using the MitoSOX™ Red fluorescent probe and analyzed by flow cytometry. For isolated mitochondria, protein (100 µg, quantified by BCA assay) was incubated with MitoSOX Red (5 μM) in assay buffer for 30 min at 37°C in the dark. The mitochondria were then centrifuged, washed with PBS, and resuspended in PBS before flow cytometric analysis (Ex/Em = 510/580 nm).

For cellular ROS measurement, cells were incubated with serum-free working solution (1 mL) containing MitoSOX Red (5 μM) for 30 min at 37°C in the dark following mitochondrial transplantation. After washing with PBS, collected, resuspended in PBS (500 μL), and subjected to flow cytometric analysis (Ex/Em = 510/580 nm).

1.20 Apoptosis Assay

Apoptosis was evaluated using an Annexin V-FITC/PI Apoptosis Detection Kit (EnoGene, BA1150). HepG2 cells were seeded in 6-well plates at a density of 2 × 10^5^ cells per well and assigned to experimental groups as described above. After mitochondrial transplantation, cells were harvested, washed twice with ice-cold PBS, and resuspended in binding buffer (100 μL). Then Annexin V-FITC (2.5 μL) and propidium iodide (PI, 2.5 μL) were added and incubated for 20 min at room temperature in the dark. After adding the binding buffer (400 μL), dual-channel fluorescence was detected using a flow cytometer (FITC: Ex/Em = 488/525 nm; PI: Ex/Em = 535/617 nm). Unstained, single-stained (Annexin V-FITC or PI), and fully stained controls were used for fluorescence compensation and gating calibration.

**2. Synthesis and characterization of mitochondrial isolation probes**

**
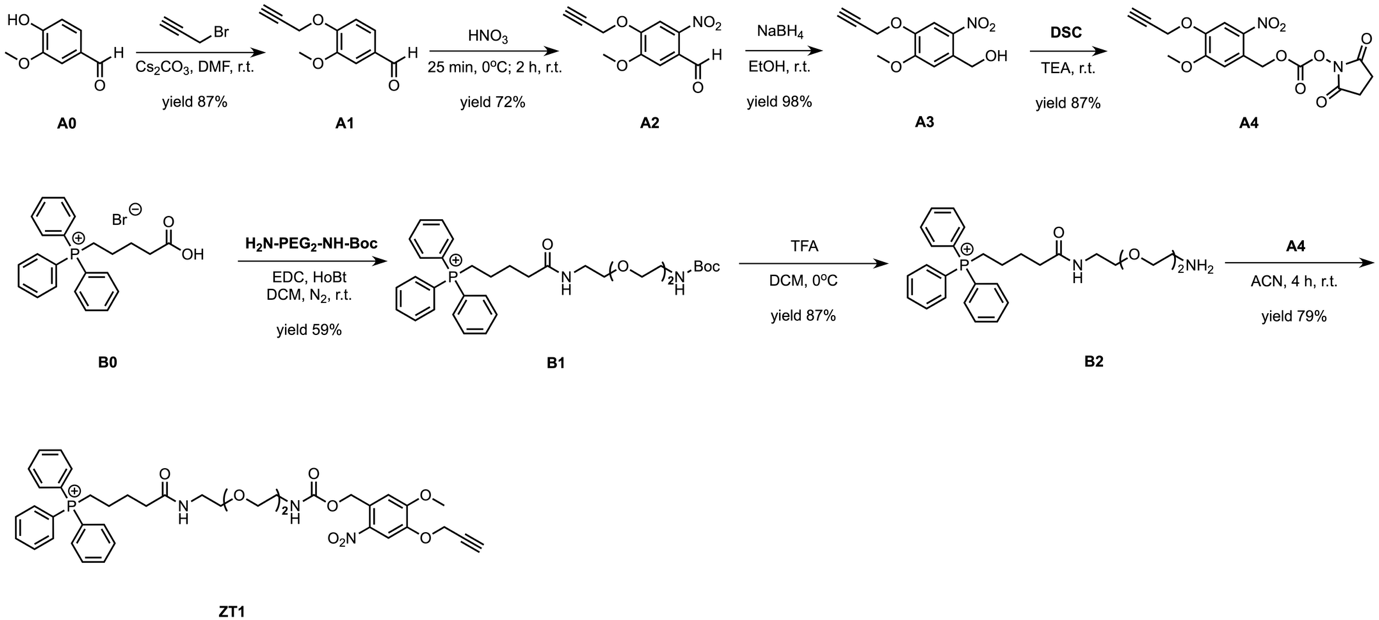
**

**Scheme S1**. Synthetic route for light-controlled mitochondrial isolation probes, including intermediates **A1**–**A4**, **B0**–**B2** and **ZT1**.


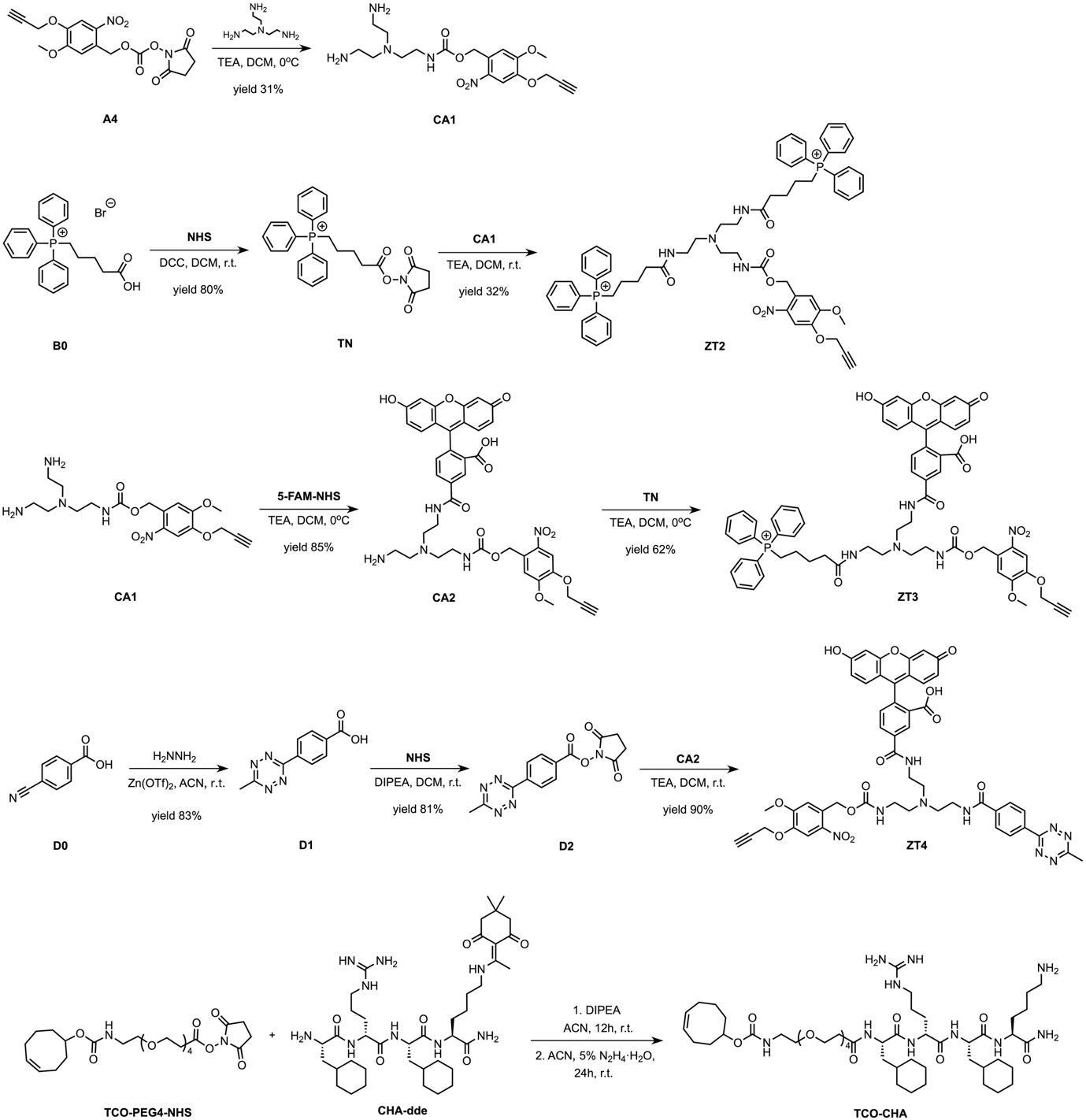


**Scheme S2**. Synthetic route for light-controlled and multifunctional mitochondrial isolation probes, including trivalent **ZT2**–**ZT4** probes and **TCO-CHA**.

Synthesis of **A1**: Vanillin (A0, 1.50 g, 9.9 mmol) and cesium carbonate (3.3 g, 9.9 mmol) were added to a round-bottom flask (100 mL) containing anhydrous N, N-dimethylformamide (20 mL). The mixture was stirred at room temperature (5 min) to ensure complete dissolution, followed by the addition of 3-bromo-1-propyne (3.74 mL, 22.8 mmol). The reaction mixture was stirred at room temperature for 24 h. After completion, the reaction was quenched with water and extracted with ethyl acetate (3 × 30 mL) and brine. The organic layers were combined, dried, and concentrated under reduced pressure. The crude product was purified by column chromatography using ethyl acetate/petroleum ether (1: 2, v/v) as the eluent to afford a white solid **A1** (1.65 g, 87%).^[5,6]^ ^1^H NMR (500 MHz, CDCl_3_) δ 9.85 (s, 1H), 7.45 (dd, *J* = 8.1, 1.9 Hz, 1H), 7.40 (d, *J* = 1.9 Hz, 1H), 7.13 (d, *J* = 8.3 Hz, 1H), 4.85 (d, *J* = 2.5 Hz, 2H), 3.91 (s, 3H), 2.66 (t, *J* = 2.4 Hz, 1H).

Synthesis of **A2**: Compound **A1** (1.0 g, 5.5 mmol) was dissolved in concentrated nitric acid (40 mL) in a round-bottom flask (100 mL) under an ice bath and protected from light. The mixture was stirred for 25 min to ensure complete mixing and then allowed to react at room temperature for 2 h. After completion, the reaction was quenched by the addition of cold water. The mixture was allowed to stand until a large amount of precipitate formed and the supernatant became nearly colourless and transparent. The resulting yellowish solid was collected by vacuum filtration and washed thoroughly with ice-cold water to afford compound **A2** (887 mg, 72%) as a pale-yellow solid.^[7]^ ^1^H NMR (500 MHz, CDCl_3_) δ 10.47 (s, 1H), 7.80 (s, 1H), 7.44 (s, 1H), 4.92 (d, *J* = 2.4 Hz, 2H), 4.03 (s, 3H), 2.63 (t, *J* = 2.4 Hz, 1H).

Synthesis of **A3**: Compound **A2** (510 mg, 2.17 mmol) was dissolved in ethanol (40 mL) under light-protected conditions. Separately, sodium borohydride (250 mg, 6.6 mmol) was dissolved in 1 M sodium hydroxide solution (35 mL). The two solutions were mixed and stirred at room temperature for 2.5 h. The completion of the reaction was confirmed by thin-layer chromatography (TLC). The reaction mixture was then neutralized with dilute hydrochloric acid and extracted with ethyl acetate and saturated sodium chloride solution. The organic layer was dried and evaporated under reduced pressure to give compound **A3** (505 mg, 98%) as a yellow solid.^[8]^ ^1^H NMR (500 MHz, CDCl_3_) δ 7.89 (s, 1H), 7.22 (s, 1H), 4.98 (d, *J* = 6.4 Hz, 2H), 4.84 (d, *J* = 2.4 Hz, 2H), 4.01 (s, 3H), 2.58 (t, *J* = 2.4 Hz, 1H).

Synthesis of **A4**: Compound **A3** (100 mg, 0.422 mmol) was dissolved in acetonitrile (5 mL) under light-protected conditions. N, N'-Disuccinimidyl carbonate (DSC, 324 mg, 1.266 mmol) and triethylamine (185 µL) were added, and the mixture was stirred at room temperature for 2 h. After the reaction was complete, the mixture was extracted with ethyl acetate and saturated ammonium chloride solution. The organic layer was collected and evaporated under reduced pressure to afford a crude product, which was purified by column chromatography (ethyl acetate: petroleum ether = 1: 2, v/v) to yield compound **A4** (139 mg, 87%) as a white solid.^[8]^ ^1^H NMR (500 MHz, CDCl_3_) δ 7.95 (s, 1H), 7.08 (s, 1H), 5.80 (s, 2H), 4.85 (d, *J* = 2.3 Hz, 2H), 4.06 (s, 3H), 2.86 (s, 4H), 2.59 (t, *J* = 2.4 Hz, 1H).

Synthesis of **B1**: Compound **B0** (255 mg, 0.58 mmol) was dissolved in anhydrous dichloromethane (20 mL) and cooled in an ice bath under a nitrogen atmosphere. To the solution, 1-ethyl-3-(3-dimethylaminopropyl)carbodiimide hydrochloride (EDC, 122 mg, 0.64 mmol) and 1-hydroxybenzotriazole (HOBt, 86 mg, 0.64 mmol) were added and stirred for 15 min. Then, tert-butyl [2-(2-(2-aminoethoxy)ethoxy)ethyl]carbamate (143 mg, 0.58 mmol) and N-methylmorpholine (117 mg, 1.16 mmol) were added to the mixture and stirred at room temperature for 6 h. Upon completion, the reaction mixture was extracted with dichloromethane and washed successively with saturated sodium bicarbonate and saturated sodium chloride solutions. The organic phase was dried and concentrated under reduced pressure to yield a transparent oily product **B1** (200 mg, 59%). ^1^H NMR (500 MHz, CDCl_3_) δ 8.75 (s, 1H), 7.88 – 7.65 (m, 15H), 5.28 (s, 1H), 3.76 (t, *J* = 15.1 Hz, 2H), 3.61 – 3.54 (m, 6H), 3.50 (t, *J* = 5.2 Hz, 2H), 3.38 (q, *J* = 6.0 Hz, 2H), 3.27 (q, *J* = 5.6 Hz, 2H), 2.64 (t, *J* = 7.0 Hz, 2H), 1.92 (p, *J* = 6.8 Hz, 2H), 1.63 (q, *J* = 6.8 Hz, 2H), 1.41 (d, *J* = 4.6 Hz, 9H).

Synthesis of **B2**: Compound **B1** (200 mg, 0.34 mmol) was dissolved in dichloromethane (3 mL) and cooled to 0^o^C. Trifluoroacetic acid (25 mL, 0.34 mmol) was added dropwise, and the reaction mixture was stirred at room temperature for 2 h. After completion, the mixture was diluted with dichloromethane and concentrated under reduced pressure. This dilution-evaporation process was repeated five times to completely remove residual trifluoroacetic acid, affording compound **B2** as a transparent oily residue. **B2** was used directly in the subsequent reaction without further purification.

Synthesis of **ZT1**: Compound **B2** (83 mg, 0.168 mmol) was dissolved in acetonitrile (5 mL), followed by the addition of triethylamine (200 μL) and compound **A4** (64 mg, 0.169 mmol). The reaction was conducted at room temperature under light-protected conditions for 4 h. After completion, the crude product was purified by column chromatography (dichloromethane: methanol = 20: 1, v/v), yielding **ZT1** as a yellow solid (100 mg, 79%). ^1^H NMR (500 MHz, CDCl_3_) δ 8.30 (s, 1H), 7.85 (s, 1H), 7.81 – 7.64 (m, 15H), 7.15 (s, 1H), 6.52 (s, 1H), 5.44 (s, 2H), 4.80 (d, *J* = 2.4 Hz, 2H), 3.95 (s, 3H), 3.58 (d, *J* = 5.6 Hz, 4H), 3.55 (dd, *J* = 11.2, 5.3 Hz, 4H), 3.38 (p, *J* = 5.3 Hz, 4H), 2.61 (s, 2H), 2.56 (t, *J* = 2.4 Hz, 1H), 2.47 (t, *J* = 7.3 Hz, 2H), 1.89 (p, *J* = 6.9 Hz, 2H), 1.65 (q, *J* = 7.0 Hz, 2H). ^13^C NMR (126 MHz, CDCl_3_) δ 173.36, 156.49, 154.72, 145.29, 138.91, 135.19, 135.17, 133.78, 133.70, 130.99, 130.66, 130.56, 118.75, 118.07, 110.98, 110.24, 70.50, 70.31, 70.27, 69.90, 63.15, 57.19, 57.09, 45.88, 41.17, 34.24, 26.23, 26.10, 21.71, 21.31, 8.75. HRMS (ESI) calcd for [M]^+^: 756.3044 (m/z). Found: 756.3036.

Synthesis of **CA1**: A solution of Compound **A4** (300 mg, 0.79 mmol) in anhydrous dichloromethane (10 mL) was prepared. Separetely, tris(2-aminoethyl)amine (180 mg, 1.23 mmol) was dissolved in anhydrous dichloromethane (10 mL), followed by the addition of triethylamine (330 µL). The mixture was stirred for 5 min at 0^o^C, and the **A4** solution was then added dropwise. The reaction was allowed to proceed at room temperature for 6 h. After completion, the crude product was purified by column chromatography (dichloromethane: methanol = 10:1, v/v) to yield compound **CA1** as a yellow product (102 mg, 31%). ^1^H NMR (500 MHz, CDCl_3_) δ 7.85 (s, 1H), 7.04 (s, 1H), 6.47 (s, 1H), 5.49 (s, 2H), 4.82 (d, *J* = 2.5 Hz, 2H), 3.97 (s, 3H), 3.28 (d, *J* = 5.2 Hz, 2H), 2.76 (t, *J* = 5.9 Hz, 4H), 2.60 (t, *J* = 5.8 Hz, 2H), 2.57 (t, *J* = 2.4 Hz, 1H), 2.55 (t, *J* = 5.9 Hz, 4H).

Synthesis of **TN**: Compound **B0** (514 mg, 1.16 mmol) was dissolved in anhydrous dichloromethane (40 mL). N-Hydroxysuccinimide (161 mg, 1.40 mmol) and N, N'-dicyclohexylcarbodiimide (DCC, 260 mg, 1.26 mmol) were added, and the mixture was stirred at room temperature for 3 h. The reaction mixture was then filtered, and the crude product was purified by column chromatography (dichloromethane: methanol = 20: 1, v/v) to yield compound **TN** as a white solid (416 mg, 80%). ^1^H NMR (500 MHz, CDCl_3_) δ 7.79 – 7.60 (m, 15H), 3.71 (dd, *J* = 16.4, 13.2 Hz, 2H), 2.76 (s, 4H), 2.67 (t, *J* = 6.7 Hz, 2H), 2.07 (p, *J* = 7.0 Hz, 2H), 1.76 (t, *J* = 8.0 Hz, 2H).

Synthesis of **ZT2**: Compound **CA1** (55 mg, 0.13 mmol) was dissolved in anhydrous dichloromethane (15 mL), and triethylamine (108 µL) was added, followed by **TN** (120 mg, 0.26 mmol). The reaction mixture was stirred at room temperature for 4 h. After dilution with dichloromethane, the mixture was washed with saturated sodium bicarbonate solution and evaporated to dryness. The crude product was purified by column chromatography (dichloromethane: methanol = 15: 1, v/v) to afford the yellow solid **ZT2** (46 mg, 32%). ^1^H NMR (500 MHz, CDCl_3_) δ 8.91 (s, 1H), 7.78 (d, *J* = 10.4 Hz, 2H), 7.75 – 7.61 (m, 30H), 7.52 – 7.37 (m, 1H), 7.28 (s, 1H), 5.39 – 5.19 (m, 4H), 4.78 (d, *J* = 20.9 Hz, 3H), 3.99 – 3.88 (m, 4H), 3.37 (s, 2H), 3.12 (s, 6H), 2.57 (s, 1H), 2.50 – 2.45 (m, 5H), 2.43 (s, 4H), 2.17 (t, *J* = 7.7 Hz, 1H), 1.99 (d, *J* = 6.2 Hz, 2H), 1.86 (t, *J* = 7.0 Hz, 4H). ^13^C NMR (126 MHz, CDCl_3_) δ 173.46, 161.10, 154.93, 145.01, 138.38, 135.08, 135.05, 133.65, 133.57, 130.62, 130.52, 130.44, 129.99, 129.98, 118.66, 117.97, 110.79, 110.20, 62.72, 57.11, 55.79, 55.38, 50.42, 37.74, 34.41, 29.86, 29.85, 29.78, 29.71, 29.65, 29.60, 29.59, 29.47, 29.40, 29.35, 27.29, 26.44, 25.64, 22.77, 21.62, 14.23. HRMS (ESI) calcd for [M]^2+^: 550.2400 (m/z), found 550.2420.

Synthesis of **CA2**: Compound **CA1** (130 mg, 0.32 mmol) was dissolved in anhydrous dichloromethane (10 mL), followed by the addition of triethylamine (134 µL). The mixture was stirred at 0^o^C for 5 min. 5-Carboxyfluorescein succinimidyl ester (100 mg, 0.211 mmol) was dissolved in N, N-dimethylformamide (5 mL) and added dropwise to the **CA1** solution. The reaction was stirred at room temperature for 6 h. The mixture was filtered to afford compound **CA2** as an orange solid (137 mg, 85%). ^1^H NMR (500 MHz, DMSO-*d6*) δ 8.82 (s, 1H), 8.44 (s, 1H), 7.92 (s, 1H), 7.81 (s, 1H), 7.61 (s, 1H), 7.20 (s, 2H), 6.58 (s, 2H), 6.21 (s, 4H), 5.32 (s, 2H), 4.93 (s, 2H), 3.86 (s, 3H), 3.64 (s, 2H), 3.34 (s, 2H), 3.11 (s, 2H), 2.58 (d, *J* = 31.2 Hz, 6H), 1.99 (dt, *J* = 12.1, 6.8 Hz, 1H). ^13^C NMR (126 MHz, DMSO-*d6*) δ 155.83, 153.74, 145.21, 138.93, 129.67, 128.95, 110.94, 110.32, 102.66, 79.24, 78.37, 62.32, 56.49, 56.27, 48.61, 35.13, 31.29, 29.09, 29.04, 28.99, 28.88, 28.84, 28.75, 28.70, 28.59, 26.56, 25.12, 22.11, 13.95.

Synthesis of **ZT3**: Compound **CA2** (30 mg, 0.039 mmol) was dissolved in N, N-dimethylformamide (5 mL), followed by the addition of triethylamine (35 µL). Compound **TN** (40 mg, 0.086 mmol) was then added, and the mixture was stirred at room temperature for 2 h. The crude product was purified by column chromatography (dichloromethane: methanol = 10: 1, v/v) to yield compound **ZT3** as an orange solid (27 mg, 62%). ^1^H NMR (500 MHz, CD_3_OD) δ 8.46 (s, 1H), 8.05 (d, *J* = 7.9 Hz, 1H), 7.86 (d, *J* = 8.8 Hz, 1H), 7.81 – 7.64 (m, 17H), 7.30 – 7.20 (m, 2H), 7.19 (d, *J* = 6.9 Hz, 1H), 6.80 (d, *J* = 9.0 Hz, 1H), 6.64 (s, 1H), 6.52 (d, *J* = 11.3 Hz, 1H), 5.33 (d, *J* = 11.0 Hz, 2H), 4.79 (s, 2H), 3.89 (s, 2H), 3.48 (s, 1H), 3.41 – 3.34 (m, 2H), 3.22 (s, 3H), 3.01 (s, 1H), 2.78 – 2.67 (m, 2H), 2.63 (dt, *J* = 11.9, 6.0 Hz, 3H), 2.25 (t, *J* = 7.4 Hz, 3H), 2.03 (q, *J* = 6.4 Hz, 1H), 1.81 (dt, *J* = 12.4, 7.0 Hz, 2H), 1.67 (s, 2H), 1.60 (s, 1H). ^13^C NMR (126 MHz, CD_3_OD) δ 168.82, 158.56, 155.92, 147.14, 140.48, 137.11, 136.22, 134.80, 131.55, 131.45, 130.84, 130.49, 128.87, 125.07, 124.90, 120.31, 120.08, 119.63, 119.39, 118.66, 113.31, 112.49, 112.38, 111.89, 103.98, 78.80, 78.10, 57.97, 57.11, 55.80, 55.28, 54.69, 40.39, 39.64, 38.71, 36.53, 33.05, 31.93, 30.81, 30.73, 30.61, 30.58, 30.45, 30.31, 28.09, 27.85, 27.71, 26.91, 23.72, 23.31, 23.08, 22.78, 22.37, 14.45, 9.20. HRMS (ESI) calcd for [M]^+^: 1112.3841 (m/z), found 1112.3840.

Synthesis of **D1**: Zinc triflate (Zn(OTf)_2_, 364 mg, 1 mmol) and compound **D0** (294 mg, 2 mmol) were added to acetonitrile (1.04 mL, 20 mmol), followed by the addition of hydrazine monohydrate (4.85 mL, 0.1 mol) under an argon atmosphere. The reaction mixture was stirred at 60^o^C for 24 h. After cooling to room temperature, sodium nitrite (2.76 g, 40 mmol) dissolved in water was added, followed by the slow dropwise addition of 1 M HCl until the pH=3. The resulting mixture was filtered, and the solid was washed with 1 M HCl to obtain the crude product. The crude product was quickly purified by column chromatography (dichloromethane: methanol = 10: 1, v/v) to yield compound **D1** as a purple solid (359 mg, 83%).^[9]^ ^1^H NMR (500 MHz, DMSO-*d6*) δ 8.57 (d, *J* = 8.4 Hz, 2H), 8.20 (d, *J* = 8.5 Hz, 2H), 3.02 (s, 3H).

Synthesis of **D2**: Compound **D1** (100 mg, 0.463 mmol) and N-hydroxysuccinimide (64 mg, 0.556 mmol) were dissolved in of anhydrous dichloromethane (20 mL), followed by the addition of N, N-diisopropylethylamine (DIPEA, 161 µL, 0.926 mmol). The mixture was stirred at room temperature for 4 h. After concentration under reduced pressure, the resulting purple crude product was purified by column chromatography (dichloromethane: methanol = 20: 1, v/v) to yield compound **D2** as a purple solid (117 mg, 81%).^[9]^ ^1^H NMR (500 MHz, CDCl_3_) δ 8.78 – 8.75 (m, 2H), 8.37 – 8.35 (m, 2H), 3.15 (s, 3H), 2.94 (s, 4H).

Synthesis of **ZT4**: Compound **CA2** (30 mg, 0.039 mmol) was dissolved in N, N-dimethylformamide (5 mL), followed by the addition of triethylamine (37 µL). Compound **D2** (28 mg, 0.089 mmol) was added, and the reaction mixture was stirred at room temperature for 2 h. The crude product was purified by column chromatography (dichloromethane: methanol = 5: 1, v/v) to yield compound **ZT4** as a purple solid (34 mg, 90%). ^1^H NMR (500 MHz, CD_3_OD) δ 8.51 (d, *J* = 37.9 Hz, 2H), 8.34 (d, *J* = 8.1 Hz, 2H), 8.04 (d, *J* = 7.9 Hz, 1H), 7.92 (d, *J* = 8.1 Hz, 2H), 7.70 (s, 1H), 7.15 – 7.07 (m, 2H), 6.76 (d, *J* = 9.0 Hz, 2H), 6.62 (s, 2H), 6.46 (d, *J* = 6.7 Hz, 2H), 5.49 (s, 1H), 5.34 (t, *J* = 4.9 Hz, 1H), 5.19 (s, 2H), 4.78 (d, *J* = 2.4 Hz, 2H), 3.85 (s, 3H), 3.66 (s, 1H), 3.59 (d, *J* = 6.1 Hz, 2H), 3.56 (t, *J* = 5.7 Hz, 2H), 3.35 (s, 2H), 3.04 (s, 3H), 3.00 (t, *J* = 2.4 Hz, 1H), 2.84 (q, *J* = 6.5 Hz, 4H), 2.76 (t, *J* = 5.8 Hz, 2H). ^13^C NMR (126 MHz, CD_3_OD) δ 176.07, 169.06, 168.91, 164.51, 158.57, 155.78, 147.02, 140.21, 138.74, 136.88, 136.08, 131.47, 130.85, 130.40, 129.16, 128.69, 128.29, 113.46, 112.31, 111.53, 103.85, 78.78, 78.00, 64.47, 57.95, 57.02, 55.30, 54.80, 54.61, 49.85, 39.45, 39.26, 30.82, 30.73, 30.61, 30.46, 30.31, 28.09, 26.25, 21.18. HRMS (ESI) calcd for [M+H]^+^: 966.2980 (m/z), found 966.3071.

Synthesis of **TCO-CHA**:

CHA-dde (154.3 mg, 0.2 mmol) was dissolved in acetonitrile (30 mL), followed by the addition of DIPEA (200 µL). The mixture was stirred for 15 min, after which TCO-PEG_4_-NHS (102.8 mg, 0.2 mmol) was added and allowed to react at room temperature for 12 h. The solvent was removed under reduced pressure to afford **TCO-CHA-dde** as a white solid, which was characterized by high-resolution mass spectrometry (HRMS). HRMS (ESI) calcd for [M+H]^+^: 1171.7628 (m/z), found 1171.7683.

The obtained white solid was then dissolved in dichloromethane (30 mL) containing 2% hydrazine hydrate and stirred at room temperature for 2 h. After removal of the solvent under vacuum, and the peptide was precipitated with cold diethyl ether. The crude product was purified by preparative thin-layer chromatography to afford **TCO-CHA** as a white solid. HRMS (ESI) calcd for [M+H]^+^: 1007.6791 (m/z), found 1007.6869.

Synthesis of **MB-N_3_**

MB-NH_2_ (2 mg; bma500-2, BioMag^TM^) with a surface amino modification degree of 3.1 nmol mg^–1^ (as provided by the supplier) was washed three times with N, N-dimethylformamide (1 mL each) and sonicated for 5 min. Subsequently, N_3_-NHS (54 μg, 240 nmol) and an appropriate amount of triethylamine were added, and the mixture was stirred for 4 h. The resulting product was washed three times with N, N-dimethylformamide (1 mL each) to afford **MB-N_3_**.

Synthesis of **LAMI1-LAMI3** and **P-LAMI4**

Four aliquots of the above-prepared **MB-N_3_** were each dispersed in DMSO: H_2_O (1:1, 1 mL) solution, followed by the addition of **ZT1-4** (240 nmol) and thorough mixing. A premixed solution of CuSO_4_ (10 mM, 8 μL), THPTA (60 mM, 8 μL), and sodium ascorbate (100 mM, 40 μL) was then introduced into the reaction system. The mixture was incubated for 12 h. The resulting products were washed with ethanol (3×) to obtain **LAMI1**-**LAMI3** and **P-LAMI4**, respectively. Finally, the products were dispersed in ethanol and stored at 4^o^C.

**3. Supplementary tables and figures**

**
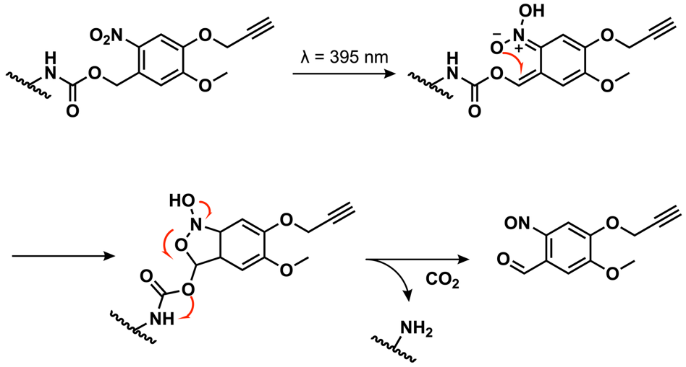
**

**Figure S1.** Schematic illustration of the photo-cleavage mechanism of the linker in light-responsive multifunctional probes.

**
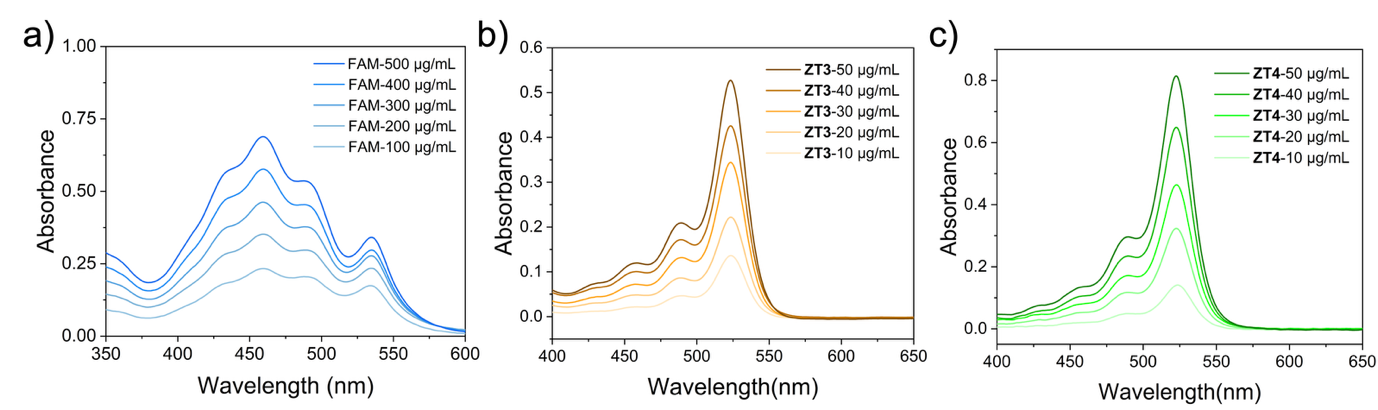
**

**Figure S2.** Ultraviolet-visible absorption spectra. a) 5-FAM fluorescence molecules at different concentrations. b) **ZT3** at different concentrations. c) **ZT4** at different concentrations. Measurements were performed in the solvent DMF.


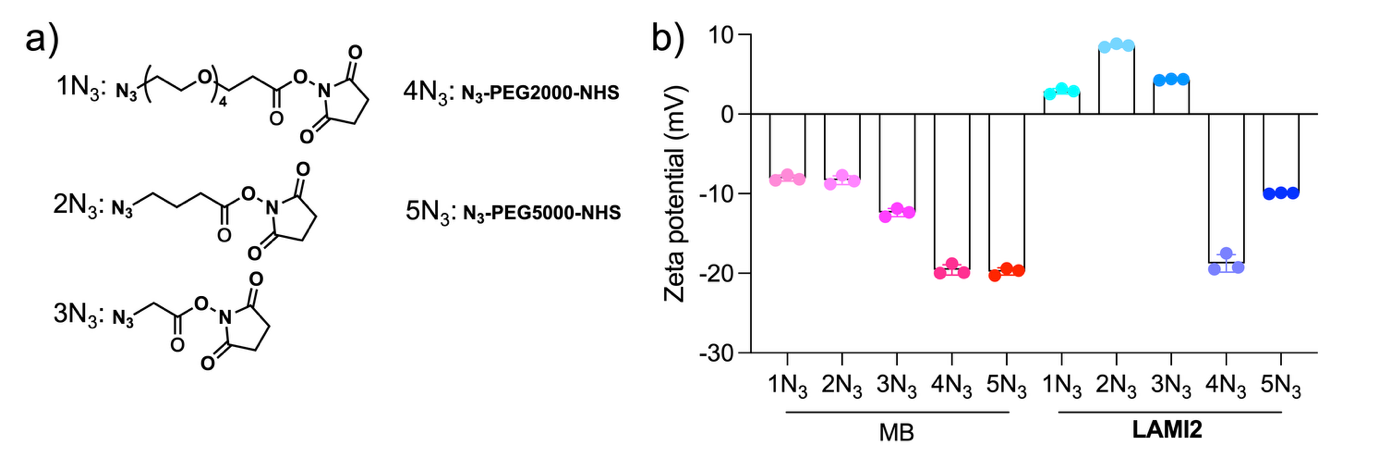


**Figure S3.** a) Chemical structures of 1–5N_3_-NHS. b) Zeta potentials of MB and **LAMI2** after modification with azides 1N_3_–5N_3_.


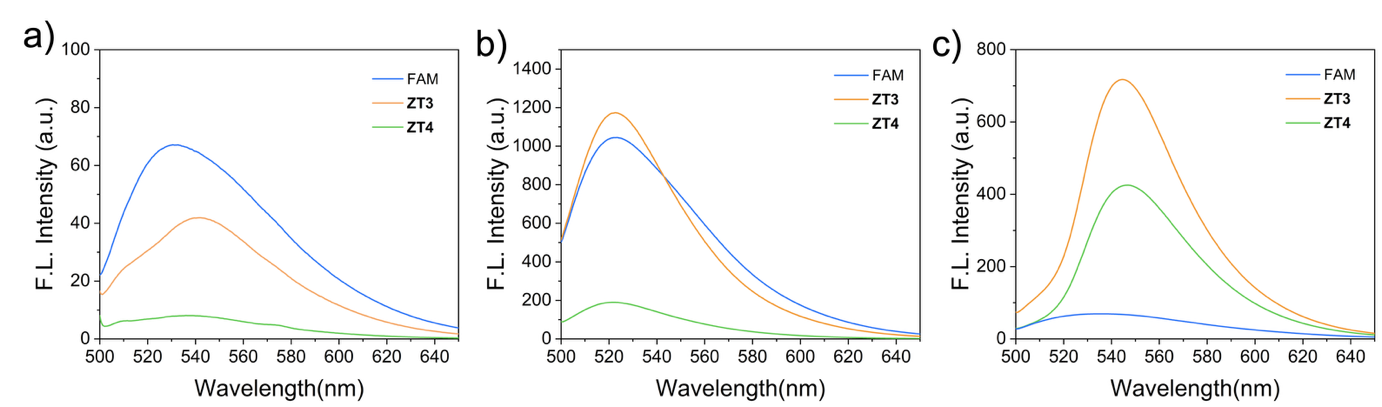


**Figure S4*.*** Fluorescence emission spectra of FAM, **ZT3**, and **ZT4** in different solvents. a) DMSO, b) MeOH, c) DMF. Ex = 488 nm.

**
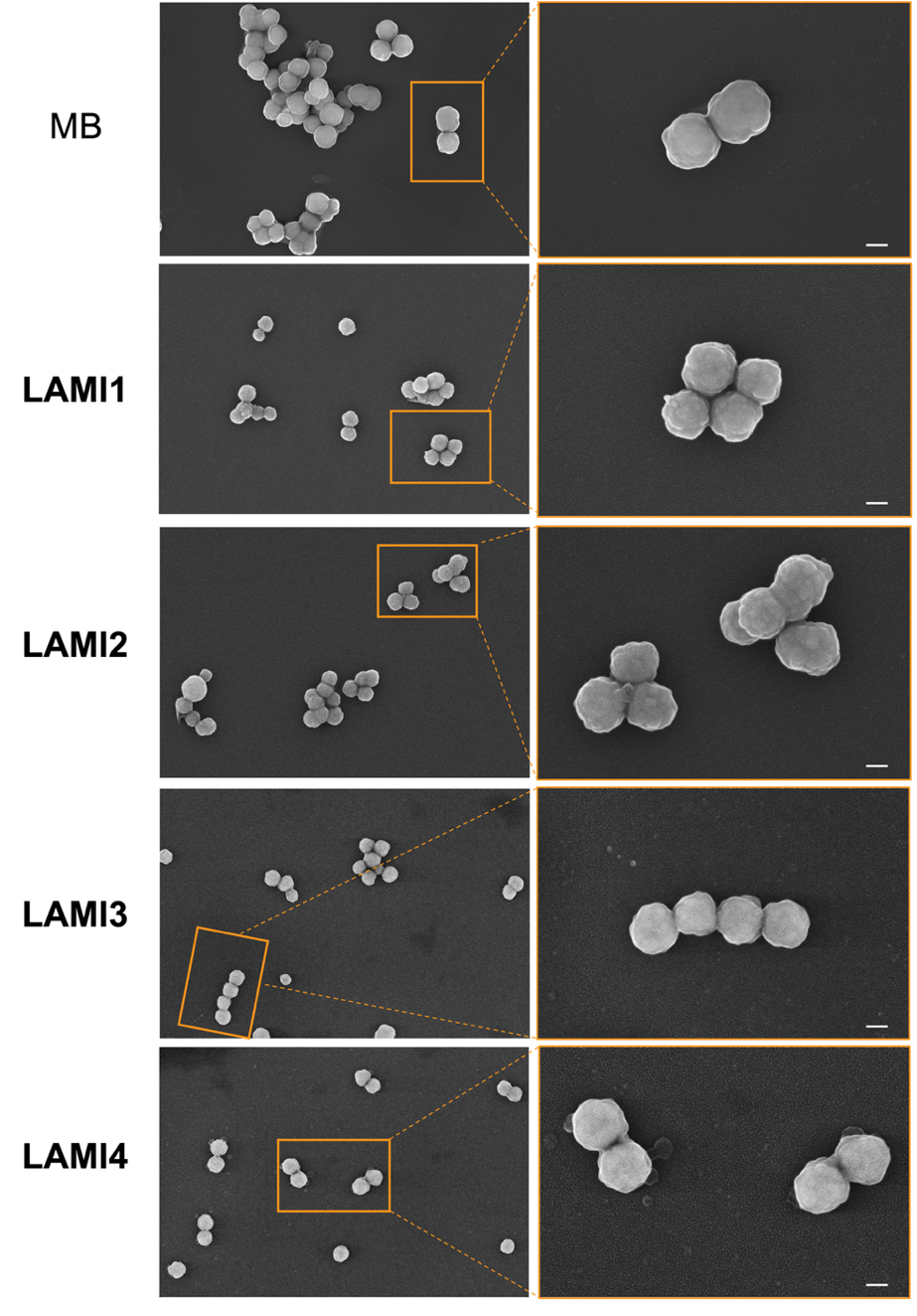
**

**Figure S5.** SEM images of the **LAMI** beads (MB, **LAMI1**–**LAMI4**), scale bar = 100 nm.


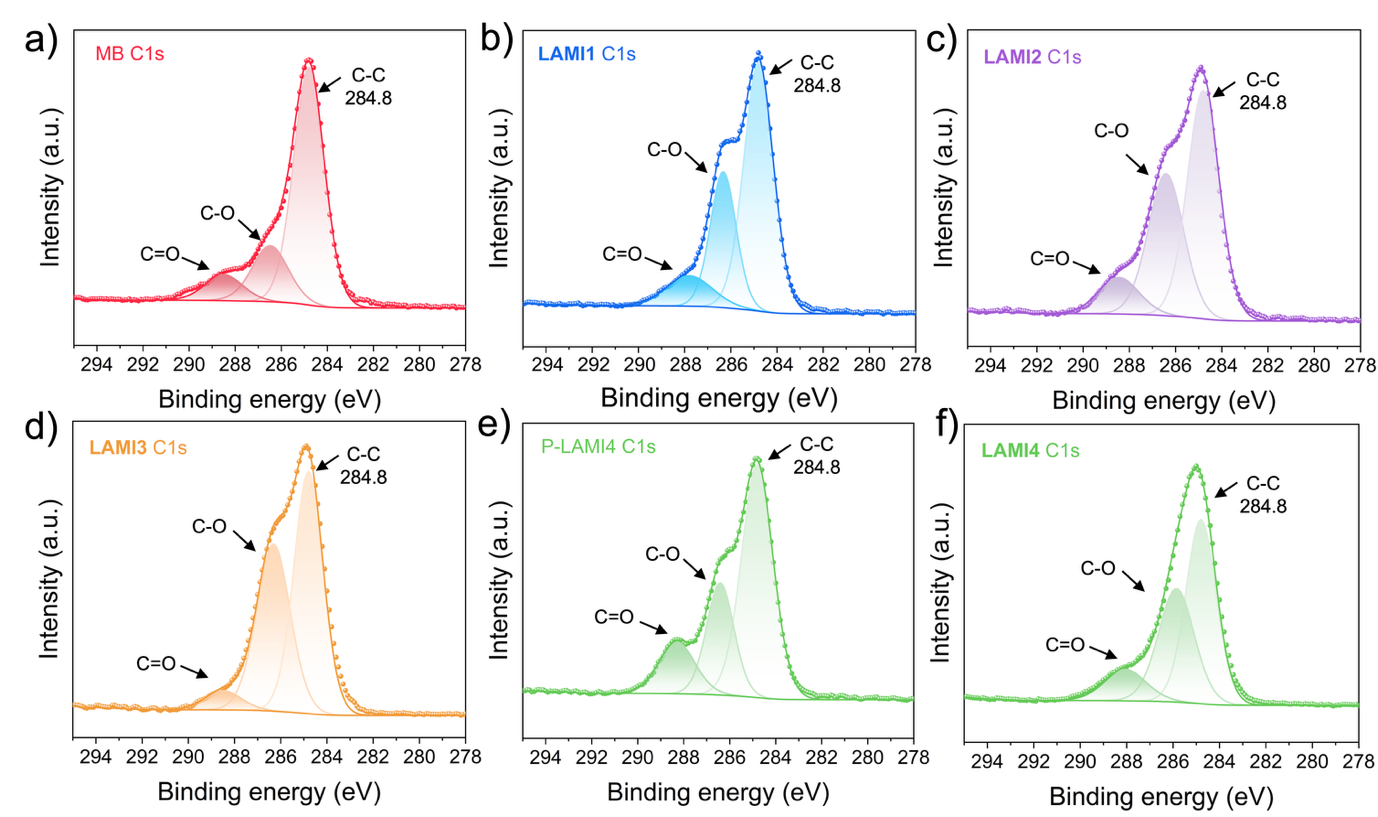


**Figure S6.** X-ray photoelectron spectroscopy (XPS) C1s analysis of the **LAMI** beads. a) MB, b) **LAMI1**, c) **LAMI2**, d) **LAMI3**, e) P-LAMI4, f) **LAMI4**.


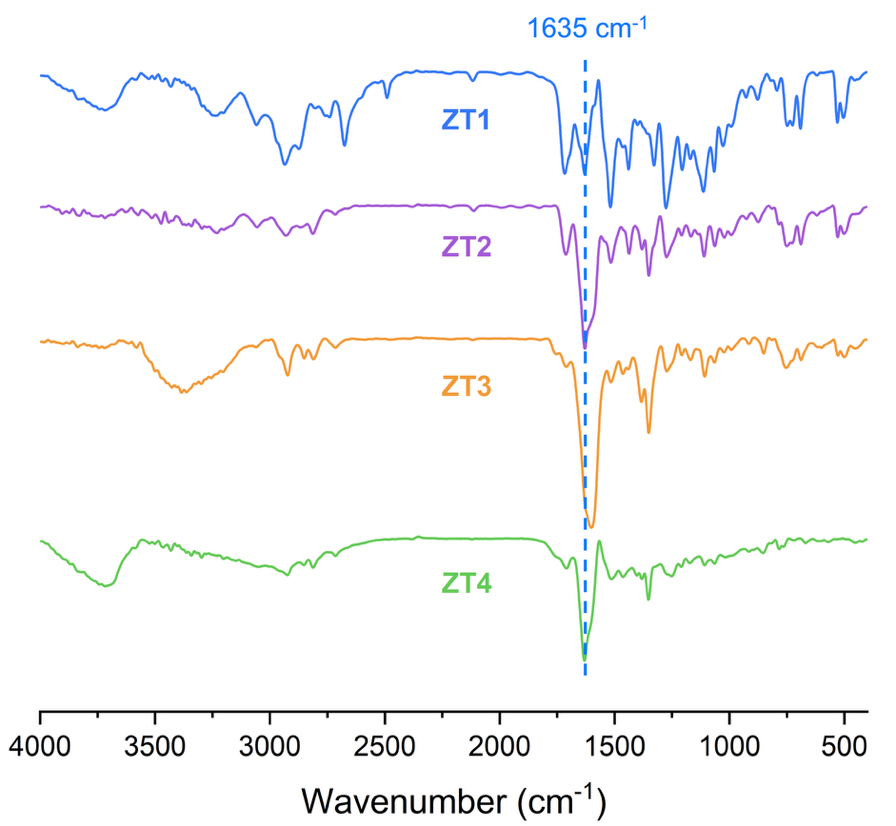


**Figure S7.** Fourier-transform infrared (FTIR) spectra of **ZT1–ZT4** molecules.


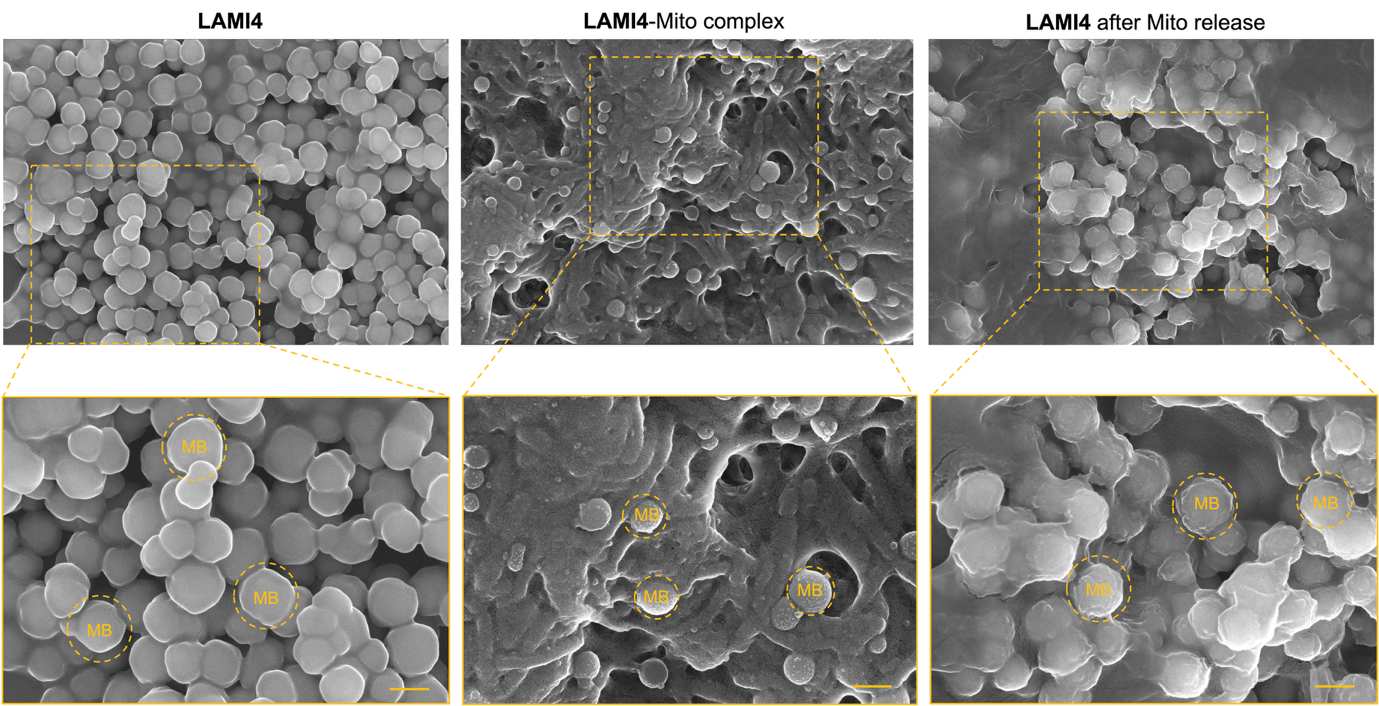


**Figure S8.** Verification of mitochondrial isolation and purification using the **LAMI** platform. Bio-scanning electron microscopy (SEM) images of **LAMI4** beads at three stages of mitochondrial capture, scale bar = 200 nm.


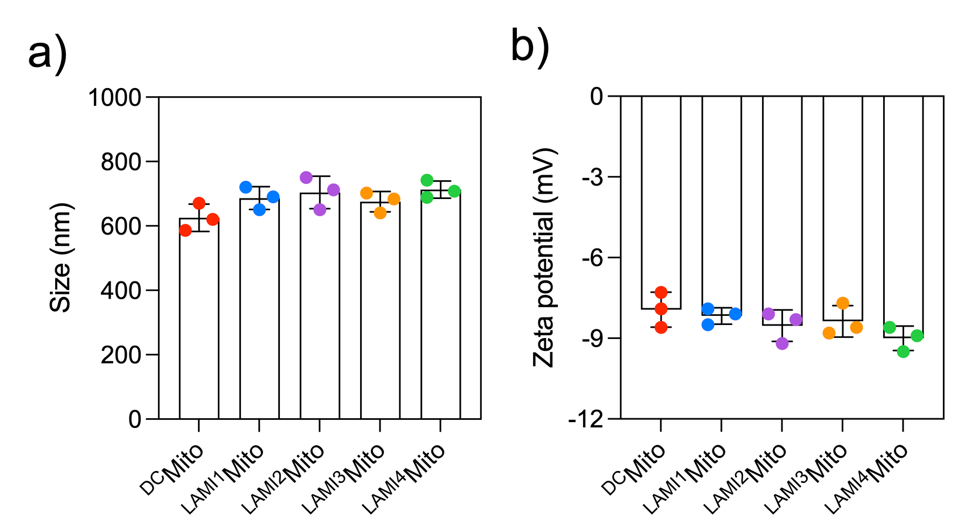


**Figure S9.** Size (a) and Zeta potential (b) characterization of the **LAMI**-based method for mitochondrial isolation from HepG2 cells (n = 3).

**Figure S10**. Evaluation of potential phototoxicity induced by 395 nm irradiation. Mitochondria isolated *via* the DC method were either kept in the dark (^DC^Mito) or exposed to 395 nm light (^DC^Mito+UV, 5 mW cm^–2^, 2 min; total energy density 0.6 J cm^–2^) to precisely mimic the **LAMI** photo-cleavage conditions. a) Quantitative analysis of ROS levels (assessed by MitoSOX) and b) FACS analysis of MitoSOX fluorescence (Violet fluorescence detection channel, 610 nm). c) Quantitative analysis of MMP and d) FACS analysis of TMRM fluorescence (PE channel). e) Quantitative analysis of ATP production. All data are presented as mean ± s.d. (n = 3 independent biological replicates). The *p*-values were determined by an unpaired Student's t-test.


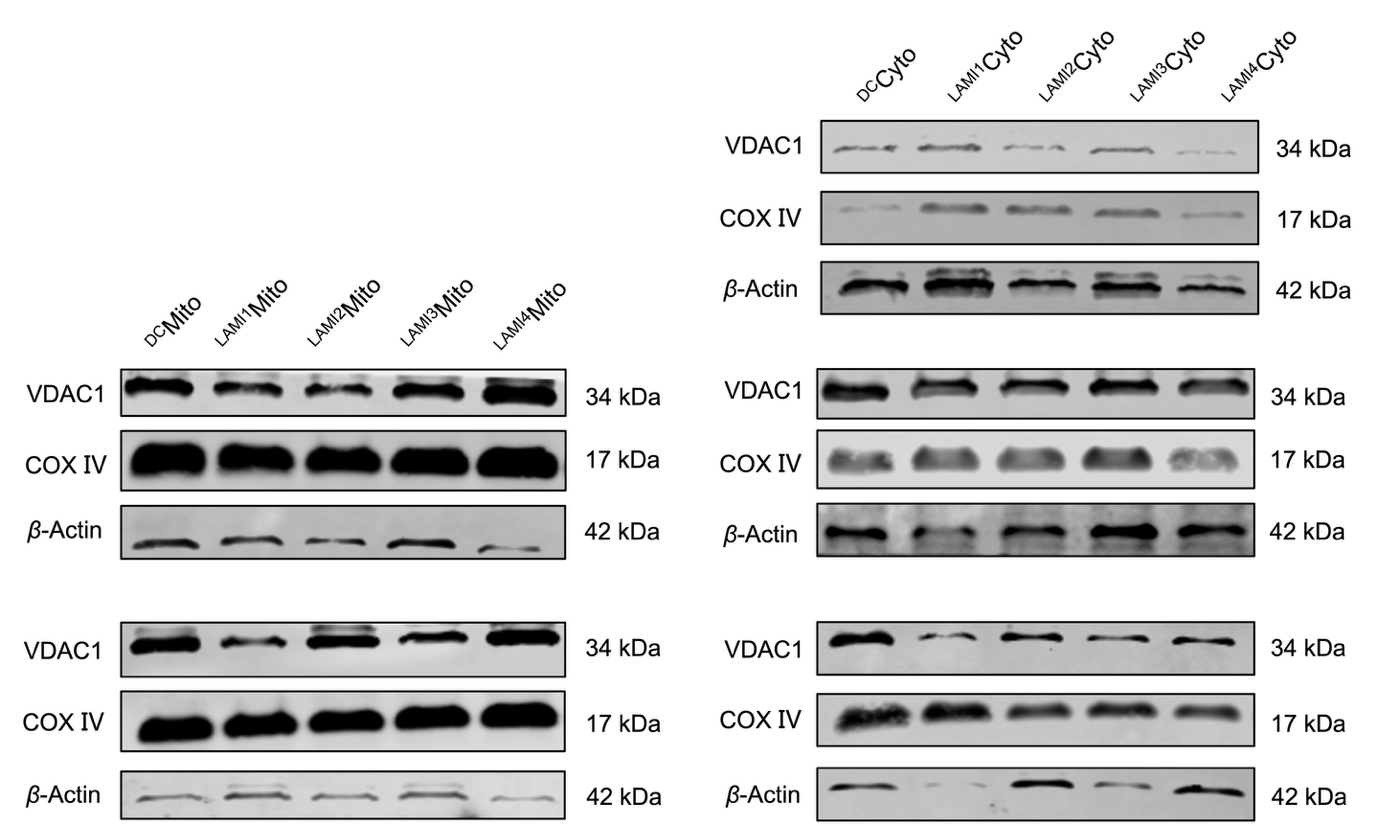


**Figure S11.** Western blot analysis of VDAC1 and COX IV following mitochondrial isolation by the DC method and the **LAMI**-based method. a) Protein levels of VDAC1 and COX IV in mitochondria isolated by the two methods. b) Protein levels of VDAC1 and COX IV in the corresponding cytoplasmic fractions. The higher levels detected in the DC-derived cytoplasm indicate increased mitochondrial damage and/or lower isolation efficiency compared to the **LAMI**-based method.


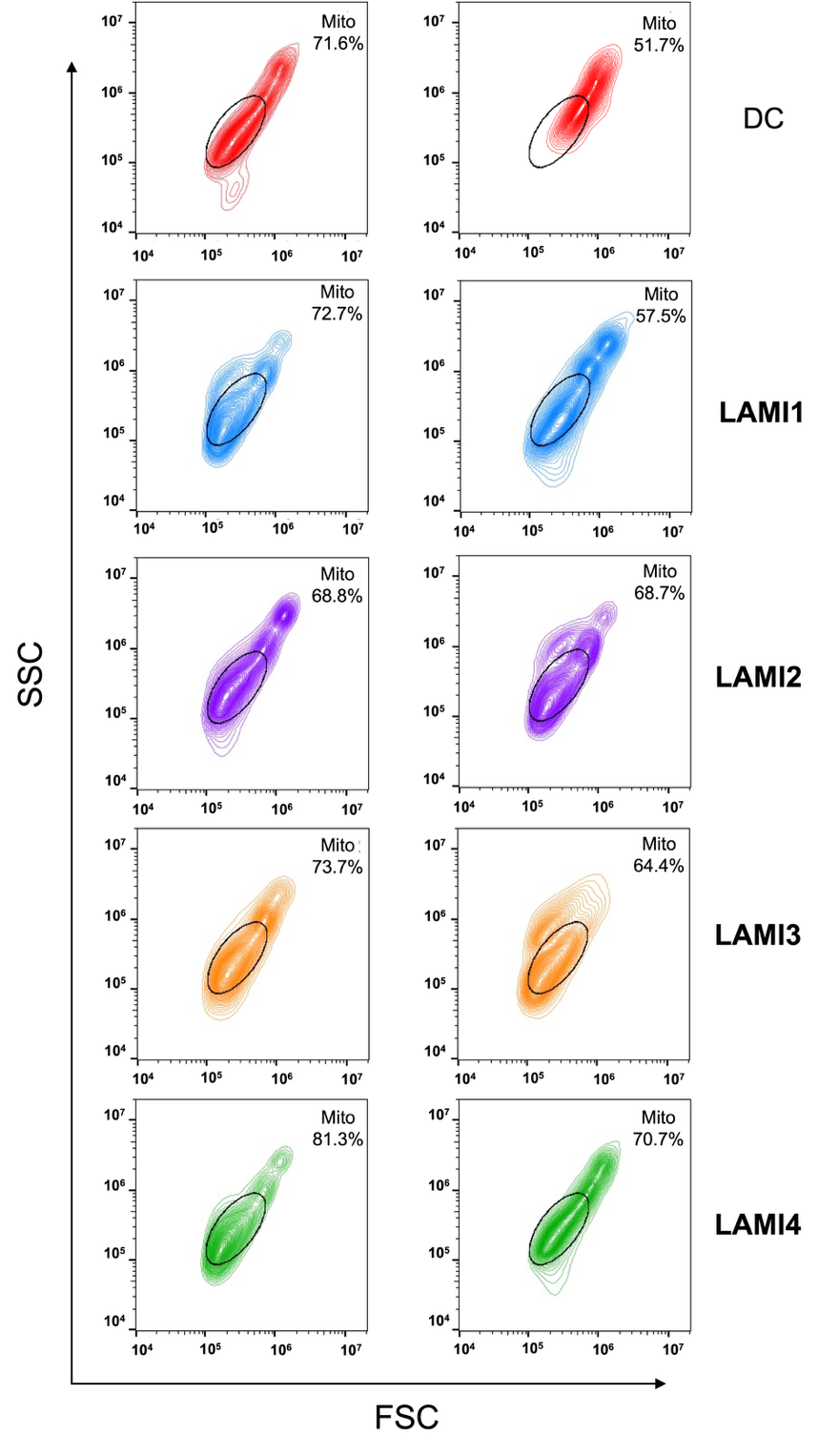


**Figure S12.** FACS scatter plots of mitochondria isolated by the DC method and **LAMI**-based method.


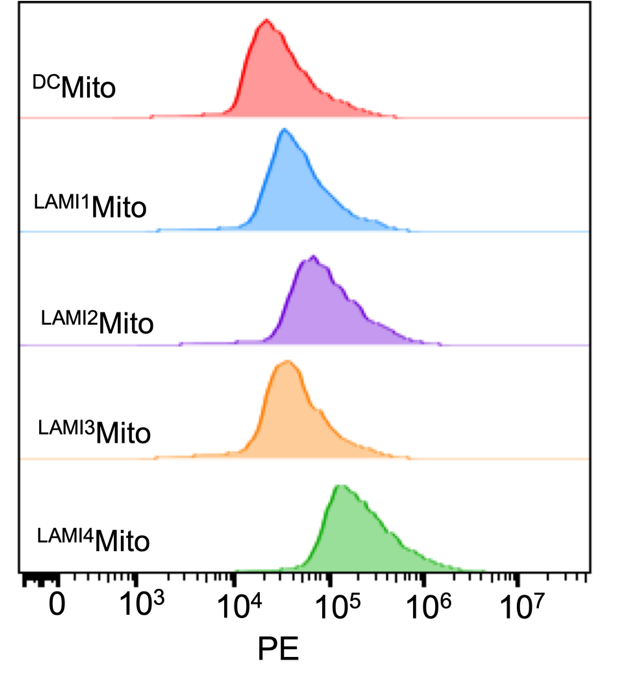


**Figure S13*.*** FACS analysis of PE fluorescence detection channels of mitochondria isolated by the DC method and **LAMI**-based method.


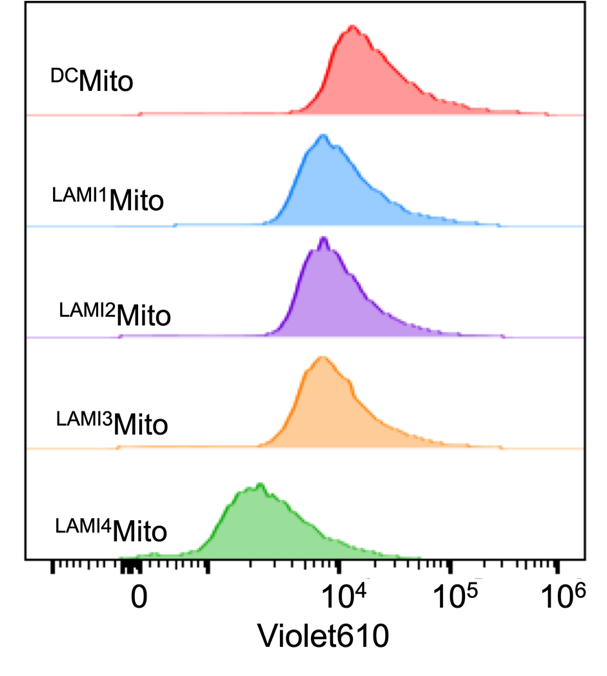


**Figure S14.** FACS analysis of violet fluorescence detection channels (610 nm) of mitochondria isolated by the DC method and **LAMI**-based method.


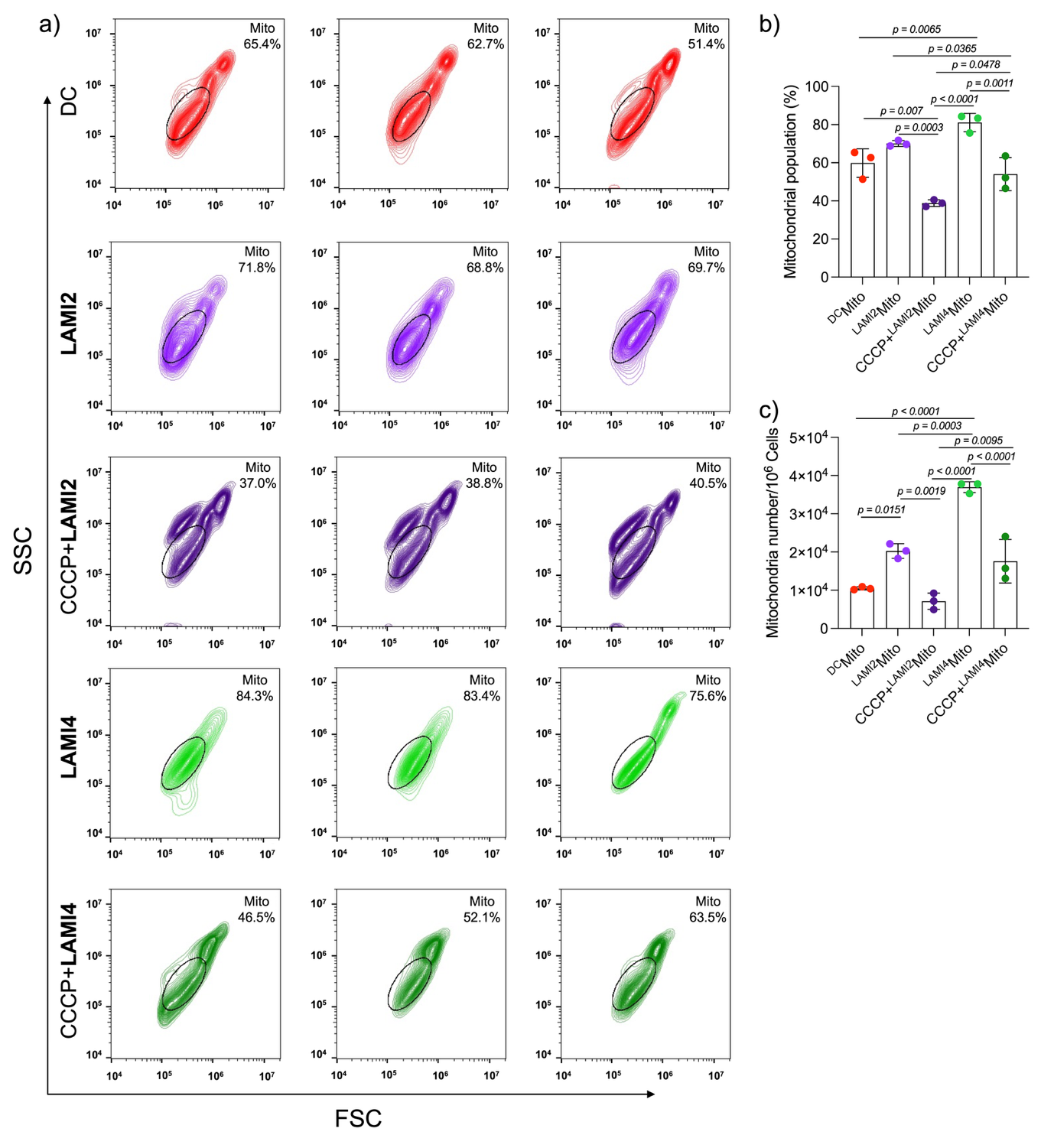


**Figure S15**. Flow cytometry analysis of mitochondrial capture efficiency with or without CCCP pretreatment. a) FACS scatter plots of mitochondria isolated using the DC, **LAMI2**, and **LAMI4**-based methods, with and without prior CCCP treatment, respectively. b) Mitochondrial population derived from the FACS scatter plots. c) Quantification of mitochondrial particle numbers within the FACS gate for equal sample volumes. For the CCCP-treated groups, cells were pre-incubated with 10 µM CCCP for 15 min prior to mitochondrial isolation *via* the respective methods. Data are presented as mean ± s.d. (n = 3 independent biological replicates). The *p*-values were determined by one-way ANOVA with Tukey’s multiple comparison test.

**
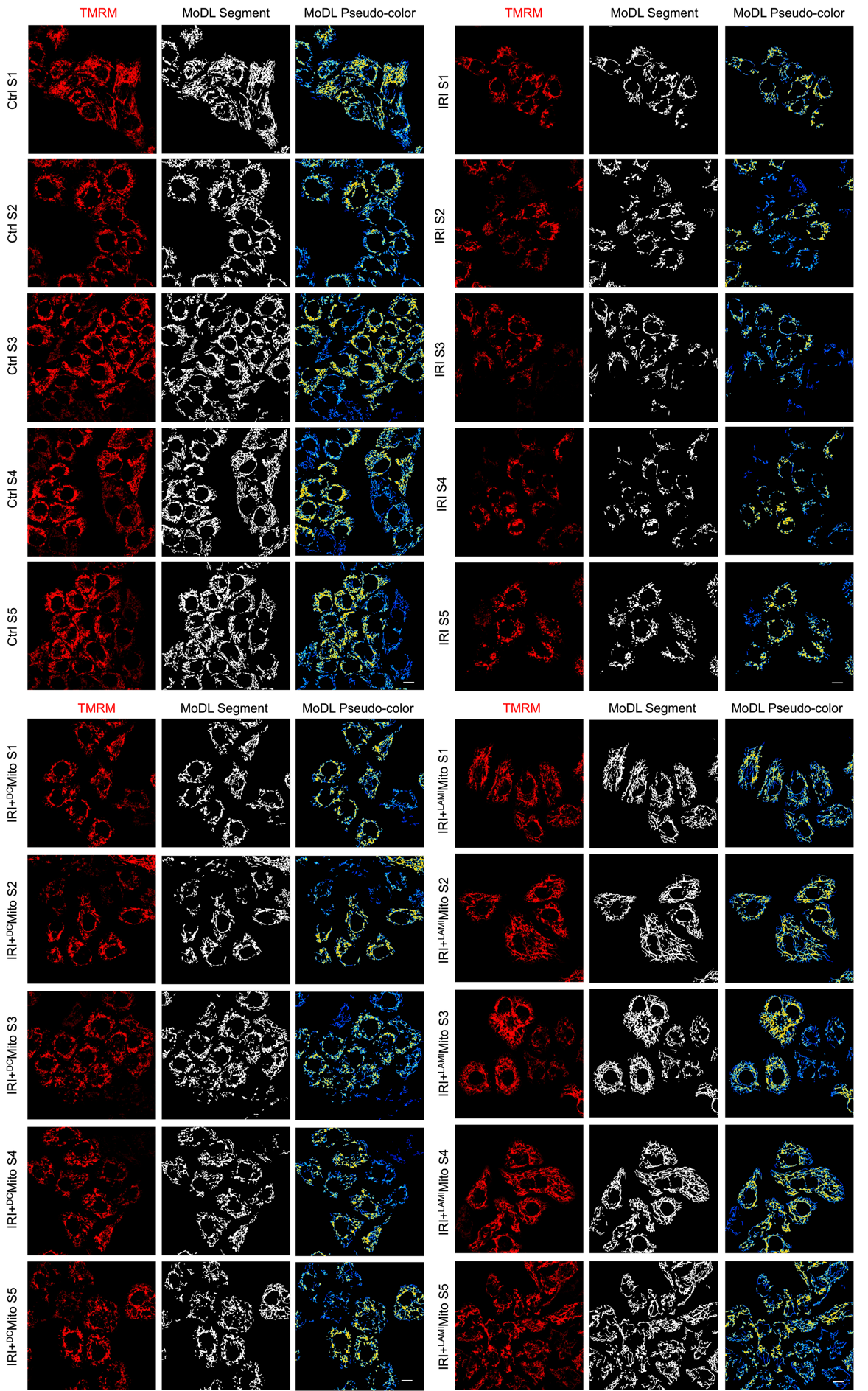
**

**Figure S16.** Analysis of mitochondrial TMRM fluorescence using the MoDL deep learning model. Shown are the original fluorescence channel (left), the MoDL-generated segmentation mask (middle), and the corresponding pseudo-colored visualization of fluorescence intensity (right). (n = 5), Scale bar = 10 µm.


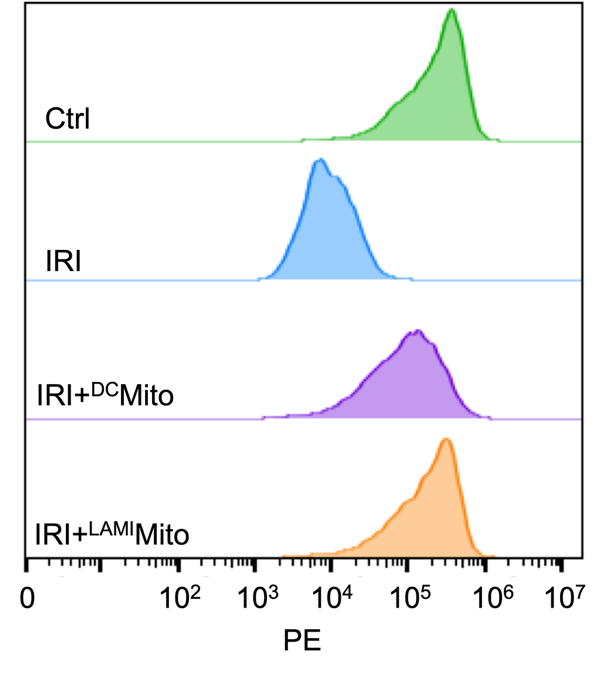


**Figure S17*.*** FACS analysis (PE channel) of mitochondrial membrane potential in hepatocytes following mitochondrial transplantation in an *in vitro* hepatic IRI model.


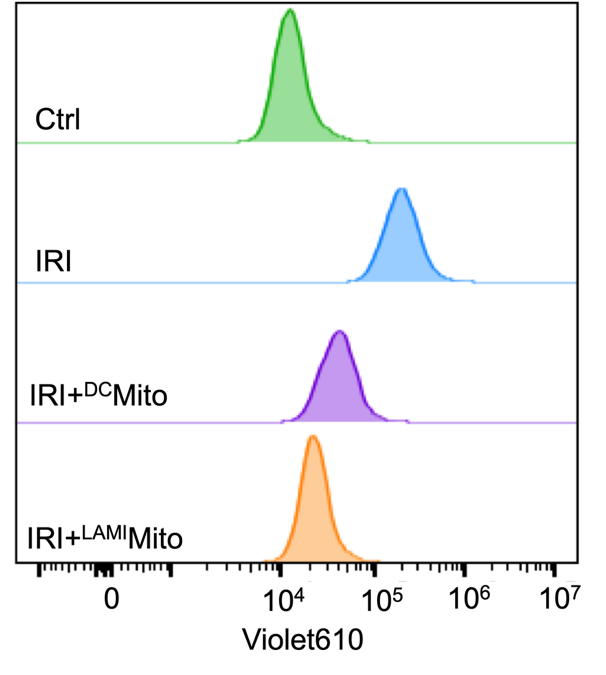


**Figure S18.** FACS analysis (Violet fluorescence detection channels, 610 nm) of ROS levels in hepatocytes following mitochondrial transplantation in an *in vitro* hepatic IRI model.


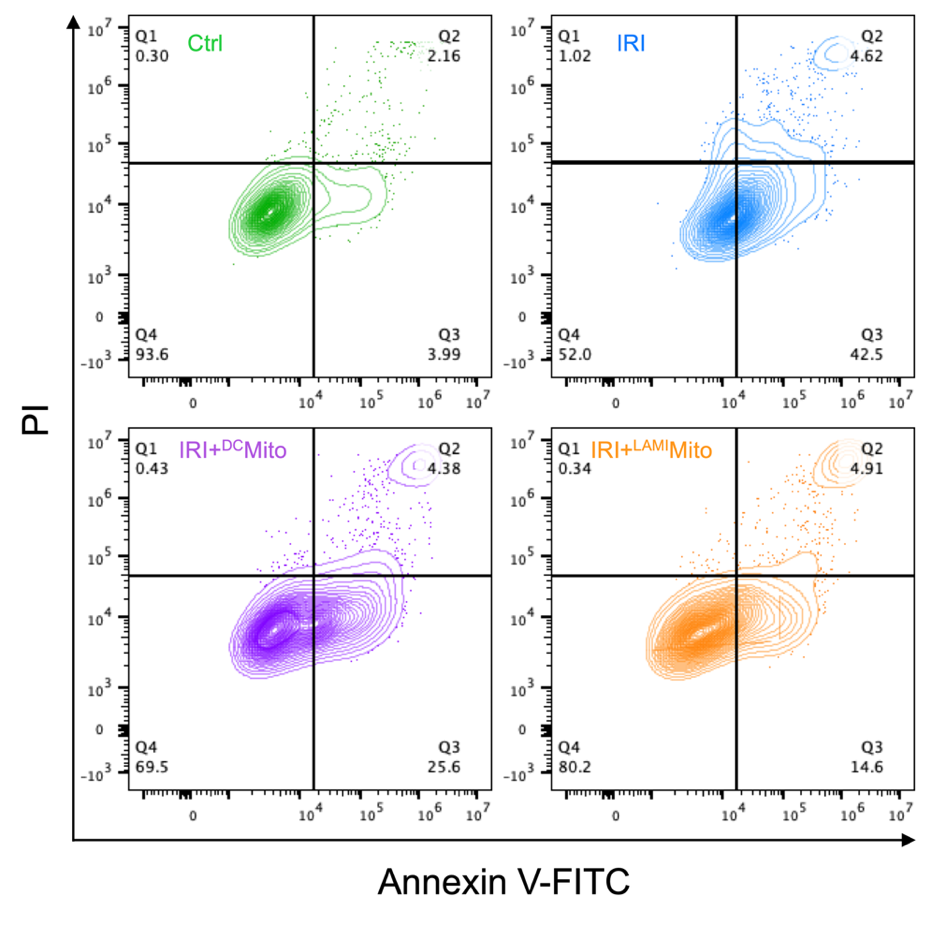


**Figure S19*.*** Representative FACS scatter plots of apoptotic cells after mitochondrial transplantation in an *in vitro* IRI model are shown, based on Annexin V–FITC/PI dual staining. Cells were divided into four quadrants: the lower left quadrant (Q4, Annexin V⁻/PI⁻) corresponds to viable cells; the lower right quadrant (Q3, Annexin V⁺/PI⁻) indicates early apoptotic cells; and the upper right quadrant (Q2, Annexin V⁺/PI⁺) represents late apoptotic or necrotic cells.


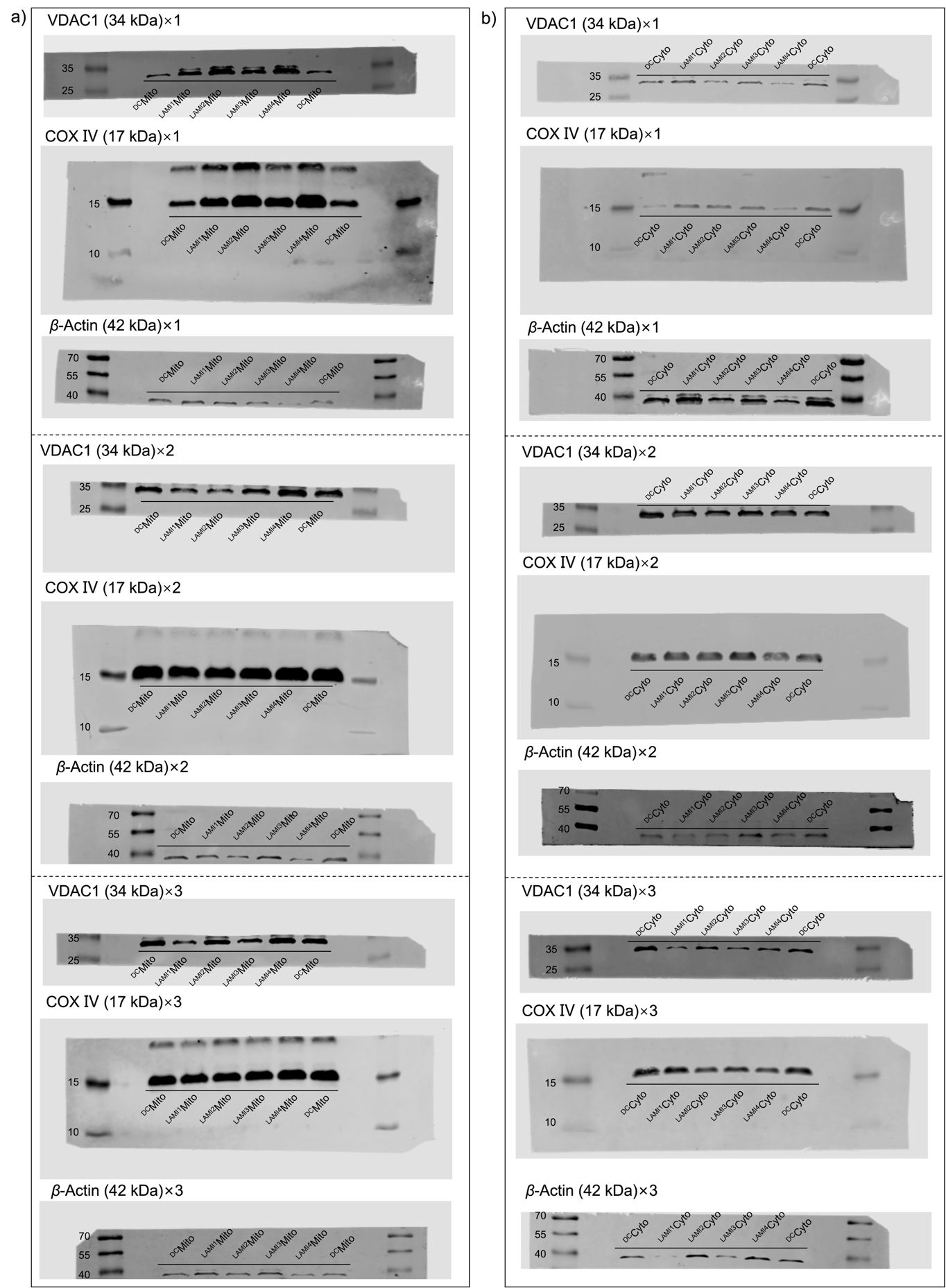


**Figure S20.** Uncropped blot for Figure 3b and Figure S11. a) Expression of VADC1 and COX IV proteins in mitochondria separated by the DC method and **LAMI**-based method. b) Expression analysis of VADC1 and COX IV proteins in the separated cytoplasm.

**4. ^1^H and ^13^C NMR spectra of all compounds**


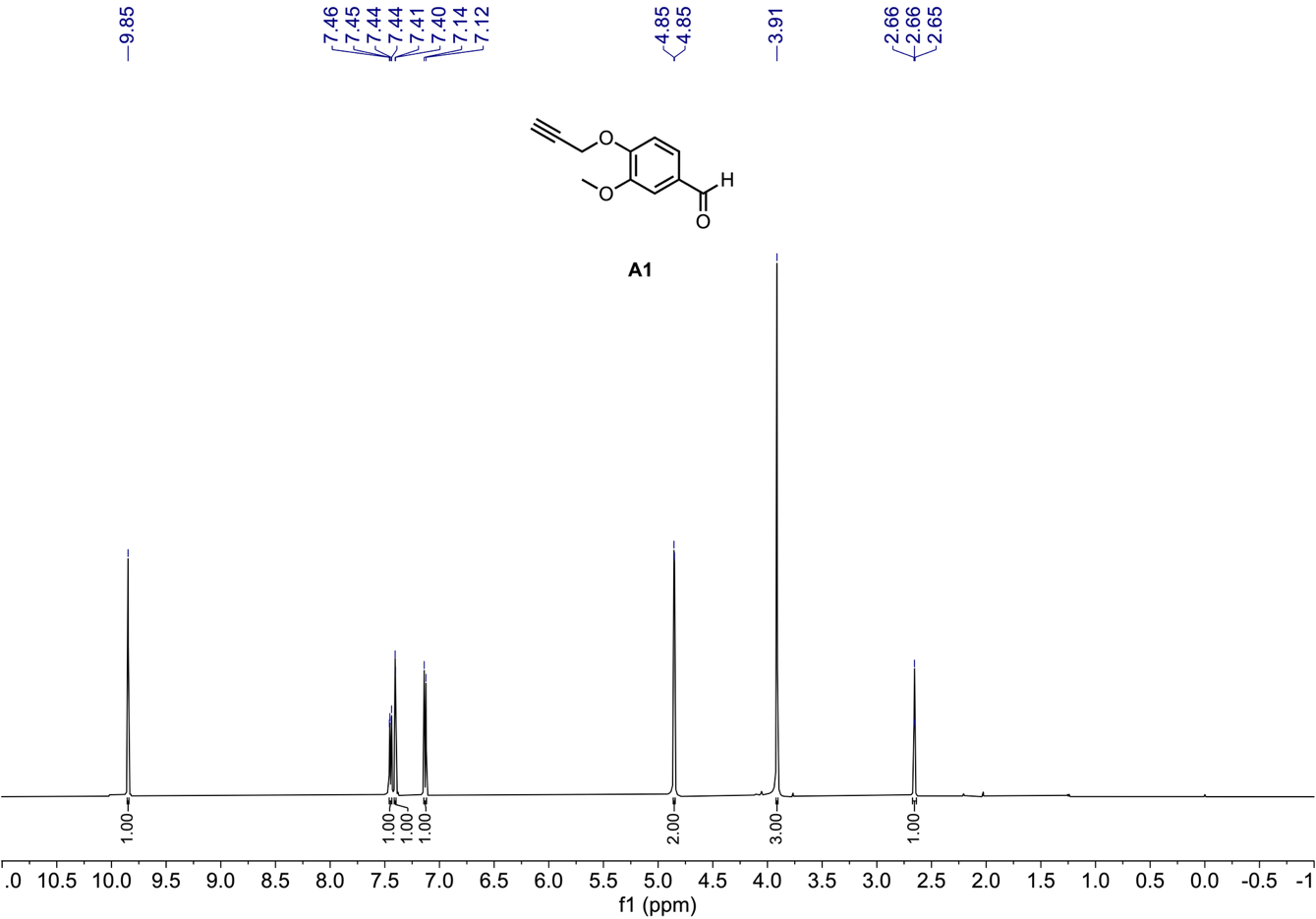


**Figure S21*.*** ^1^H NMR spectrum of compound **A1**.


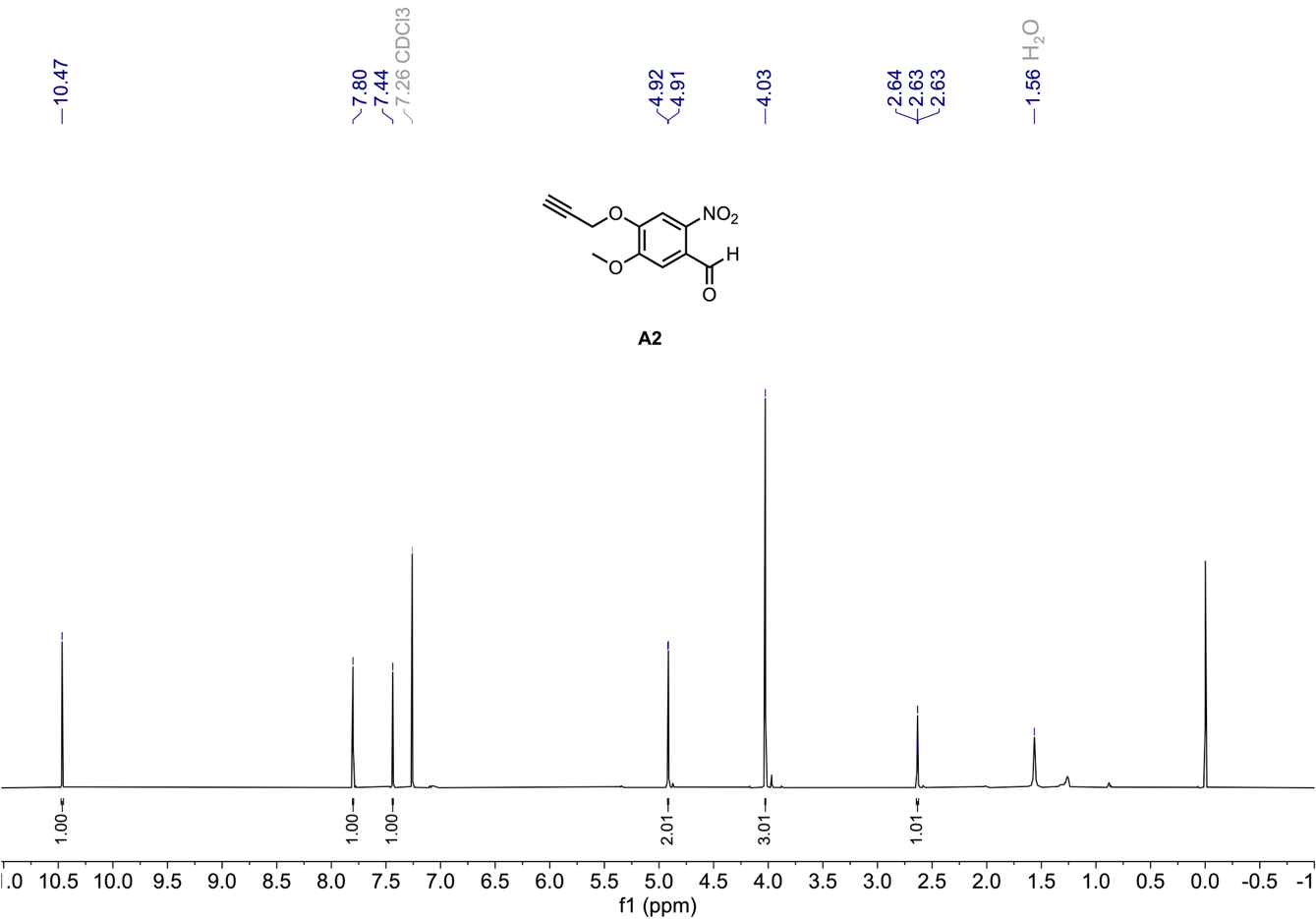


**Figure S22.** ^1^H NMR spectrum of compound **A2**.

**
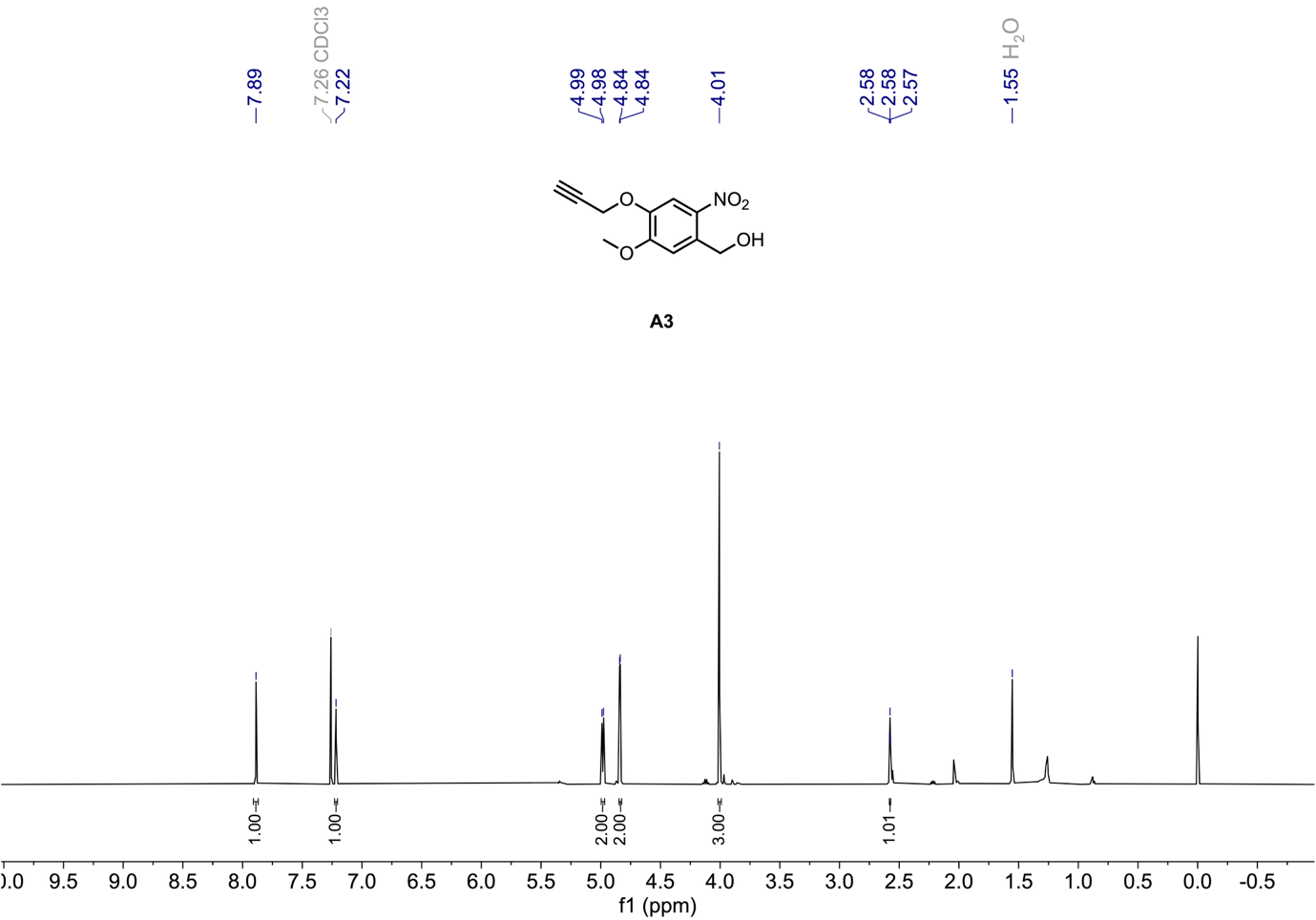
**

**Figure S23*.*** ^1^H NMR spectrum of compound **A3**.

**
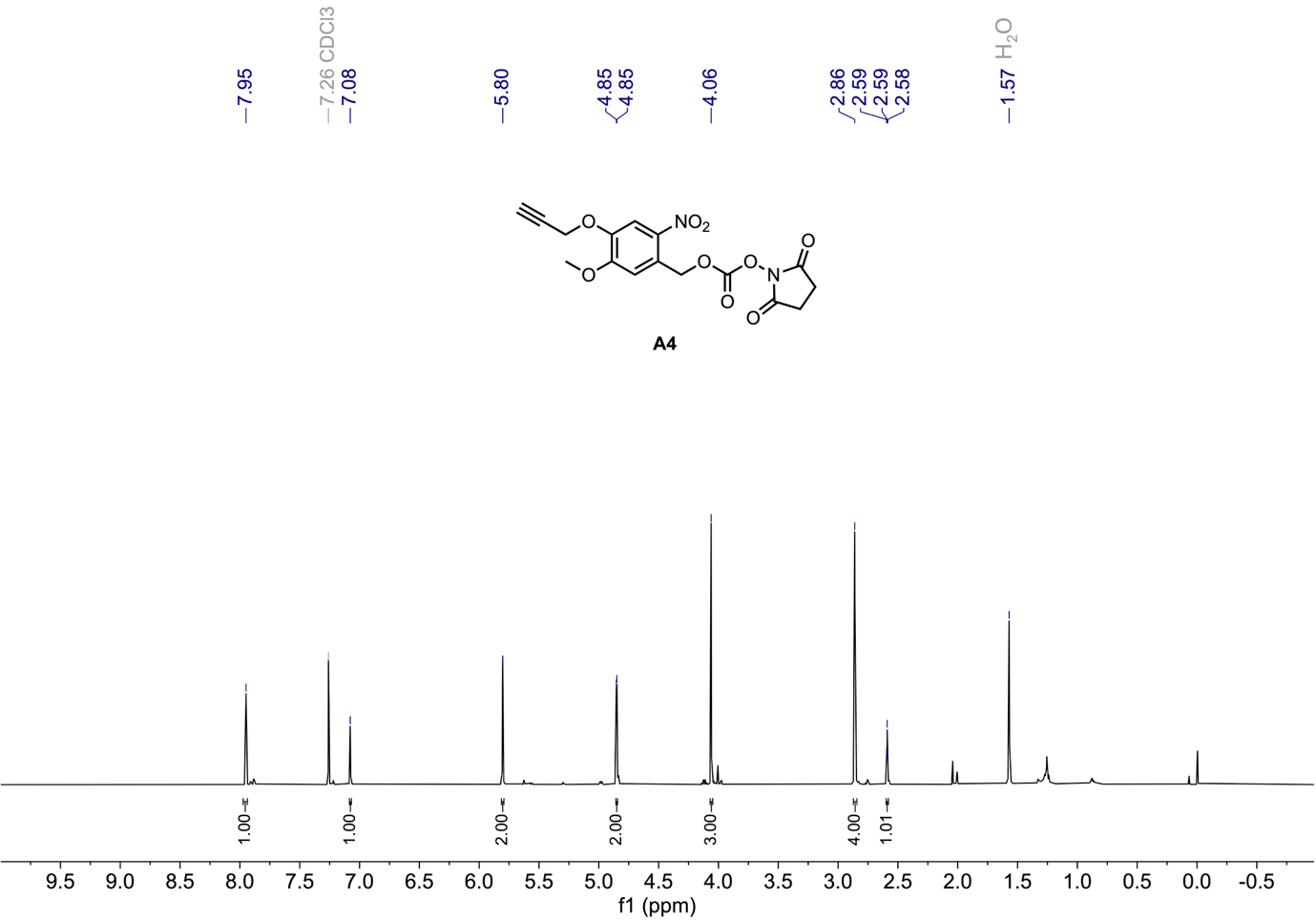
**

**Figure S24*.*** ^1^H NMR spectrum of compound **A4**.

**
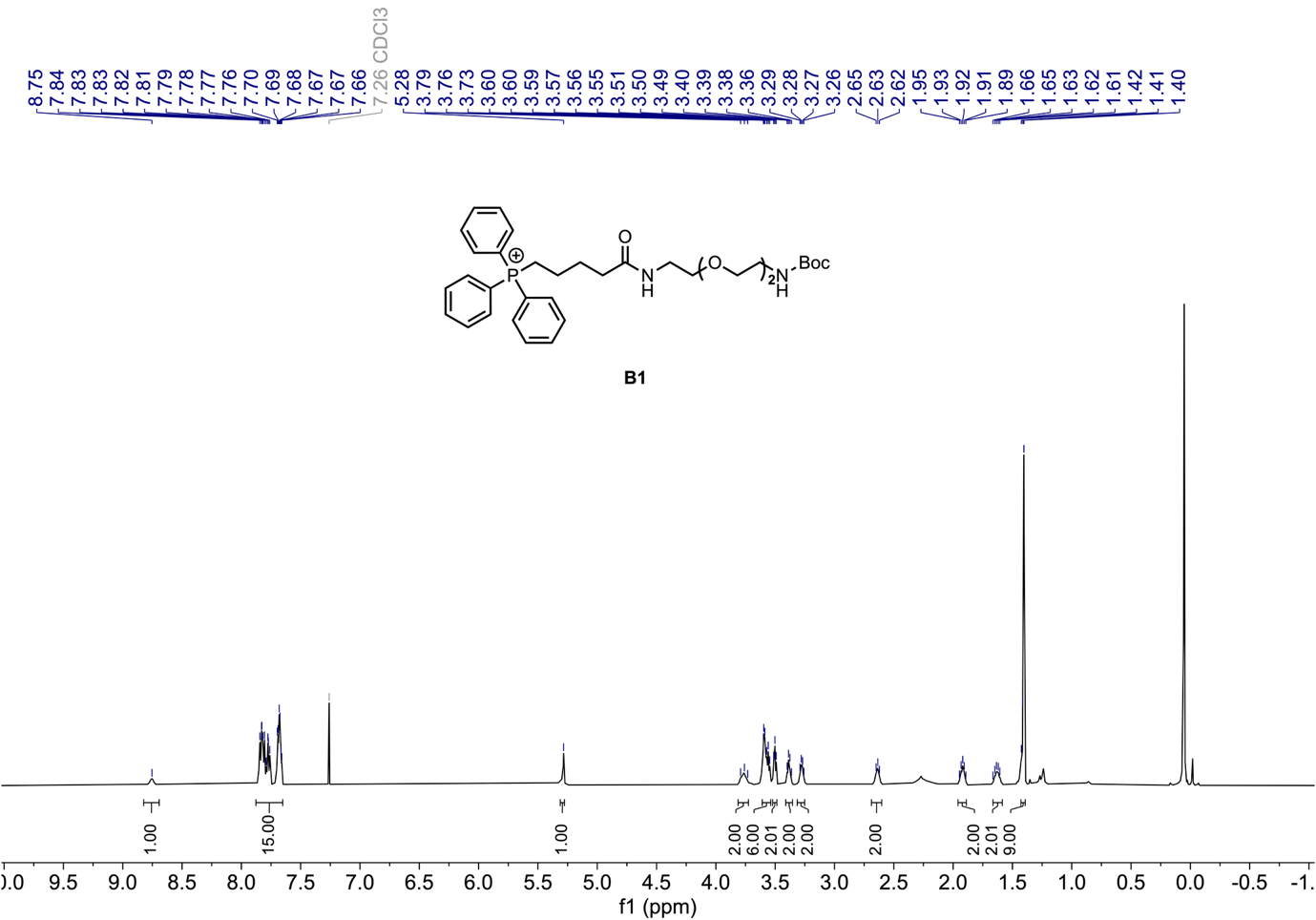
**

**Figure S25*.*** ^1^H NMR spectrum of compound **B1**.


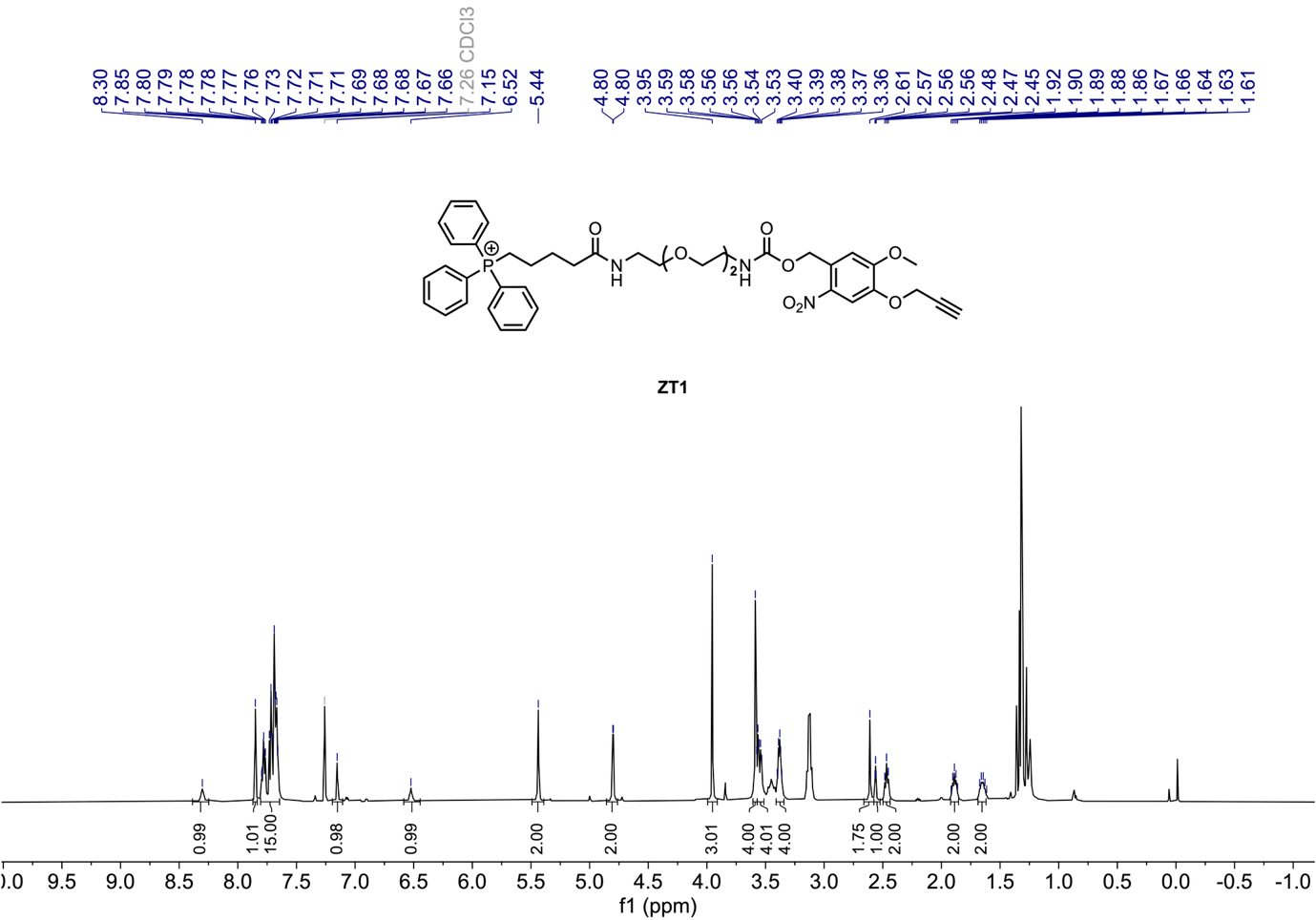


**Figure S26*.*** ^1^H NMR spectrum of compound **ZT1**.

**
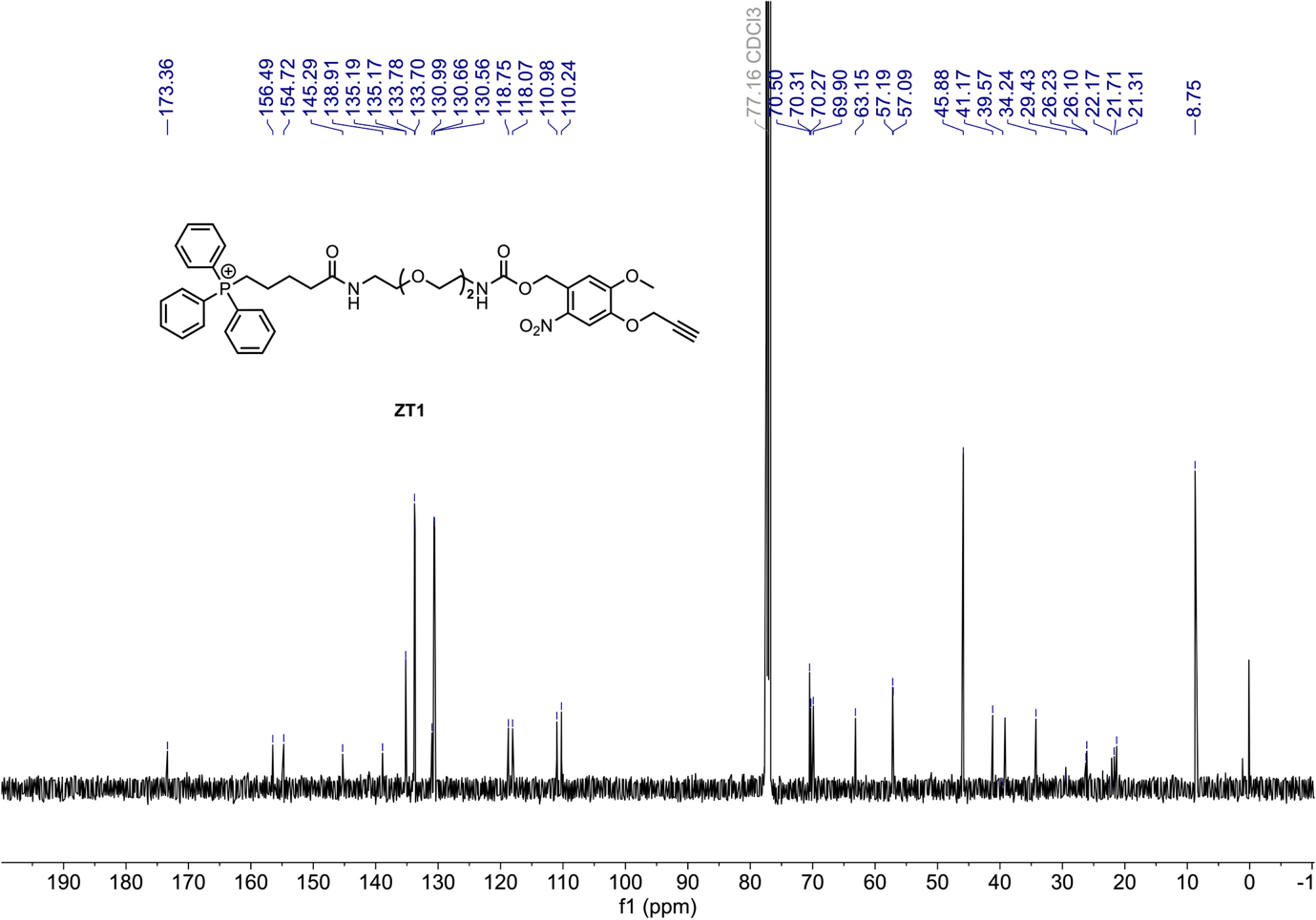
**

**Figure S27*.*** ^13^C NMR spectrum of compound **ZT1**.

**
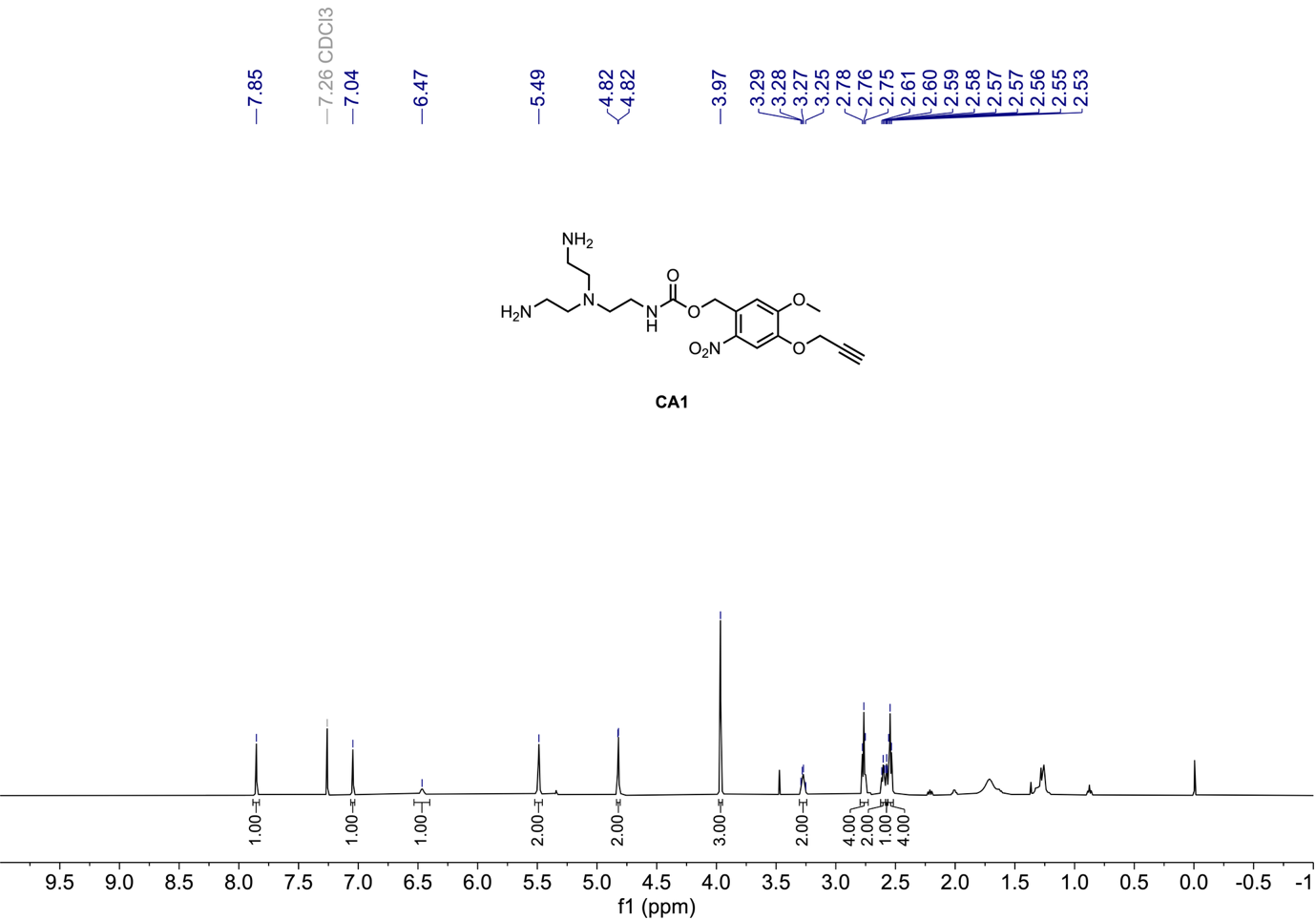
**

**Figure S28*.*** ^1^H NMR spectrum of compound **CA1**.

**
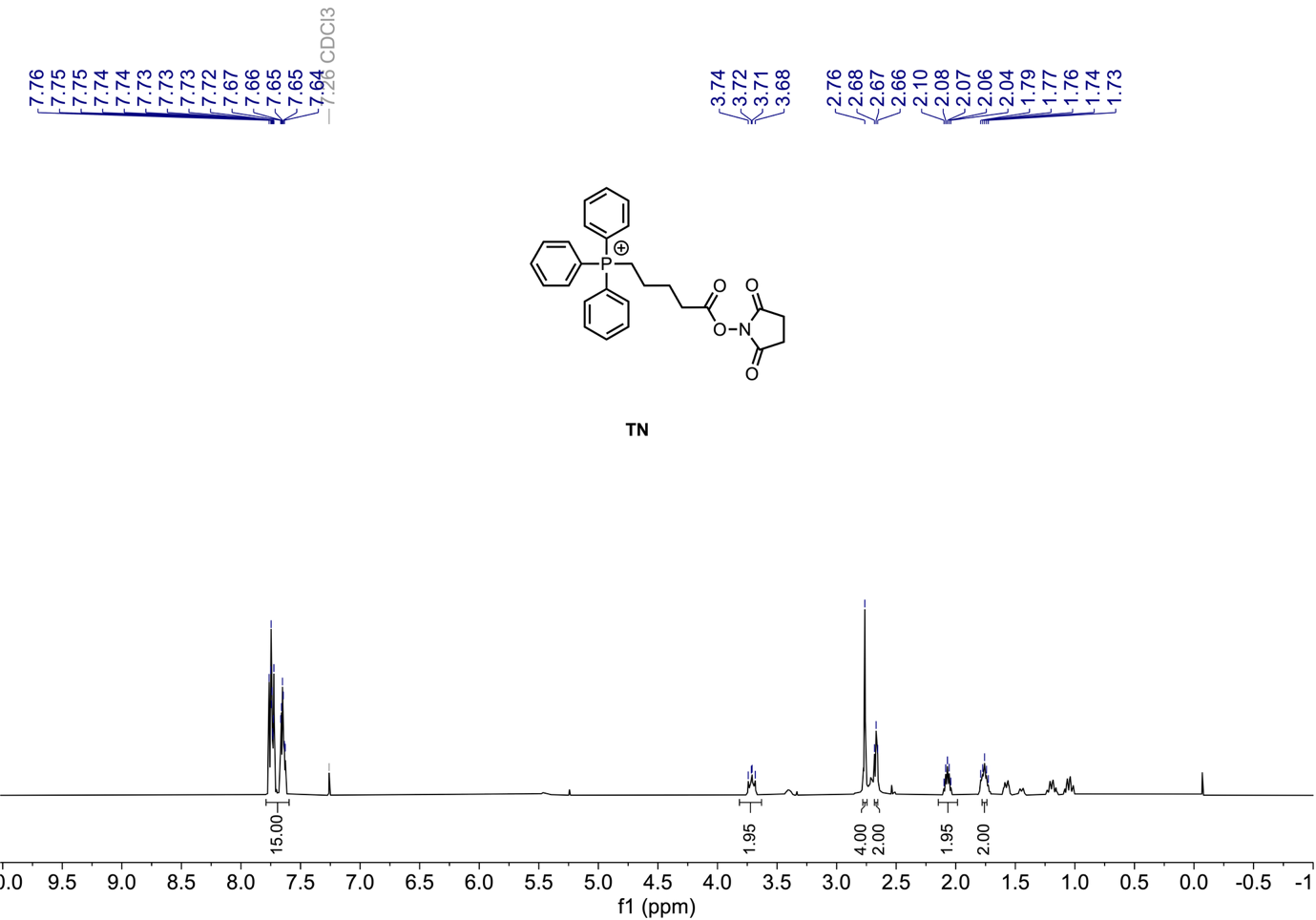
**

**Figure S29*.*** ^1^H NMR spectrum of compound **TN**.

**
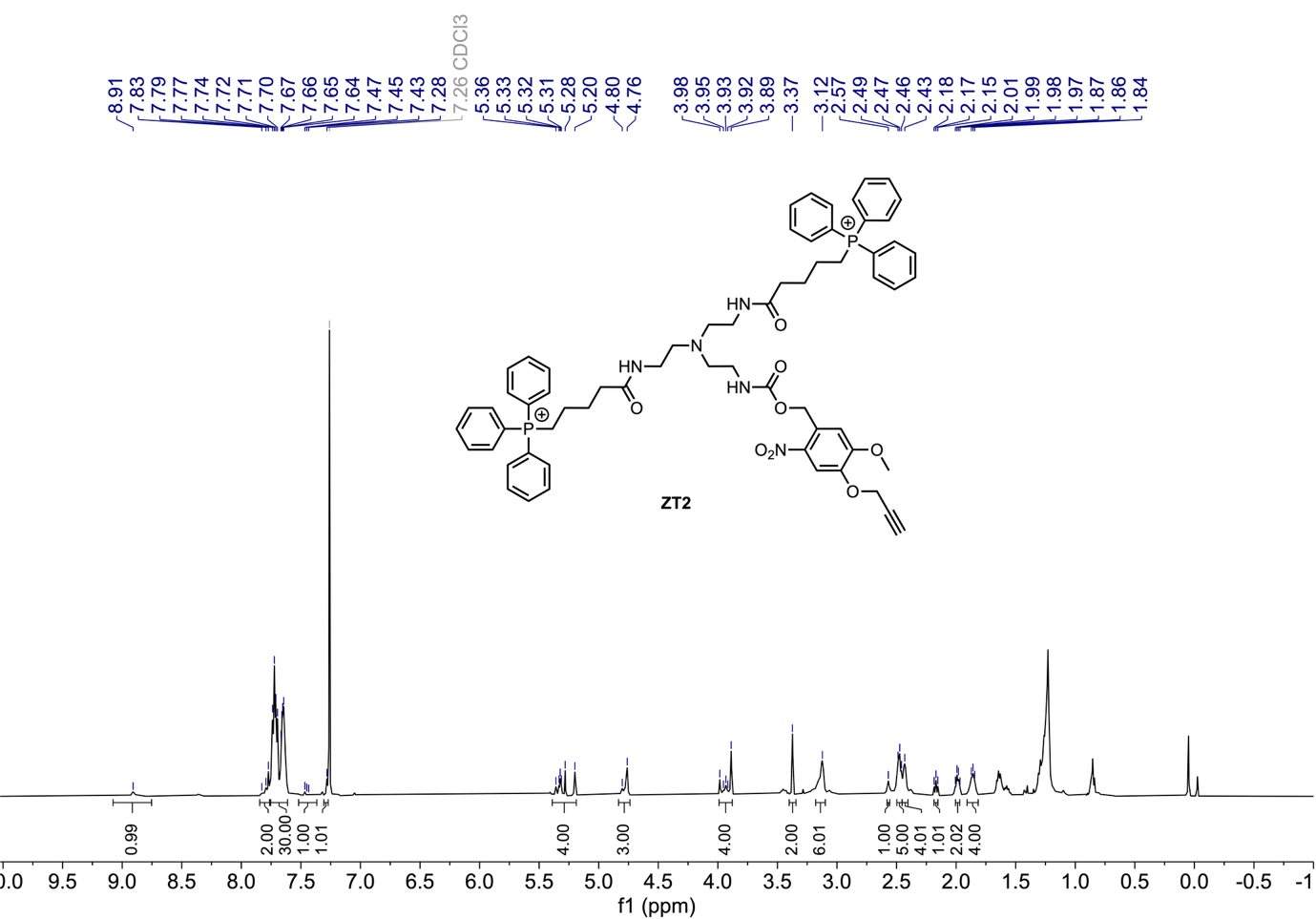
**

**Figure S30*.*** ^1^H NMR spectrum of compound **ZT2**.

**
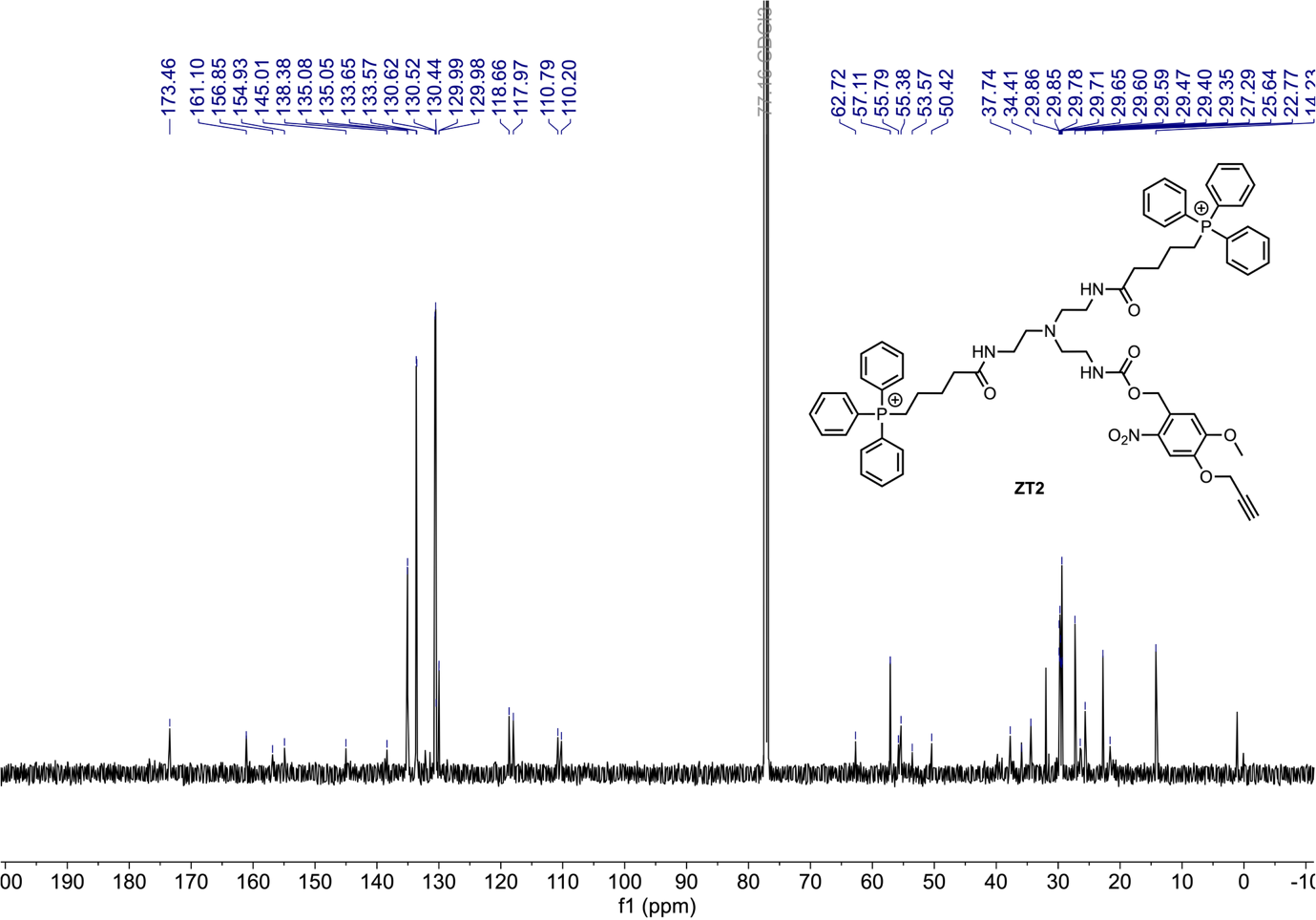
**

**Figure S31*.*** ^13^C NMR spectrum of compound **ZT2**.

**
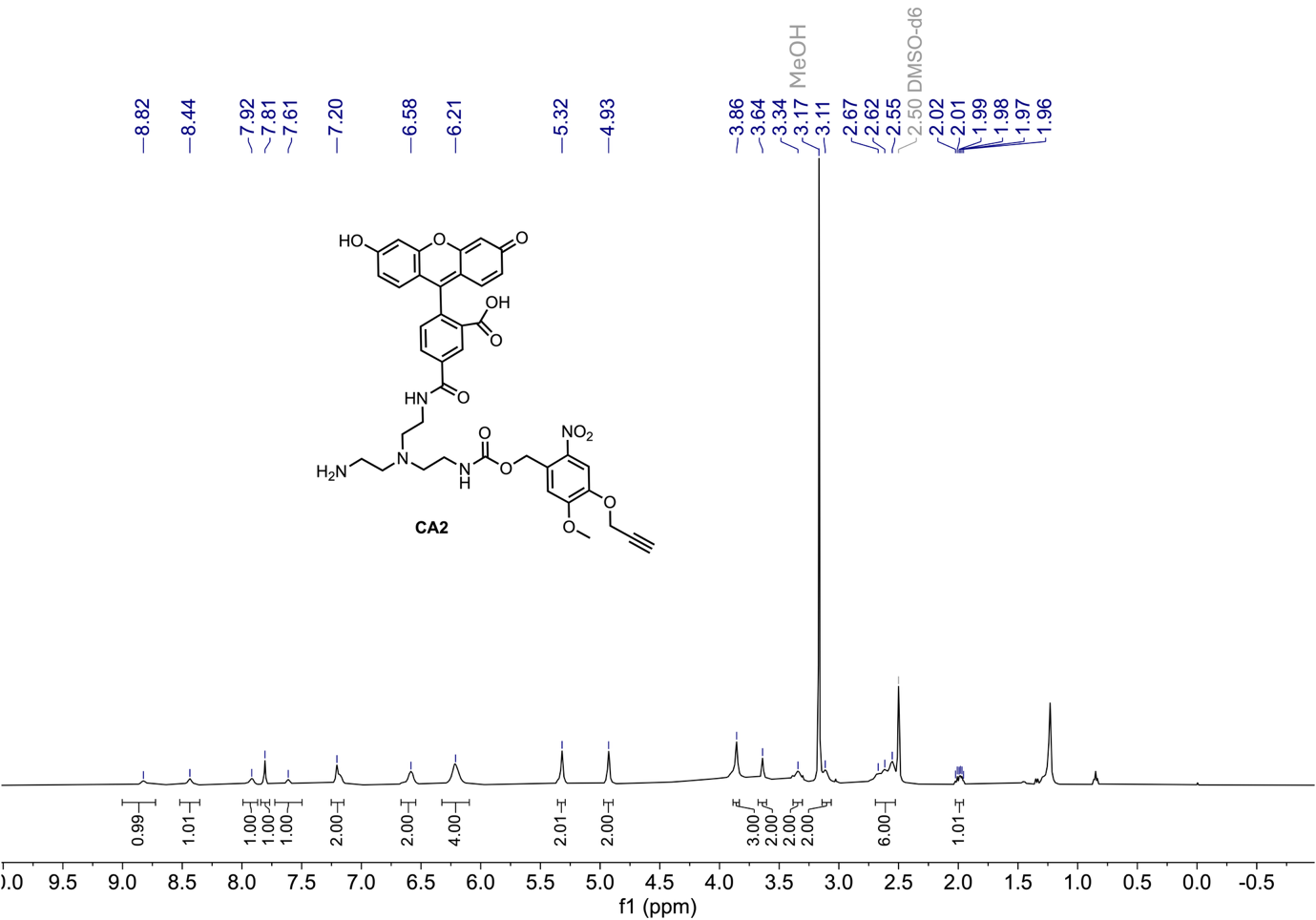
**

**Figure S32*.*** ^1^H NMR spectrum of compound **CA2**.


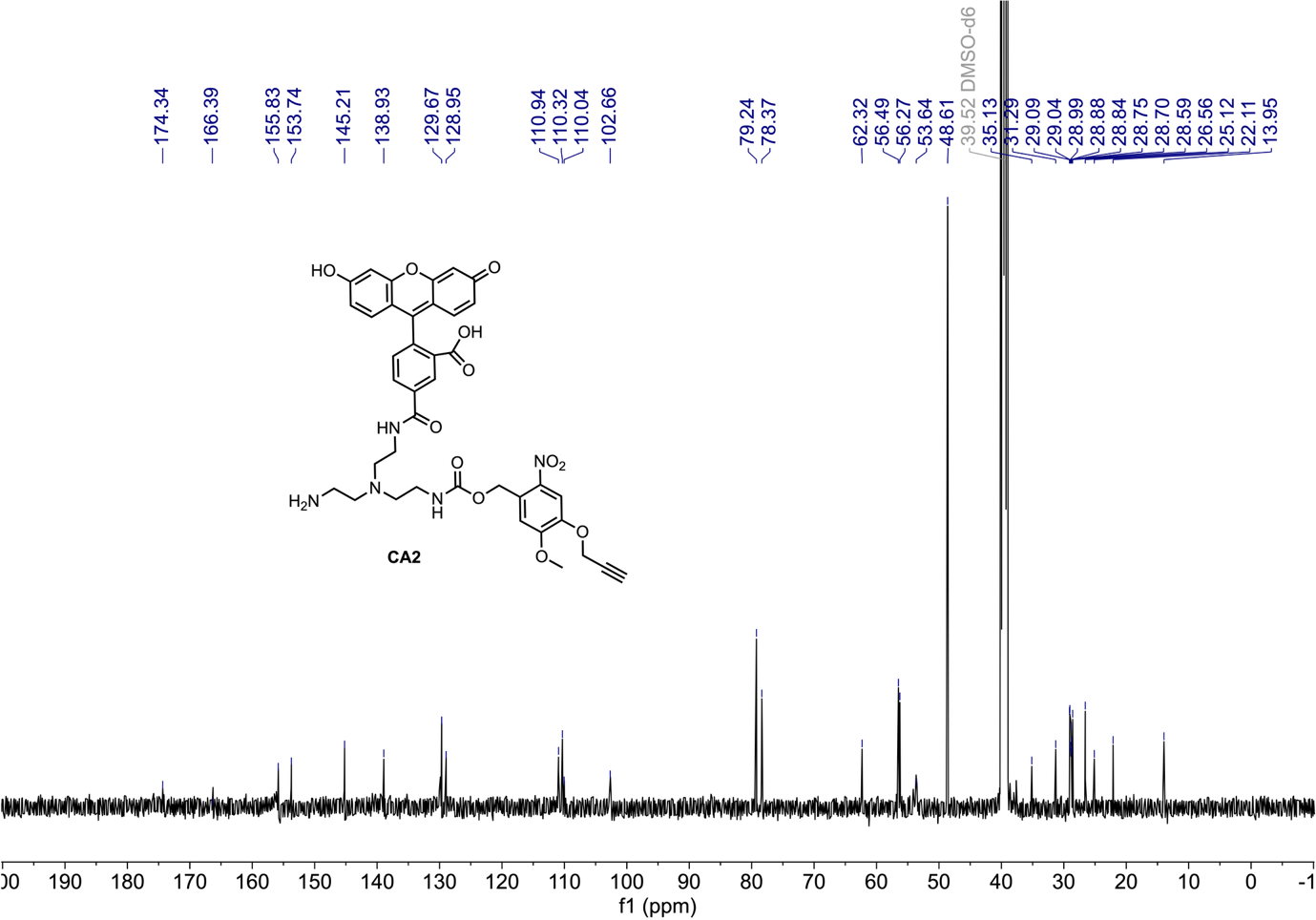


**Figure S33*.*** ^13^C NMR spectrum of compound **CA2**.


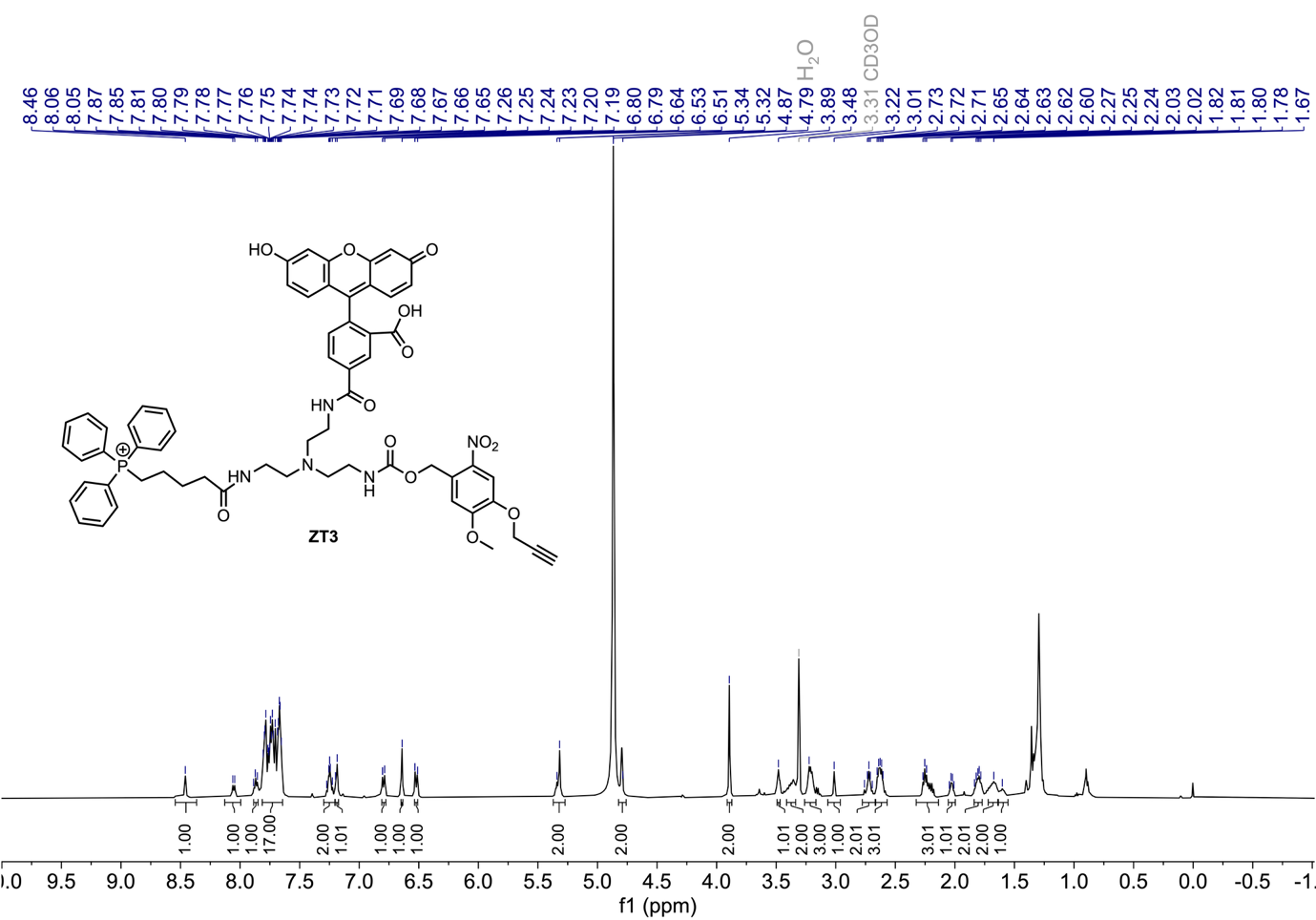


**Figure S34*.*** ^1^H NMR spectrum of compound **ZT3**.

**
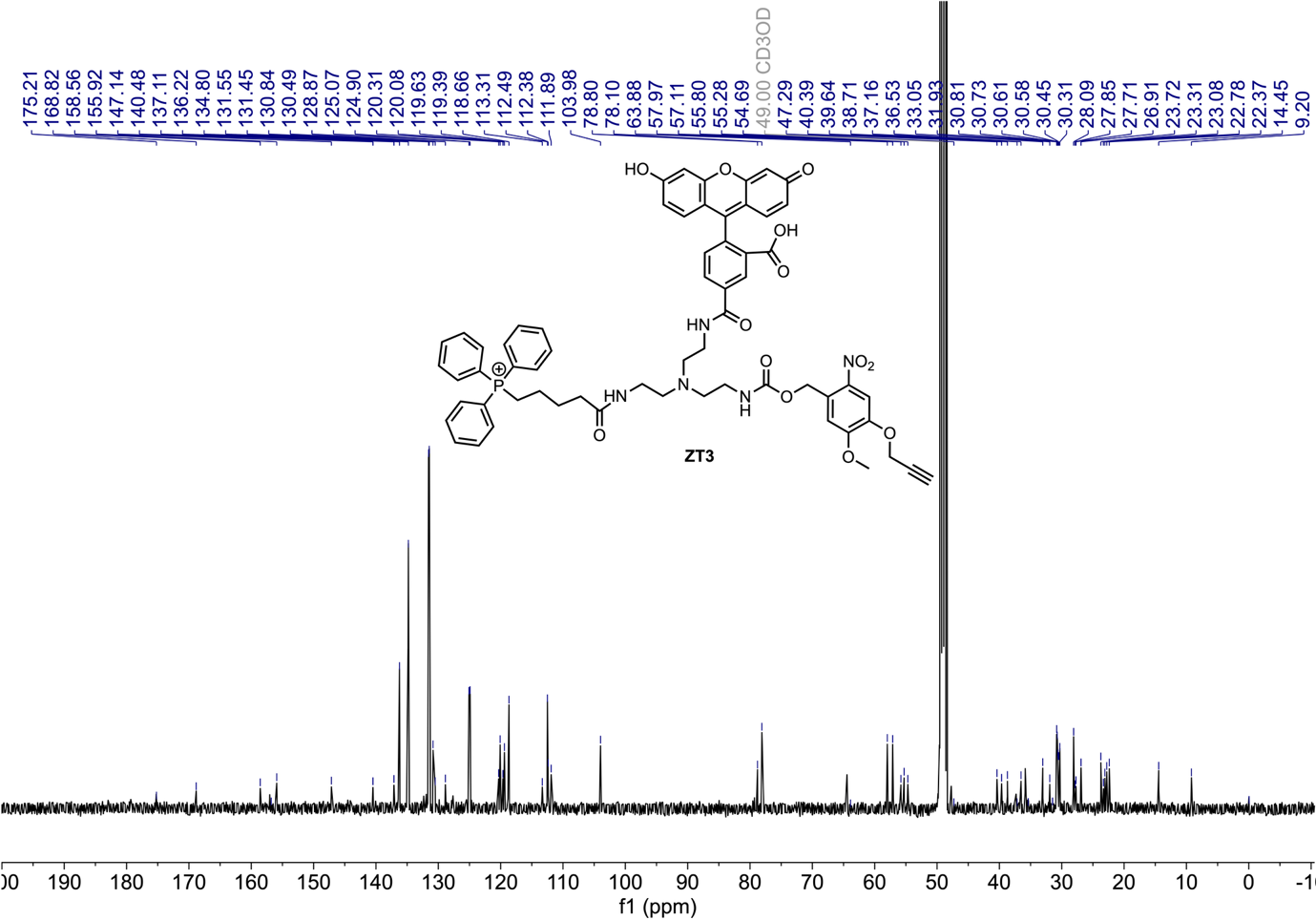
**

**Figure S35*.*** ^13^C NMR spectrum of compound **ZT3**.

**
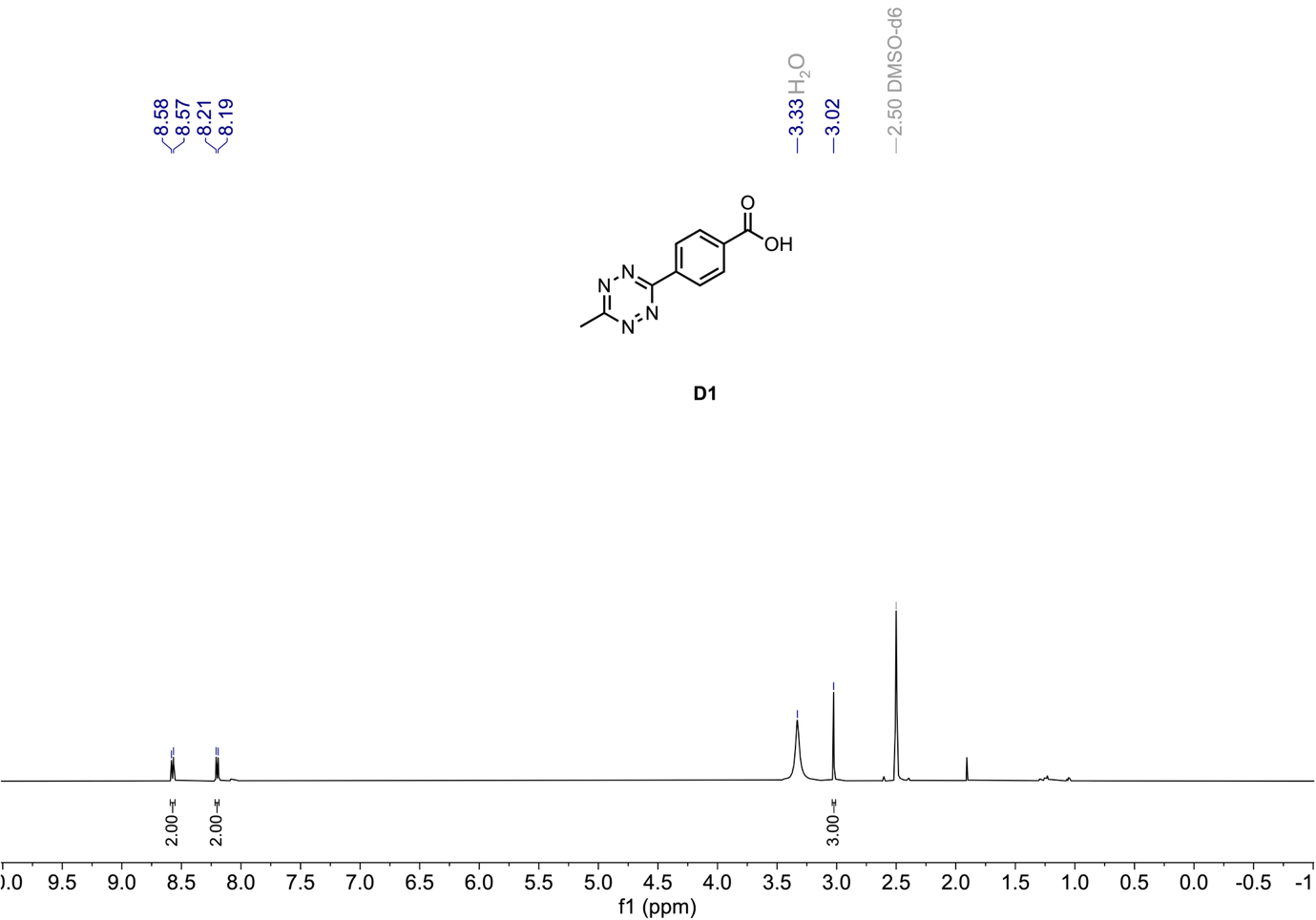
**

**Figure S36*.*** ^1^H NMR spectrum of compound **D1**.

**
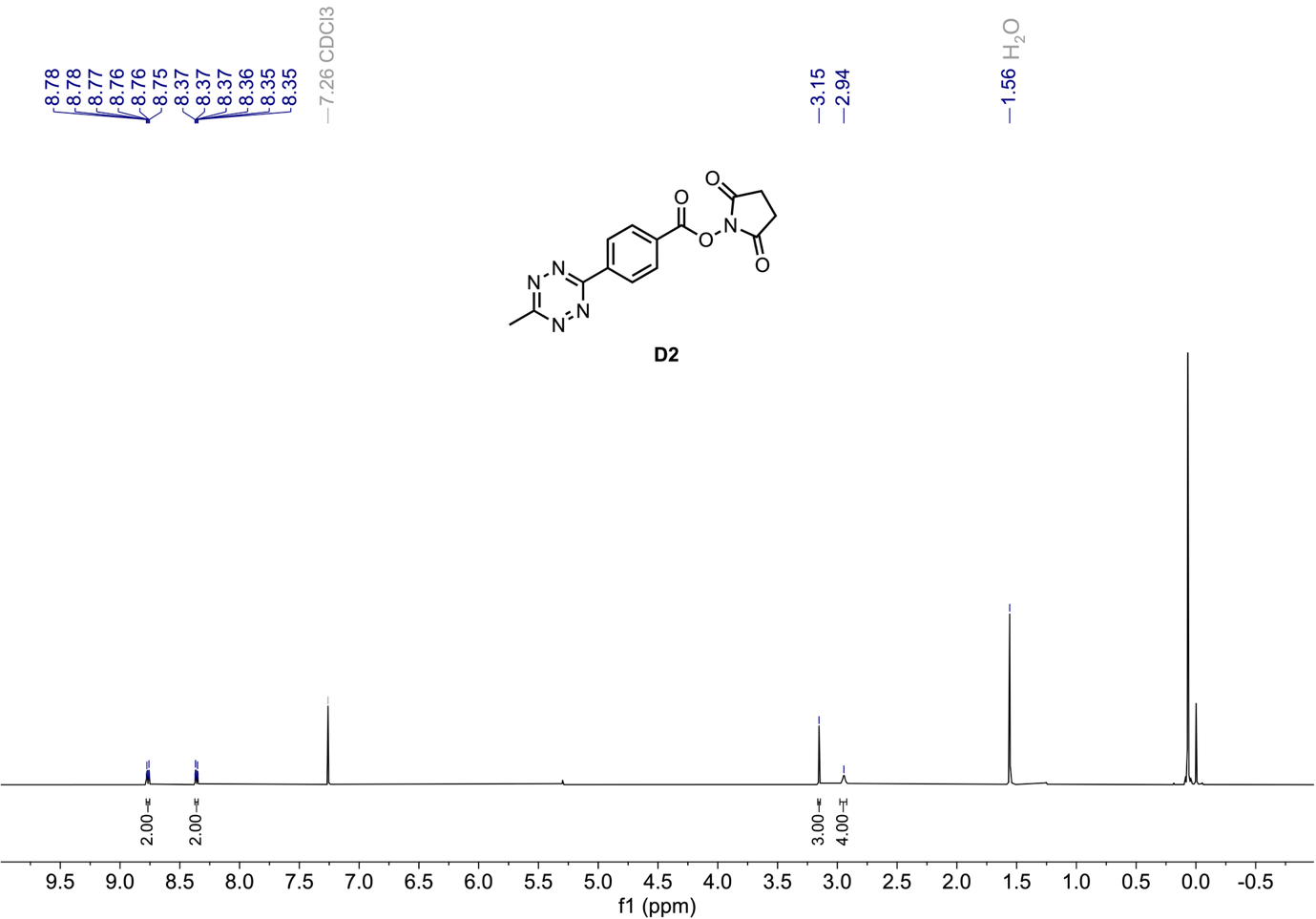
**

**Figure S37*.*** ^1^H NMR spectrum of compound **D2**.

**
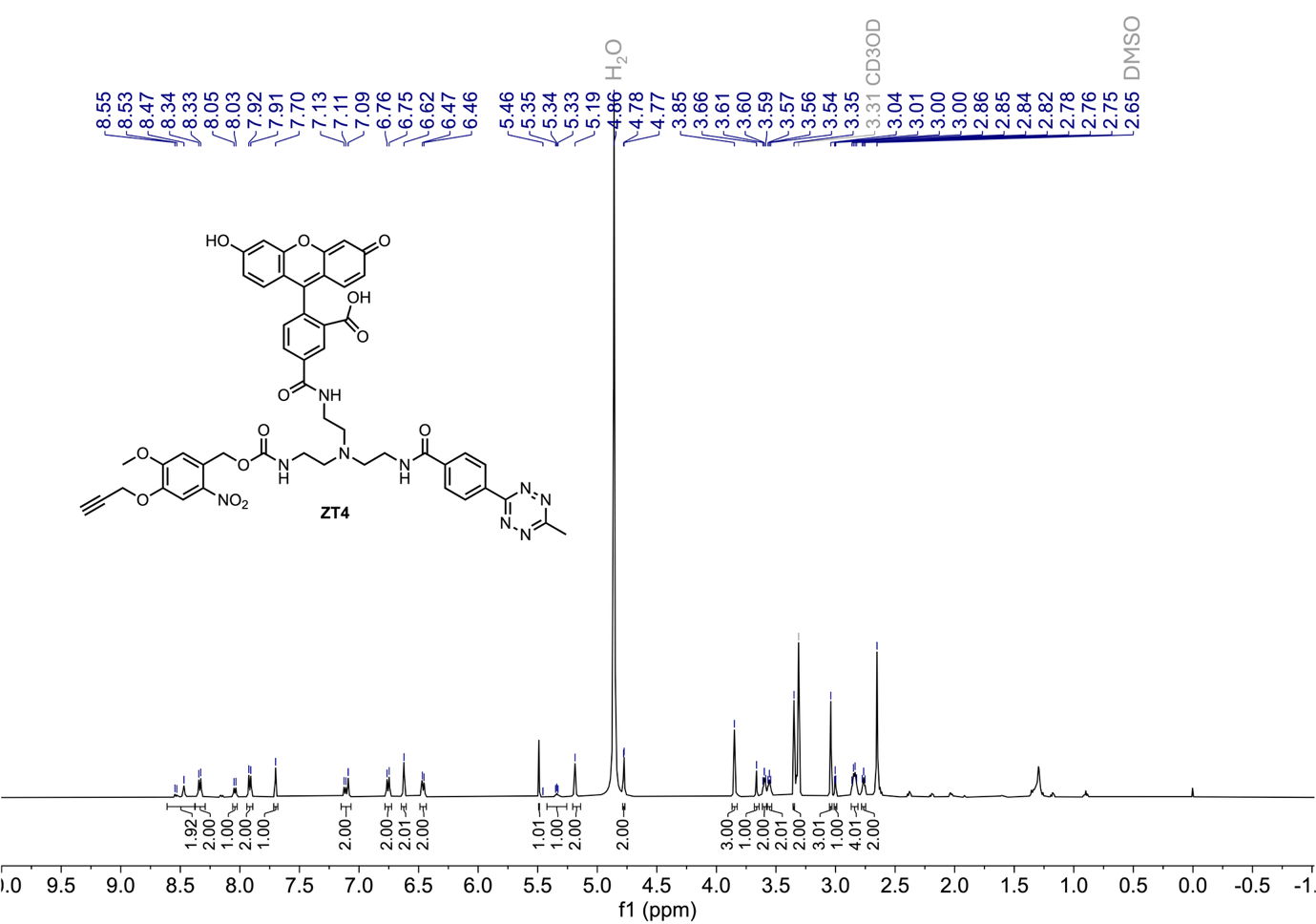
**

**Figure S38*.*** ^1^H NMR spectrum of compound **ZT4**.

**
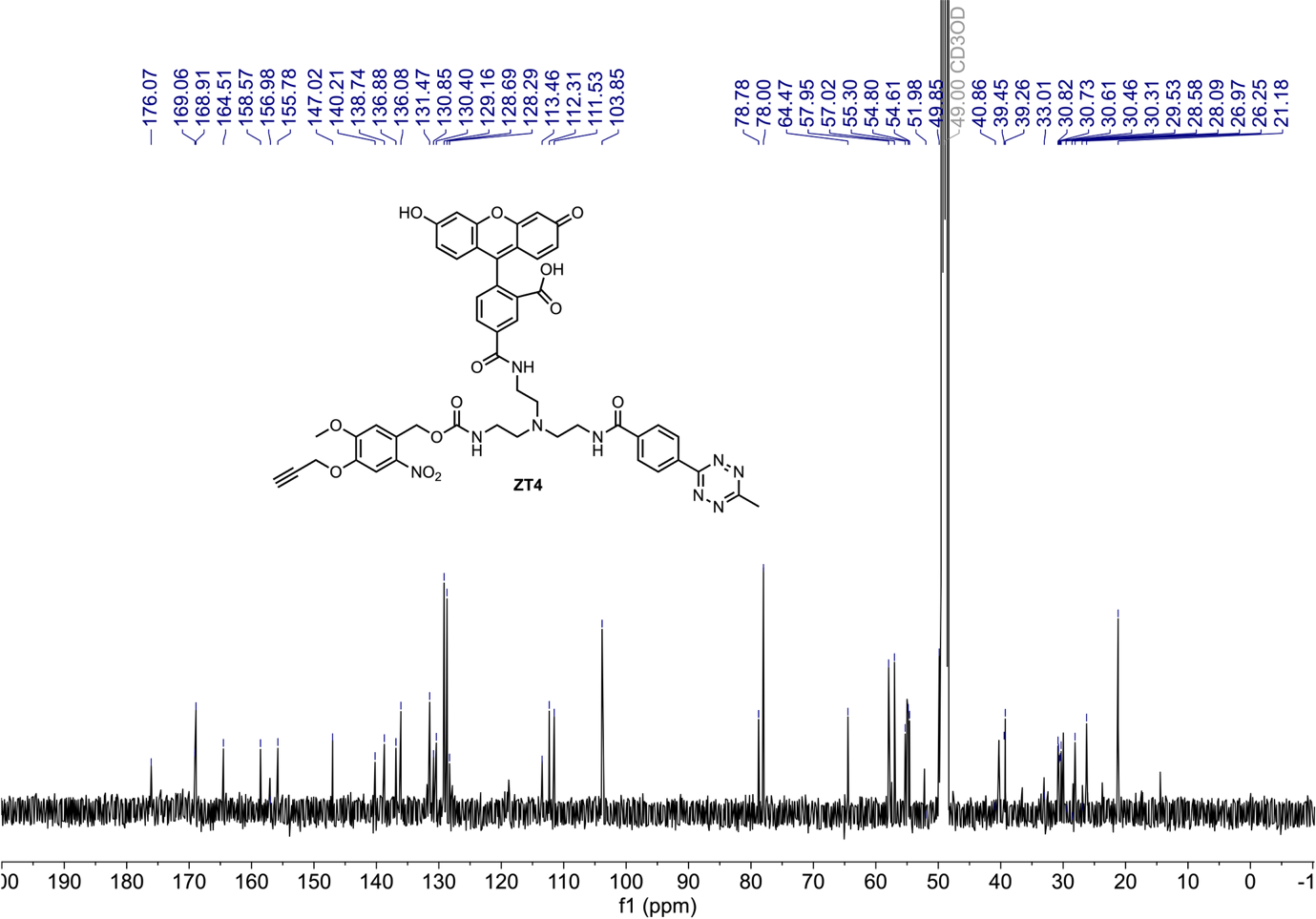
**

**Figure S39.** ^13^C NMR spectrum of compound **ZT4**.

**
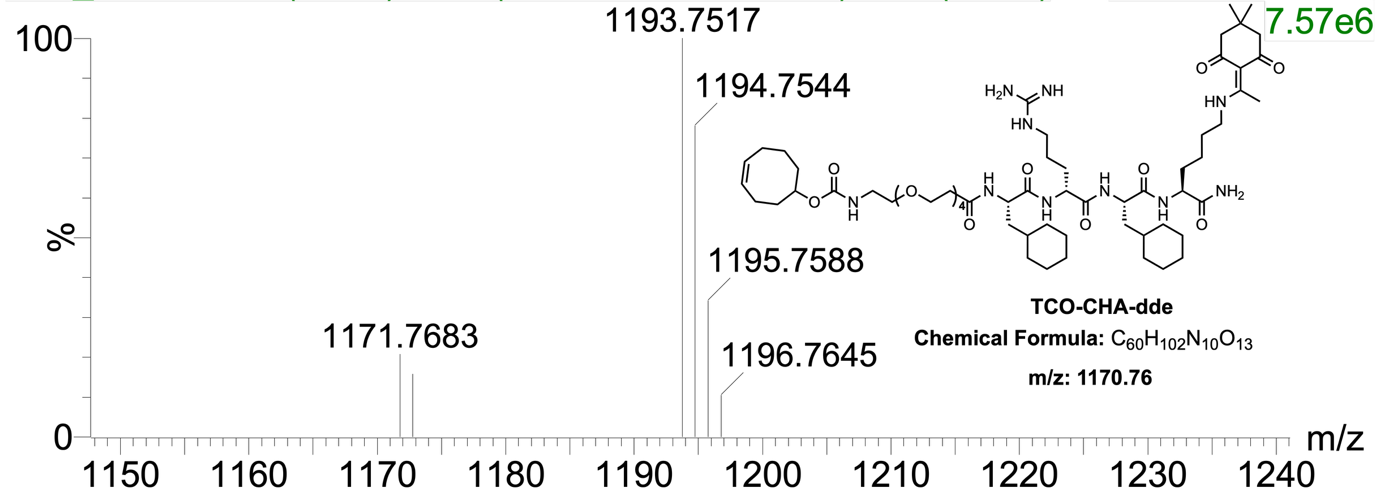
**

**Figure S40*.*** HRMS spectrum of compound **TCO-CHA-dde**.

**
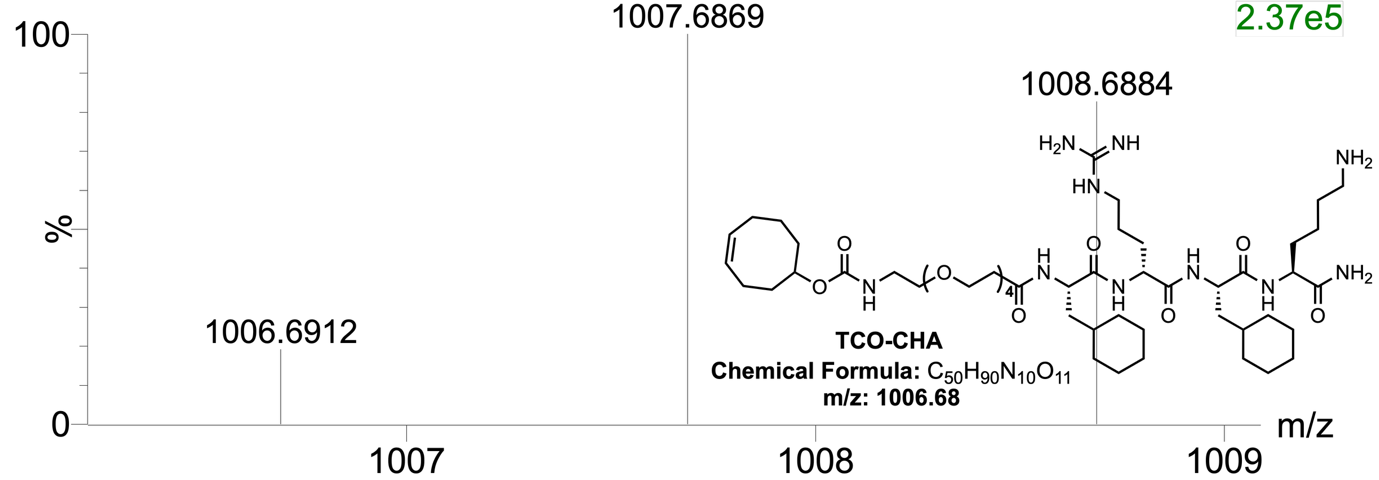
**

**Figure S41*.*** HRMS spectrum of compound **TCO-CHA**.

**5. References**

[1] N. R. Sims, M. F. Anderson, *Nat. Protoc.* **2008**, *3*, 1228-1239.

[2] C. Frezza, S. Cipolat, L. Scorrano, *Nat. Protoc.* **2007**, *2*, 287-295.

[3] J. M. Preble, C. A. Pacak, H. Kondo, A. A. MacKay, D. B. Cowan, J. D. McCully, *J. Vis. Exp.* **2014**, e51682.

[4] T. Saha, C. Dash, R. Jayabalan, S. Khiste, A. Kulkarni, K. Kurmi, J. Mondal, P. K. Majumder, A. Bardia, H. L. Jang, S. Sengupta, *Nat. Nanotechnol.* **2022**, *17*, 98-106.

[5] V. S. P. R. Lingam, R. Vinodkumar, K. Mukkanti, A. Thomas, B. Gopalan, *Tetrahedron Lett.* **2008**, *49*, 4260-4264.

[6] J. Yu, Y. Chen, Y.-H. Zhang, X. Xu, Y. Liu, *Org. Lett.* **2016**, *18*, 4542-4545.

[7] M. S. Padilla, C. A. Farley, L. E. Chatkewitz, D. D. Young, *Tetrahedron Lett.* **2016**, *57*, 4709-4712.

[8] B. Peng, C. Yu, S. Du, S. S. Liew, X. Mao, P. Yuan, Z. Na, S. Q. Yao, *ChemBioChem* **2018**, *19*, 986-996.

[9] C. Yu, L. Qian, M. Uttamchandani, L. Li, S. Q. Yao, *Angew. Chem. Int. Ed.* **2015**, *54*, 10574-10578.
